# Formation and Structural Characterization of Benzoxa- and Thienoxaphosphaborinines

**DOI:** 10.1021/acs.joc.5c00501

**Published:** 2025-05-21

**Authors:** Krzysztof Nowicki, Maja Grądecka, Patrycja Kurasz, Paweł A. Wieczorkiewicz, Damian Dąbrowski, Krzysztof Durka, Sergiusz Luliński

**Affiliations:** Faculty of Chemistry, 49566 Warsaw University of Technology, Noakowskiego 3, 00-664 Warsaw, Poland

## Abstract

Benzoxa- and thienoxaphosphaborinines, a new group of six-membered boron–phosphorus heterocycles, are reported. Their synthesis involved aromatic lithiation of appropriate 2-aryl-6-butyl[1.3.6.2]­dioxazaborocans, followed by treatment with PhPCl_2_ and subsequent hydrolysis giving rise to (2-(phenylhydrophosphoryl)­aryl)­boronic acids. These intermediates exhibit nucleophilic character and undergo readily condensation reactions with aldehydes and ketones giving rise to target products. The mechanism of the latter reaction was studied by means of DFT calculations showing that activation of the carbonyl partner occurs through the formation of the hydrogen bond with the B­(OH)_2_ group rather than through an interaction with the Lewis acid boron atom due to a plausible FLP-like behavior. The molecular structures of a few derivatives were determined by X-ray crystallography showing that in all cases, the molecules assemble through intermolecular BOH···O=P hydrogen bonds.

## Graphical abstract

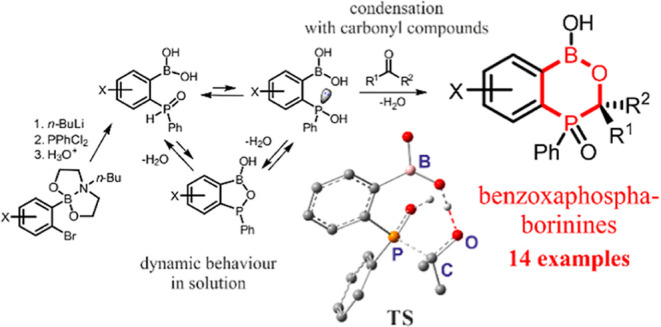

## Introduction

Recently, bifunctional aromatic compounds bearing boron- and phosphorus-based centers have attracted increasing interest due to their unique catalytic properties in activation of both nonpolar and polar molecules. Such systems are generally termed Frustrated Lewis Pairs (FLPs) as they comprise Lewis base phosphorus and Lewis acidic boron atom which are sterically hindered, thus precluding the formation of a standard dative bond between them.^1,2^ The reactivity of FLPs based on an ortho-disubstituted benzene core bearing adjacent B and P centers is specific as it may involve synergistic effects due to chelation of an approaching molecule which can be followed by structural rearrangements.^3,4^ It is worth noting that such systems may serve as useful starting materials for the preparation of various 5- and 6-membered boron–phosphorus heterocycles upon insertion of PhNCO, CH_2_O, difluorocarbene, and phosphinidenes (P–R) (Scheme 1).^5–10^ Their use as efficient bifunctional organocatalysts for Michael addition was based on initial insertion of enones.^11^

Our interest in heteroelement analogues of benzoxaboroles including ring-expanded derivatives^12^ has prompted us to study compounds comprising B–O–Si linkage based on naphthalene and biphenyl backbones^13^ as well as other borosiloxane systems obtained through transformations of formyl-^14^ and oxazolinyl-substituted benzosiloxaboroles.^15^ In this work, we present our studies on benzoxa- and thienoxaphosphaborinines (Scheme 1). These compounds can be regarded as benzoxaborole congeners where the boracyclic ring is expanded by insertion of phosphine oxide functionality. Their formation involved the cyclocondensation of ambiphilic phenylhydrophosphoryl-substituted arylboronic acids with carbonyl electrophiles: theoretical studies were performed to assess whether those intermediates may exhibit FLP-like reactivity. We also provide their general characterization, including crystallographic analysis of selected derivatives.

**Scheme 1:**
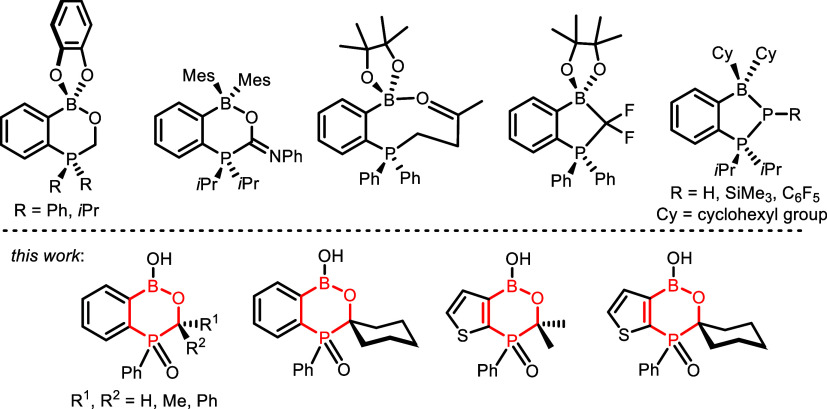
Comparison of Structures of Known Boracyclic Adducts Resulting from the FLP-like Activity of Ortho-Phosphinyl Arylboronates with Benzoxa- and Thienoxaphosphaborinines Reported in This Work

## Results and Discussion

### Synthesis

In the first step, (2-(phenylhydrophosphoryl)­phenyl)­boronic acid **5** was obtained by treatment of 2-(2′-bromophenyl)-6-butyl[1.3.6.2]­dioxazaborocan **1** with *n*-BuLi in THF at −78 °C,^16^ followed by rapid addition of PhPCl_2_ to a suspension of a generated aryllithium intermediate (Table 1) at the very low temperature (ca. −100 °C) in order to minimize the risk of the undesired side reaction, i.e., the nucleophilic substitution of the second P–Cl bond. After aqueous acidic hydrolysis, product **5** was isolated as a white solid that is soluble in polar organic solvents. It shows the ^31^P NMR resonance as a doublet (^1^ *J*_HP_ = 497.5 Hz) centered at 34.88 ppm in CDCl_3_ confirming the presence of the P–H bond. The spectral lines are broadened, which may indicate a dynamic behavior of **5**. The ^31^P NMR spectrum of **5** in DMSO-*d*_6_ shows a well-resolved doublet (^1^ *J*_PH_ = 506.8 Hz) of quartets centered at 30.9 ppm as the major signal. The latter splitting results from the coupling of the ^31^P nucleus with 2 aromatic protons of the phenyl group and another proton from the boronated aromatic ring (^3^ *J*_PH_ = 14.6 Hz), thus confirming the structure formulation. Another minor resonance (intensity of ca. 10% with respect to the major one) at 109.2 ppm was observed in DMSO-*d*_6_. Clearly, this points to the presence of a species featuring the trivalent P atom resulting from the prototropic tautomerization of the more abundant form of **5**, i.e., **5-P­(O)­H**. Such a process is a common feature of secondary phosphine oxides.^17–20^ For comparison, the ^31^P NMR chemical shift for the related compound Ph_2_POMe is 117.0 ppm.^21^ The signal assigned to the minor tautomer of **5** is significantly broadened (*h*_1/2_ ≈ 20 Hz) and thus lacks the multiplet structure resulting from the coupling with three protons located at the ortho positions of aromatic rings. We suppose that this could be attributed to a dynamic behavior of this species involving a reversible dehydrative cyclization giving rise to **5-POB** featuring the covalent P–O–B linkage (Scheme 2). It was corroborated by the ESI HRMS spectrum where a peak corresponding to a protonated **5-POB** species is observed at *m*/*z* = 229.0585 (Figures S80, S103, and S104). DFT structural optimization of **5-POB** (M06-2X^22^ level of theory with a 6-311++G­(d,p)^23^ basis set) indicates a fully covalent character of the P–O bond (*d*_PO_ = 1.69 Å) (Figure 1). To the best of our knowledge, we provide the first authentication for the existence of a “classical” benzoxaphosphaborole, i.e., a boracyclic species comprising three-coordinate B and P atoms covalently linked through the oxygen atom. Thus, **5-POB** can be regarded as a direct analogue of benzoxaborole. Further analogy with the latter compound is the ability to form rather strong dimers, as observed by the ESI MS/MS tandem mass spectrometry (positive ion mode) showing the peak of [2M + H]^+^ at *m*/*z* = 457 (Figure S104). Recently, related benzoxaphosphaborole systems with bulky aryl groups at boron atoms were reported. However, they feature five-coordinate P atoms with long P–O bonds (*d*_PO_ = 1.94 ^24^ and 2.05 Å^25^) which were interpreted at best to be on the borderline between ionic and covalent according to the NBO and QTAIM analyses. It should be noted that the analysis of mass spectrometry data of **5** revealed some peculiarities not observed by NMR spectroscopy, which may be attributed to compound activation under gas-phase conditions at higher temperatures. Thus, the ESI HRMS (positive ion mode) revealed solely the peak at *m*/*z* = 245.0533 (Figure S79), whereas the expected peak of [M + H]^+^ of **5** at *m*/*z* = 247.0690 was absent. A deeper investigation using ESI MS/MS tandem mass spectrometry revealed the presence of the expected deprotonated [M – H]^−^ at *m*/*z* = 245 (negative ion mode) and the protonated [M + H]^+^ at *m*/*z* = 247 (positive ion mode) forms of **5** (Figures S100–S102). However, peaks of higher intensities at *m*/*z* = 243 and 245, respectively, were found in both modes. Fragmentation pathways of −2 Da signals and their corresponding quasi-molecular ion counterparts are consistent with a formal loss of the H_2_ molecule from **5** (Figures S79, S100, S102). Therefore, forms [M – H_2_ – H]^−^ at *m*/*z* 243 and [M – H_2_ + H]^+^ at *m*/*z* 245 can be attributed to benzoxaphosphaborole species **5-P­(O)­OB**, i.e., the oxidized derivative of **5-POB** although the mechanism of its formation remains unclear as both dehydrogenation and oxidation pathways can be considered (Scheme 2).

**Table 1:**
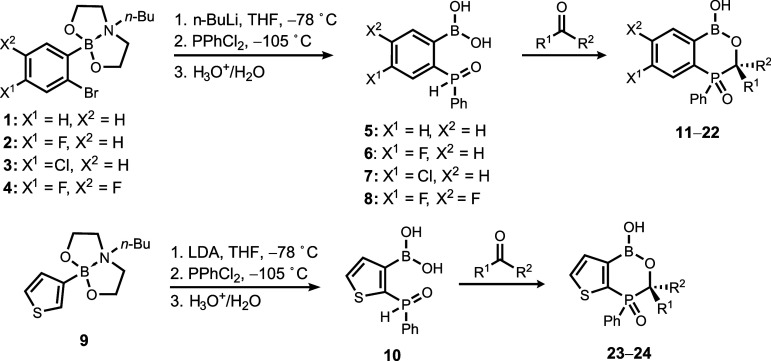
Synthesis of Functionalized Benzoxa- and Thienoxaphosphaborinines **11**–**24**

compound	X^1^	X^2^	R^1^, R^2^	yield/%
**11**	H	H	Me, Me	52
**12**	H	H	H, H	51
**13**	H	H	cyclohexane-1,1-diyl	39
**14**	H	H	Ph, H	64
**15**	F	H	Me, Me	26
**16**	F	H	H, H	66
**17**	F	H	cyclohexane-1,1-diyl	64
**18**	Cl	H	Me, Me	61
**19**	Cl	H	cyclohexane-1,1-diyl	41
**20**	F	F	Me, Me	41
**21**	F	F	H, H	80
**22**	F	F	cyclohexane-1,1-diyl	43
**23**			Me, Me	48
**24**			cyclohexane-1,1-diyl	58

**Scheme 2:**
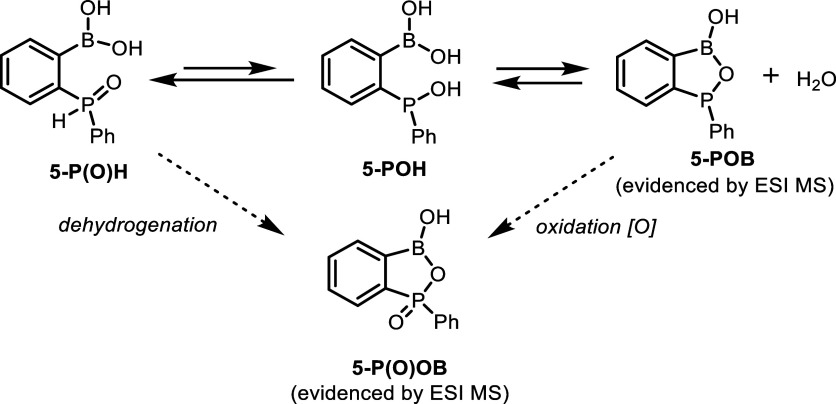
Postulated Equilibria between Various Forms of **5**

According to DFT calculations (CHCl_3_ solvent field), the P­(O)H tautomer (**5-P­(O)­H**) is only slightly more stable than **5-POH** (Δ*G* = 5.2 kJ·mol^–1^), which is consistent with the observed dynamic behavior of **5**. In addition, we have considered the existence of two conformers of **5-P­(O)­H** stabilized either by an intramolecular P=O···HO–B hydrogen bond interaction or a dative P=O → B interaction. The HB structure is more stable by 22.7 kJ·mol^–1^ and thus is predicted to dominate in solution. Three different conformers of the tautomer **5-POH**, characterized by P–OH···O­(H)–B, P–(H)­O···HO–B, or P···HO–B HBs, are less stable with respect to **5-P­(O)­H** by 5.2–11.9 kJ·mol^–1^. On the other hand, the standard Gibbs energy of the formation of **5-POB** from **5-P­(O)­H** is 8.3 kJ·mol^–1^. Thus, due to similar stabilities of **5-POH** and **5-POB** (equal to within a few kJ·mol^–1^), it can be concluded that these species should effectively equilibrate in solution (Figure 1). GIAO calculations of ^31^P NMR chemical shifts at the same level of theory with the CHCl_3_ solvent field of all studied forms of **5** are in general agreement with the conclusions derived from experimental spectra. Specifically, the ^31^P NMR chemical shift for **5-P­(O)­H** is 24 ppm, and for **5-POH**, the δ^31^P NMR value averaged over three different conformers is equal to 110 ppm, while in the case of **5-POB**, it is 117 ppm. Overall, the collected experimental data and theoretical calculations point to a dynamic behavior of **5** which seems also to be affected by a type of solvent and presence of water.

**Figure 1:**
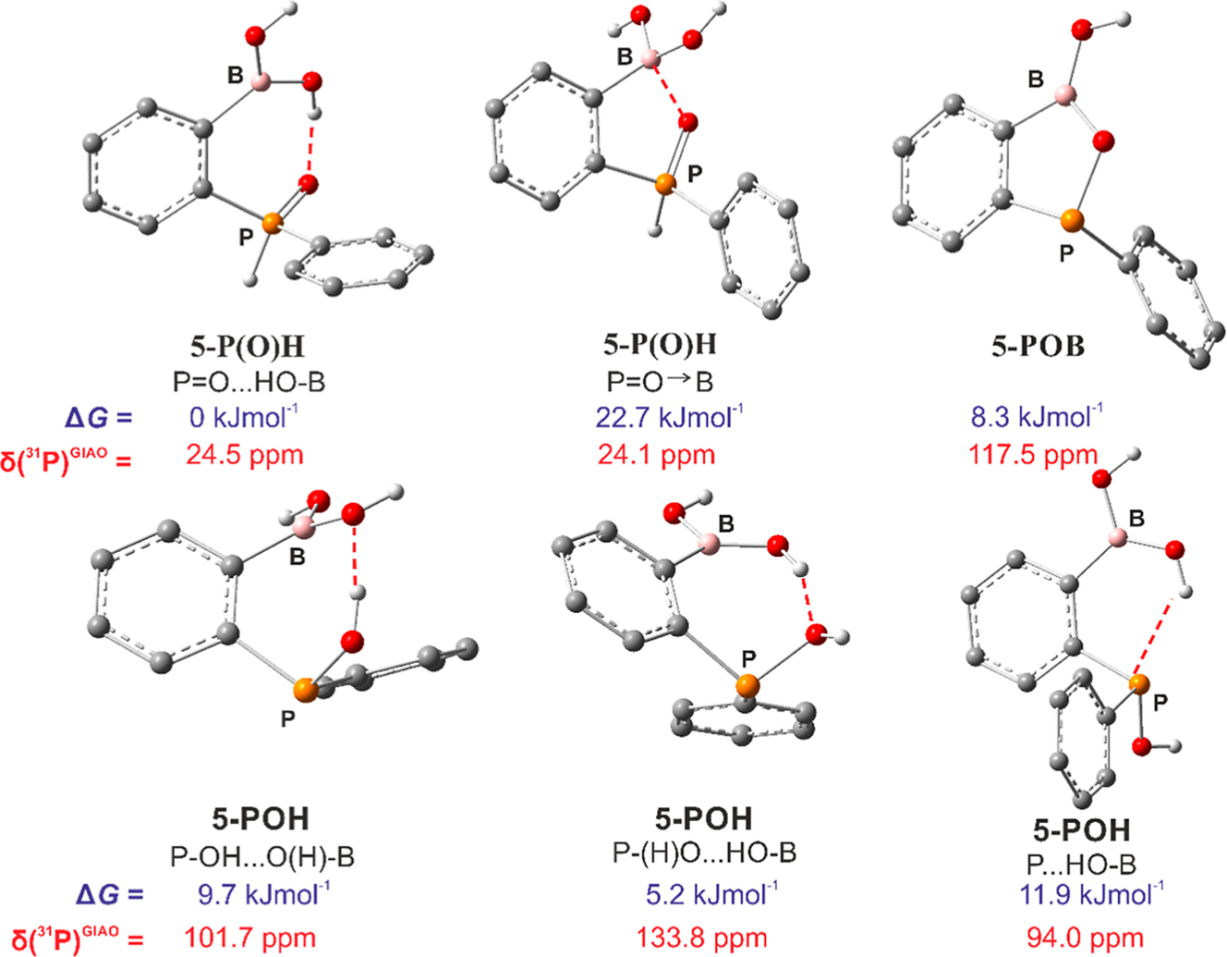
Various forms of **5**. Geometries were derived from theoretical calculations (M06-2X/6-311++G­(d,p)), relative free-enthalpy values (Δ*G*) are given with respect to the **5-P­(O)­H** form. Theoretical ^31^P NMR chemical shift values for each form have been additionally provided.

In addition, the ^11^B NMR spectrum of **5** in CDCl_3_ showed the major signal at 28.6 ppm, which is typical for a trigonal boron atom of the B­(OH)_2_ group. Under alkaline conditions (D_2_O + NaOH), the ^11^B NMR resonance at δ = 1.5 ppm confirms the presence of a tetrahedral anionic boronate group. It should be noted that **5** was contaminated by a significant proportion (ca. 15 mol %) of phenylphosphinic acid Ph­(O)­(H)­OH, presumably resulting from the hydrolysis of unreacted PhPCl_2_, as indicated by the MS spectrum showing a peak at *m*/*z* = 141 (negative ion mode) and ^31^P NMR spectra showing a minor doublet centered at 21.8 ppm (^1^ *J*_HP_ = 570 Hz) in CDCl_3_ or a doublet of triplets at 21.9 ppm (^1^ *J*_HP_ = 548 Hz, ^3^ *J*_HP_ = 14.1 Hz) in DMSO-*d*_6_.^26^ Since boronic acids are incompatible with chromatography purification, we attempted to purify **5** through washing or crystallization, but those attempts failed, and thus we used a crude material for subsequent reactions. The synthesis of halogenated (2-(phenylhydrophosphoryl)­phenyl)­boronic acids **6–8** was accomplished using the approach described for **5** starting with their respective precursors **2**–**3** ^27^ and **4**.^28^ The thiophene analogue **10** was obtained by deprotonative lithiation of a boronated thiophene precursor **9** with LDA according to the reported procedure.^29^ As for **5**, the isolation of well-defined and pure (2-(phenylhydrophosphoryl)­aryl)­boronic acids **6–8** and **10** proved troublesome but their formation was unambiguously confirmed by HRMS data (Figures S81–S83). For **10**, however, the peak attributable to the [M + H]^+^ at 253.0252 *m*/*z* ion is accompanied by a more abundant signal of 251.0096 *m*/*z* (−2 Da), most probably the [M – H_2_ + H]^+^ ion (Figure S84), which resembles the behavior of **5**. An intense peak (*m*/*z* = 235.0148) of protonated thienoxaphosphaborole species **10-POB** is also present (Figure S85). As for **5**, ^1^H and ^13^C NMR spectra of **6**–**8** and **10** in DMSO-*d*_6_ show broadened signals, indicating the presence of various forms coexisting in dynamic equilibria. This is in line with the respective ^11^B NMR spectra in DMSO-*d*_6_ (Figures S19, S24, S29, S34) showing two resonances at ca. 27–30 and 3–6 ppm at varying proportions which indicates the presence of both three- and four-coordinate boron species. Furthermore, ^31^P NMR spectra of **6**–**8** in DMSO-*d*_6_ show minor resonances at ca. 103–104 ppm, which can be assigned to P–OH tautomers or respective benzophosphoxaboroles, both types of structures bearing the trivalent P atom. In contrast, the ^31^P NMR spectra of the thiophene derivative **10** do not show the presence of analogous P­(III) species irrespective of the solvent used (CDCl_3_ or DMSO-*d*_6_), which indicates that the type of an aromatic ring has a significant effect on the tautomerization and subsequent equilibria.

In the next step, we subjected compounds **5–8** and **10** to condensation reactions with selected aldehydes and ketones. We found that **5** reacts readily with acetone when dissolved in this solvent at room temperature giving rise to benzoxaphosphaborinine derivative **11** which precipitates from a solution as a white solid during several minutes. The ^1^H NMR spectrum in CDCl_3_ shows two doublets of diastereotopic methyl groups at 1.62 (^3^ *J*_PH_ = 12.0 Hz) and 1.34 ppm (^3^ *J*_PH_ = 12.9 Hz), which confirms the attachment of the Me_2_C moiety at the stereogenic phosphorus center. The molecular structure of **11** was confirmed by single-crystal X-ray diffraction analysis (Figure 2). It should be noted that analogous reactions of diphenyl phosphine oxide with acetone and benzaldehydes were reported to give respective carbinol adducts.^18,30–32^ The formation of boracyclic structure **11** is thermodynamically driven by the release of the water molecule. It is confirmed by the lack of separate resonances of protons of B­(OH)_2_ and carbinol groups in the ^1^H NMR spectrum in CDCl_3_. There is only a broadened resonance of 1 proton at 5.63 ppm which can be assigned to the B–OH group.

**Figure 2:**
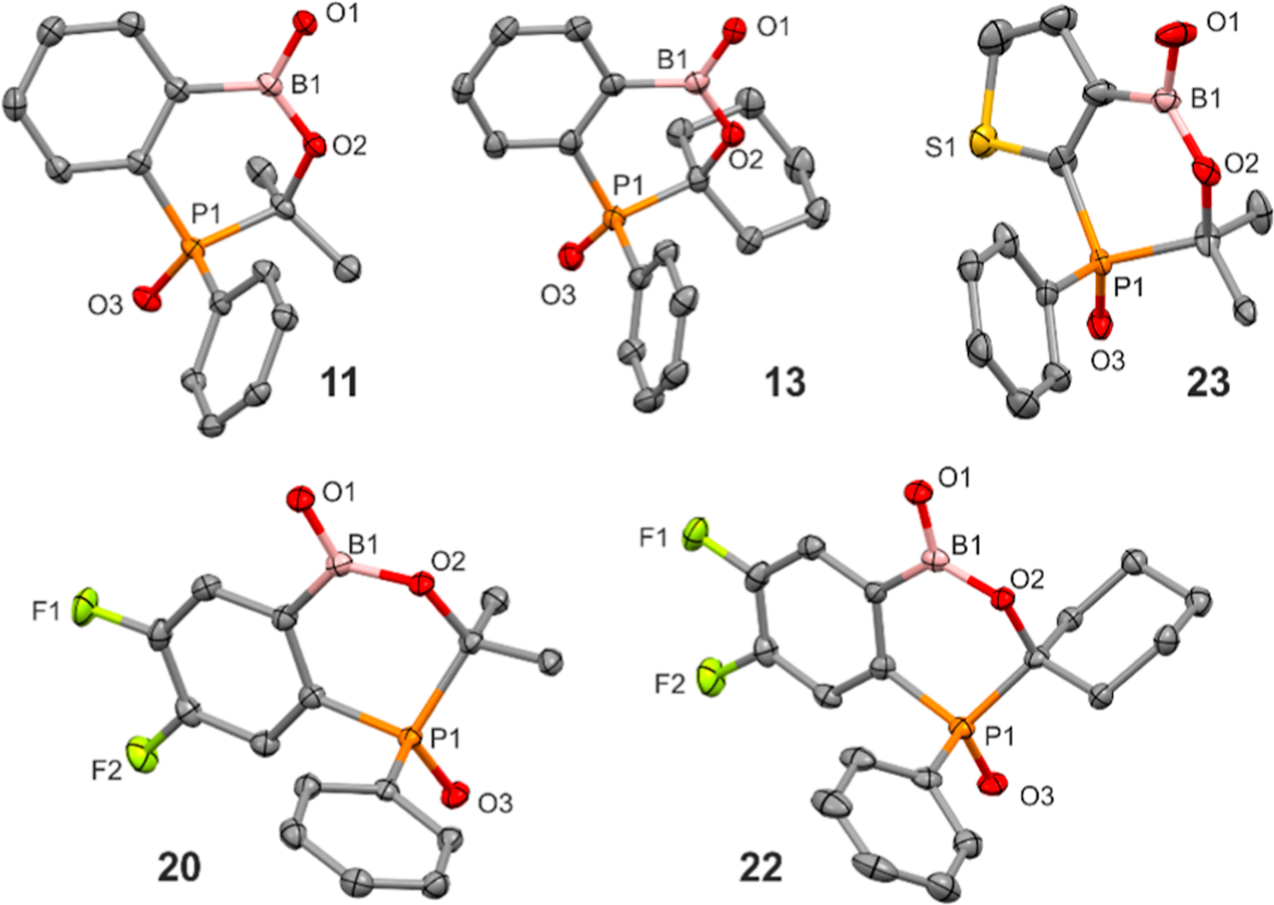
Molecular structures of benzoxaphosphaborinines **11**, **13**, **20**, **22**, and **23**. Ellipsoids drawn at the 50% probability level. Hydrogen atoms are omitted for clarity.

The mechanism of the reaction of **5** with acetone was examined by DFT calculations performed at the M06-2X/6-311++G­(d,p) level of theory. It should be noted that the minor tautomer **5-POH** is the reactive species due to the presence of a nucleophilic P atom. We have considered a pathway involving B–OH···O=CMe_2_ hydrogen-bond interaction preceding the formation of a transition state (HB-induced pathway, Figure 3). The mechanism assumes the simultaneous proton transfer from the P–OH group to the B–OH group and from B–OH to Me_2_C–O^–^ (blue arrows in Figure 3—TS-HB). The alternative mechanism assumes that the carbonyl group coordinates to the boron atom through dative C=O → B interaction, while the lone pair of the P atom interacts with the electrophilic carbon atom. This mechanism can be classified as a Frustrated Lewis Pair activation (FLP-like pathway). In both cases, the carbonyl group becomes more electrophilic, which facilitates an attack by the nucleophilic P atom of the **5-POH** tautomer. The computed energies of transition states support the HB-induced pathway (Δ*G*(HB-TS) = 31.9 kJ·mol^–1^; Δ*G*(FLP-TS) = 59.4 kJ·mol^–1^; acetone solvent field. In line with these observations, the P···C­(O) distance in HB-TS (*d*_P···C_ = 1.915 Å) is shorter with respect to the FLP-TS (*d*_P···O_ = 2.565 Å). Furthermore, the direct product of the HB-induced addition is much more stable compared with that formed through the FLP-like pathway. In addition, we have considered another FLP-like pathway involving **5-POB** species. However, the calculations of such a process did not converge, resulting in a state with the O atom of the acetone molecule only weakly interacting with the boron atom. To evaluate how strongly the boronic group activates the addition process, the Gibbs free energy of the transition state of the reaction between diphenylphosphine oxide Ph_2_P­(O)­(H) and acetone was calculated. The resulting value of 53.8 kJ·mol^–1^ (with respect to the Ph_2_POH tautomer) is higher than that for the analogous reaction of **5**. This confirms the synergistic effect of the boronic group and phosphorus center in the condensation process in the latter case.

**Figure 3:**
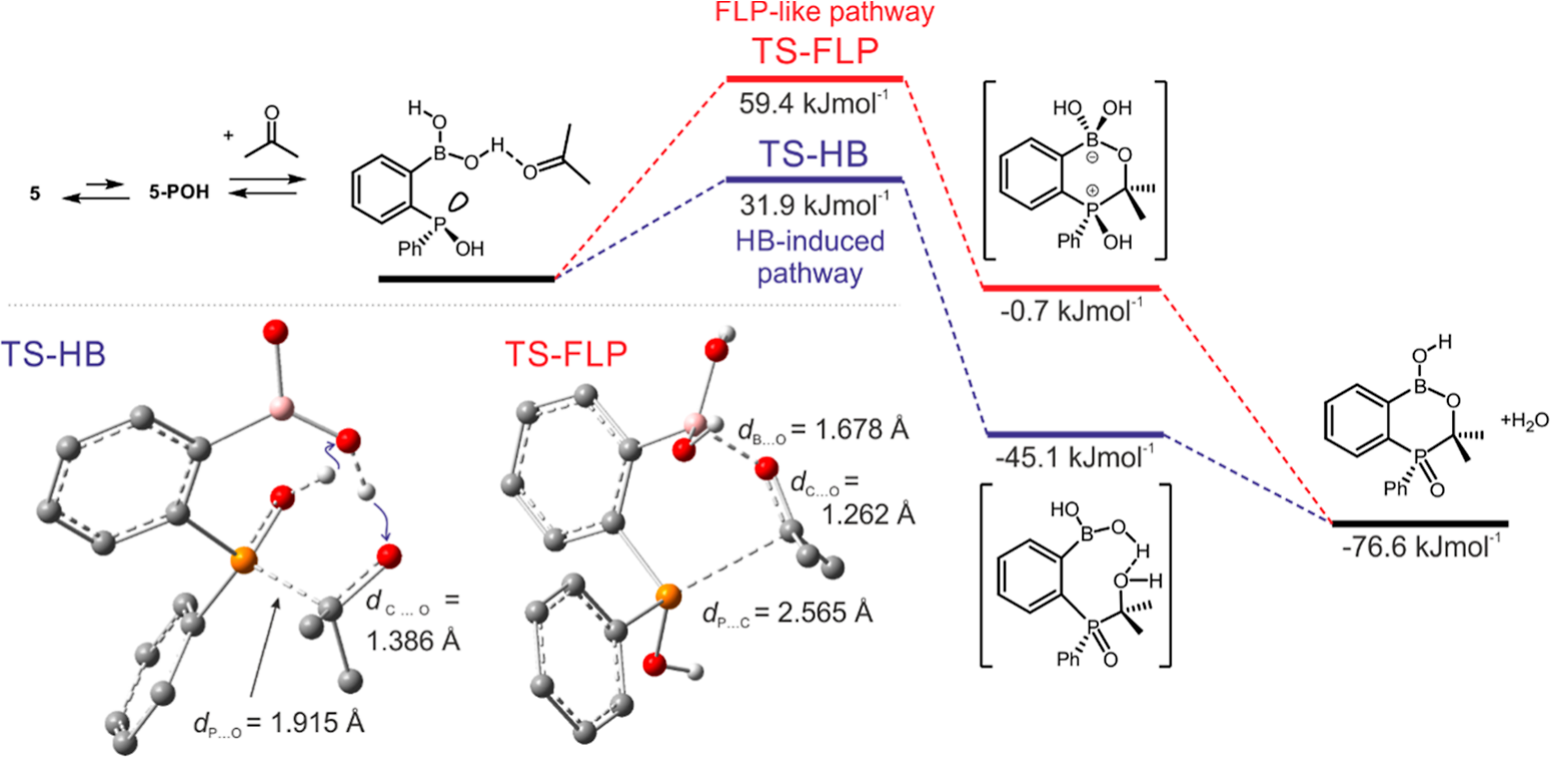
Proposed mechanism of the formation of **11**.

Compound **5** was also treated with other carbonyl substrates such as formaldehyde, cyclohexanone, and benzaldehyde, to give respective products **12–14** (Table 1). However, reactions with less-reactive electrophiles including sterically congested ketones (2,2,4,4-tetramethylpentan-3-one and benzophenone), ethyl acetate, and acetonitrile failed. The structures of **12–14** were confirmed by HRMS and multinuclear NMR spectroscopy data. The ^1^H NMR spectrum of **12** (Figure S40) shows two characteristic doublets of doublets of diastereotopic hydrogen atoms due to homonuclear geminal coupling constant |^2^ *J*_HH_| = 14.5 Hz, whereas heteronuclear coupling constants |^2^ *J*_HP_| are 8.1 and 5.0 Hz for signals at 4.57 and 4.69 ppm, respectively. For **14**, the formation of two diastereoisomers should be considered (Scheme 3). Indeed, two signals are observed in the ^31^P NMR spectrum of compound **14**. The major one at 24.1 ppm can be assigned to the **14**-*anti* diastereomer, whereas the minor resonance of **14**-*syn* appears at 21.4 ppm. The formation of the **14**-*anti* is preferred due to the lack of unfavorable steric interactions of phenyl groups which, in turn, destabilize the **14**-*syn* structure. The ^1^H NMR spectrum of **14** (Figure S46) shows a doublet of the (P)­CH proton at 5.82 ppm (|^2^ *J*_HP_| = 9.2 Hz) as well as a broadened singlet at 5.56 ppm in a ratio of 2:1. It seems that the doublet can be assigned to the more stable and abundant **14**-*anti* diastereomer. Presumably, **14**-*syn* is prone to reversible hydrolytic ring opening, which is responsible for the broadening of the signal of the proton attached to the P-bound carbon atom.

**Scheme 3:**
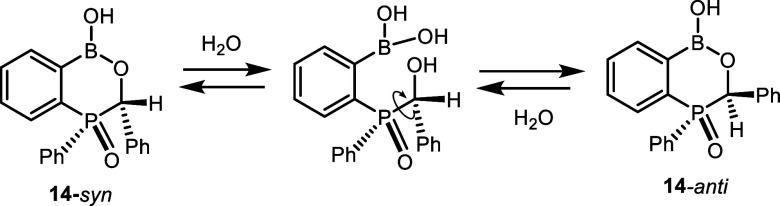
Structure of Two Diastereomers of **14** and Reversible Cleavage of the Heterocyclic Ring in the Less Stable **14**-*syn* Structure

Following the synthesis of **11–14**, the reactions of **6–8** with formaldehyde, acetone, and cyclohexanone gave rise to halogenated benzoxaphosphaborinines **15–22** (Table 1). Moreover, the scope of this protocol was expanded toward the synthesis of thienoxaphosphaborinine derivatives **23–24** from the precursor **10**.

The acidity of selected derivatives was assessed using potentiometric titration with aq. NaOH in the mixed solvent (H_2_O/MeOH, 1:1). The p*K*_a_ value for **11** is 7.2, i.e., it is lower than that reported for the parent benzoxaborinine (p*K*_a_ = 8.4),^33,34^ which indicates that the replacement of the methylene fragment with the P­(O)­Ph moiety in the boracycle leads to a significant acidity enhancement. The p*K*_a_ values for halogenated analogues **15**, **18**, and **20** are decreased to 6.6, 6.3, and 5.7, respectively, reflecting a distinctive acidifying effect of fluorine and chlorine substituents in line with results reported for related oxaboracyclic compounds.^28,34^ The p*K*_a_ value for the thiophene derivative **23** is 6.0, i.e., it is also significantly lower in comparison to **11**.

The crystal structures of **11**, **13**, **20**, **22**, and **23** were confirmed by single-crystal X-ray diffraction (Figure 2). The analysis of molecular structures showed that the geometry of the oxaphosphaborinine heterocycle is similar among all studied compounds including thiophene-based derivative **23**. The average P–C­(sp^2^) distance is equal to 1.79 Å, i.e., it is shorter with respect to the P–C­(sp^3^) one (1.85 Å). The P=O double-bond distances of 1.49 Å are comparable to those found in the crystal structure of diphenylphosphonates Ph_2_P­(O)­OR (1.47–1.49 Å).^35–38^ As observed for benzosiloxa- and benzoxaboroles,^39–43^ endocyclic B–O bonds are slightly longer (av. 1.38 Å) than exocyclic ones (av. 1.34 Å). The six-membered oxaphosphaborinine heterocycle features essentially a half-boat conformation. Thus, it is coplanar with the adjacent aromatic ring, except for the sp^3^-hybridized carbon atom. In all structures, intermolecular OH···O hydrogen bonds connect OH and P=O groups in a head-to-tail fashion giving rise to chains of a varying topology (Figure 4). In **11** and **13**, the neighboring molecules are rotated one to another, resulting in a helical topology of the entire HB chain. In the case of fluorinated derivatives **20** and **22**, the molecules are related solely by translation. As a result, the respective HB chains have a homochiral character. Unusually, compound **23** crystallizes in the chiral space group *P*4_1_ with as many as 8 molecules in the asymmetric part of the unit cell (Figure S1, Supporting Information) and with two CHCl_3_ molecules. Four molecules have the *R* configuration, and the remaining four have the *S* configuration. The molecules differ mainly in the conformation of the phenyl substituent at the phosphorus atom. The supramolecular structure of **23** consists three different homochiral chains: two of them is composed of alternately connected molecules of the *R* configuration, while the other one is composed of *S* enantiomers (Figure S2, Supporting Information).

**Figure 4:**
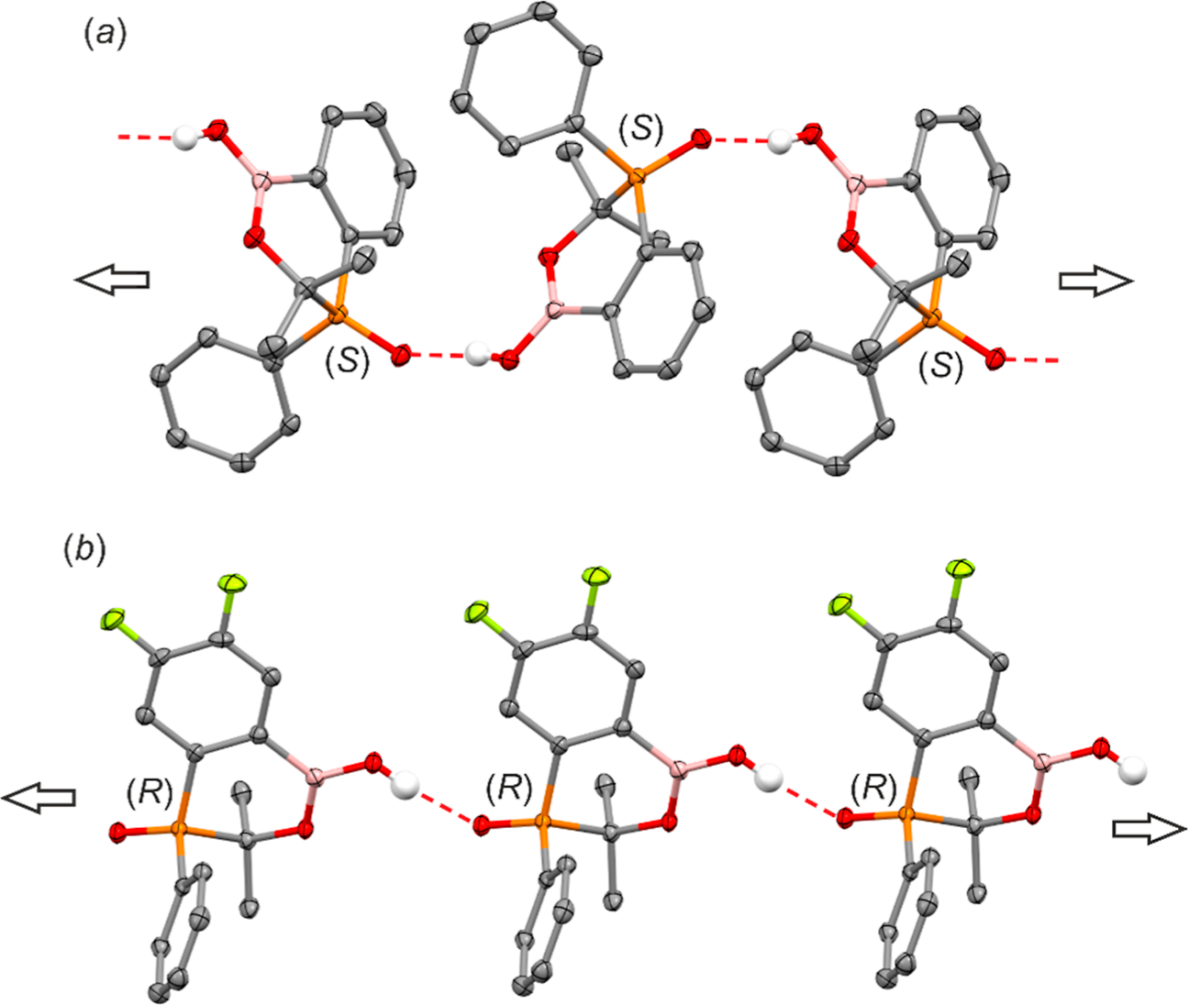
Hydrogen-bonded chains in (a) **11** and (b) **20**. Thermal ellipsoids were generated at the 50% probability level. Carbon-bonded hydrogen atoms were omitted for clarity. Both compounds crystallize in centrosymmetric space groups (*P*1̅ structure **11**; *P*2_1_/*c* structure **20**) containing chains of opposite configurations.

## Conclusions

In conclusion, we found that (2-(phenylhydrophosphoryl)­aryl)­boronic acids **5–8** and **10** were obtained from respective 2-(2′-aryl)-6-butyl[1.3.6.2]­dioxazaborocans **1–4** and **9** through a two-step lithiation/phosphorylation protocol. The nucleophilic character of the phosphorus atom in those compounds was manifested by facile cyclocondensation reactions with carbonyl electrophiles, resulting in the formation of respective benzoxa- and thienoxaphosphaborinines. The structures of the selected products were confirmed by single-crystal X-ray diffraction. Moreover, DFT calculations shed light on the reaction mechanism and thus provided evidence for a significant activating effect of the ortho-boronic group on the addition of the secondary phosphine oxide to the electrophilic carbonyl group. Overall, the results extend the chemistry of arylboronic acids and indicate, once again, that their appropriate functionalization can give rise to novel boracyclic derivatives.

## Experimental Section

### General Comments

Solvents used for reactions were dried by heating to reflux (oil bath) with sodium/benzophenone and distilled under argon. Starting materials and other reagents including halogenated benzenes, alkyllithiums, diisopropylamine, trimethyl borate, and dichlorophenylphosphine were used as received without further purification. Reactions involving organometallic compounds were carried out under an argon atmosphere. Detailed procedures for the synthesis of starting materials **1**,^16^ **2–3**,^27^ **4**,^28^ and **9** ^29^ are described in our previous works. ^1^H, ^13^C, ^19^F, and ^31^P NMR spectra were recorded on an Agilent NMR 400 MHz DDR2 spectrometer. ^11^B NMR spectra were recorded on a Bruker AVANCE III 300 MHz spectrometer. In the ^13^C NMR spectra, the resonances of boron-bonded carbon atoms were not observed in most cases as a result of their broadening by a quadrupolar boron nucleus. ^1^H and ^13^C NMR chemical shifts are given relative to those of TMS using residual solvent resonances. ^11^B and ^19^F NMR chemical shifts are given relative to those of BF_3_·Et_2_O and CFCl_3_, respectively. ^31^P NMR chemical shifts are given relative to 85% phosphoric acid solution in D_2_O. High-resolution mass spectroscopy (HRMS) spectra were recorded using a Synapt G2-S HDMS mass spectrometer (Waters) equipped with an electrospray ionization (ESI) ion source and a quadrupole time-of-flight (QToF) mass analyzer. An Acquity UPLC I-Class system (Waters) was used for sample transfer to the mass spectrometer. The mobile-phase flow rate was set to 100 μL/min, and the sample was dissolved and injected (1 μL) directly into the ESI source. Fragmentation spectra were obtained using a tandem mass spectrometer 6460 (Agilent Technologies) equipped with an electrospray ionization (ESI) ion source and a triple quadrupole (QqQ) mass analyzer. A 1200 HPLC system (Agilent Technologies) was used for sample transfer to the mass spectrometer. The mobile-phase flow rate was set to 200 μL/min, and the sample was dissolved and injected (20 μL, *c* ≈ 50 μg/mL) directly into the Supporting Information source. Spectra were obtained in scan and product ion modes.

### (2-(Phenylhydrophosphoryl)­phenyl)­boronic Acid (**5**)

A solution of **1** (5.0 g, 15.4 mmol) in THF (20 mL) was added dropwise to a stirred solution of *n*-BuLi (1.6 M solution in hexane, 12.0 mL, 19.2 mmol) in THF (40 mL) at −78 °C. The precipitation of a white solid was observed. After 1 h of stirring, the resulting suspension was cooled to −100 °C and PhPCl_2_ (2.9 mL, 20.8 mmol) was added rapidly, in one portion. An immediate increase of temperature from −100 to −70 °C and gradual dissolution of the precipitate were observed. The reaction mixture was again cooled to −100 °C and stirred at this temperature for 1 h. Then, it was slowly warmed up to ca. – 40 °C, quenched with 2 M aq. HCl to pH = 2, and stirred for 2 h at room temperature. The one-phase reaction mixture was concentrated under reduced pressure in order to remove THF and other volatile organic components. The resultant aqueous suspension containing a white viscous solid was diluted with Et_2_O (30 mL) to give a clear two-phase mixture. The organic phase was separated, and the aqueous phase was extracted with Et_2_O (3 × 20 mL). The combined organic solutions were dried over anhydrous Na_2_SO_4_ and concentrated to give a white fluffy solid residue. It was dissolved in the mixture of DCM and hexane (60 mL, 1:1 v/v) stirred for 10 min and filtered. The solvents were evaporated to give a white solid which was washed with hexane and filtered. It was mixed with water (10 mL) and stirred for 30 min, which resulted in the formation of a viscous material. The aqueous phase was decanted. The residue was washed with water and dissolved in DCM. The solution was dried with Na_2_SO_4_ and evaporated to give compound **5** as a white powder. Yield 2.34 g (30%). ^1^H NMR (300 MHz, CDCl_3_): δ 8.96 (s), 8.31–8.19 (m), 7.77–7.42 (m), 6.81–6.44 (m) ppm. ^11^B NMR δ 28.6, 6.6 ppm. ^13^C­{^1^H} NMR (101 MHz, CDCl_3_): δ 138.5 (d, *J* = 13.1 Hz), 132.9 (d, *J* = 2.7 Hz), 132.4 (d, *J* = 3.2 Hz), 131.5, 130.9 (d, *J* = 11.2 Hz), 130.7, 130.3 (d, *J* = 14.8 Hz), 129.1 (d, *J* = 13.0 Hz), 128.6 (d, *J* = 14.1 Hz) ppm. ^31^P­{^1^H} NMR (122 MHz, CDCl_3_): δ 34.1, 21.5 (impurity: PhP­(O)­(H)­OH) ppm. ^31^P NMR (122 MHz, DMSO-*d*_6_): δ 109.2, 30.9 (dq, *J* = 506.7, 14.1 Hz), 21.9 (impurity PhP­(O)­(H)­OH): dt, *J* = 545.2, 14.5 Hz) ppm. HRMS (ESI, positive ion mode) *m*/*z*: [M – H_2_ + H]^+^ Calcd for C_12_H_11_BO_3_P, 245.0533; Found, 245.0533, [M – H_2_O + H]^+^ Calcd for C_12_H_11_BO_2_P, 229.0584; Found, 229.0585.

### (4-Fluoro-2-(phenylhydrophosphoryl)­phenyl)­boronic Acid (**6**)

The synthesis was performed as described for **5** starting with **2** (3.78 g, 11.0 mmol). The product **6** was obtained as a white powder after washing with a mixture of DCM and cyclohexane (20 mL, 1:4 v/v). Yield 1.43 g (49%). ^1^H NMR (300 MHz, DMSO-*d*_6_): δ 9.20 (s, 1H), 8.24–6.91 (m, 8H), 7.49 (d, *J* = 546.7 Hz, 1H) ppm. ^11^B NMR (96 MHz, CDCl_3_): δ 29.6, 5.7 ppm.^13^C NMR (151 MHz, DMSO-*d*_6_): δ 162.4 (d, *J* = 237.5 Hz), 132.2, 131.5, 130.1 (d, *J* = 11.8 Hz), 128.9 (d, *J* = 13.1 Hz), 128.6 (d, *J* = 13.5 Hz), 128.0, 117.9, 114.3 (d, *J* = 19.6 Hz), 113.7 (d, *J* = 19.6 Hz), 112.2 ppm. ^19^F NMR (282 MHz, CDCl_3_): δ −108.2 ppm. ^31^P­{^1^H} NMR (122 MHz, CDCl_3_): δ 102.4, 23.2 (d, *J* = 24.4 Hz), 16.4 (d, *J* = 34.6 Hz) ppm. ^31^P NMR (122 MHz, DMSO-*d*_6_): δ 103.1, 23.8 (dd, *J* = 517.4, 12.2 Hz), 17.1 (d, *J* = 547.7 Hz) ppm. HRMS (ESI, positive ion mode) *m*/*z*: [M + H]^+^ Calcd for C_12_H_12_BFO_3_P, 265.0596; Found, 265.0592.

### (4-Chloro-2-(phenylhydrophosphoryl)­phenyl)­boronic Acid (**7**)

The synthesis was performed as described for **5** starting with **3** (2.84 g, 7.9 mmol). The product **7** was obtained as a white powder after washing with a mixture of DCM and cyclohexane (20 mL, 1:4 v/v). Yield 0.76 g (34%). ^1^H NMR (300 MHz, DMSO-*d*_6_): δ 9.20 (s, 1H), 8.11–7.19 (m, 8H), 7.49 (d, *J* = 547.0 Hz, 1H) ppm. ^11^B NMR (96 MHz, DMSO-*d*_6_): δ 29.8, 5.0 ppm. ^13^C NMR (151 MHz, DMSO-*d*_6_): δ 135.9, 132.2 (d, *J* = 2.9 Hz), 131.1, 130.5, 130.1 (d, *J* = 11.8 Hz), 128.9 (d, *J* = 12.9 Hz), 128.6 (d, *J* = 13.3 Hz), 128.2, 127.4 ppm. ^31^P­{^1^H} NMR (122 MHz, CDCl_3_): δ 102.2, 22.8, 16.5 ppm. ^31^P NMR (122 MHz, DMSO-*d*_6_): δ 102.9, 23.4 (dd, *J* = 517.3, 15.6 Hz), 17.1 (d, *J* = 546.8 Hz ppm). HRMS (ESI, positive ion mode) *m*/*z*: [M + H]^+^ Calcd for C_12_H_12_BClO_3_P, 281.0300; Found, 281.0297.

### (4,5-Difluoro-2-(phenylhydrophosphoryl)­phenyl)­boronic Acid (**8**)

The synthesis was performed as described for **5** starting with **4** (3.62 g, 10.0 mmol). The product **8** was obtained as a white powder after washing with a mixture of DCM and cyclohexane (20 mL, 1:4 v/v). Yield 1.25 g (44%). ^1^H NMR (300 MHz, DMSO-*d*_6_): δ 9.20 (s, 1H), 8.15–7.00 (m, 7H), 7.49 (d, *J* = 547.3 Hz, 1H) ppm. ^11^B NMR (96 MHz, CDCl_3_): δ 28.9, 6.0 ppm. ^13^C NMR (151 MHz, DMSO-*d*_6_): δ 132.4, 131.3, 130.5 (d, *J* = 11.5 Hz), 130.1 (d, *J* = 11.7 Hz), 128.8 (d, *J* = 12.8 Hz), 128.1, 124.7, 122.3 (d, *J* = 14.2 Hz), 120.9 ppm. ^19^F NMR (282 MHz, CDCl_3_): δ −131.2, −133.1 ppm. ^31^P­{^1^H} NMR (122 MHz, DMSO-*d*_6_): δ 103.5, 21.5 (d, *J* = 3.7 Hz), 16.4 ppm. ^31^P NMR (122 MHz, DMSO-*d*_6_): δ 104.2, 22.1 (dd, *J* = 520.7, 14.9 Hz), 17.1 (d, *J* = 547.2 Hz) ppm. HRMS (ESI, positive ion mode) *m*/*z*: [M + H]^+^ Calcd for C_12_H_11_BF_2_O_3_P, 283.0501; Found, 283.0496.

### (2-(Phenylhydrophosphoryl)­thiophen-3-yl)­boronic Acid (**10**)

A solution of **9** (5.06 g, 20.0 mmol) in THF (20 mL) was added dropwise at −78 °C to a stirred solution of LDA, freshly prepared from *n*-BuLi (2.5 M in hexane, 8.8 mL, 22.0 mol) and diisopropylamine (2.30 g, 3.2 mL, 23.0 mmol) in THF (30 mL) at −78 °C. After ca. 30 min of stirring at −100 °C, PhPCl_2_ (14.19 g, 2.98 mL, 0.150 mol) was added rapidly, in one portion. The reaction mixture was then stirred for 30 min at −100 °C. An immediate increase of temperature from −100 to −70 °C was observed. The reaction mixture was stirred at this temperature for 1 h. The further workup was performed as described for **5** to give **10** as a white powder after washing with a mixture of DCM and cyclohexane (20 mL, 1:4 v/v). Yield 2.03 g (41%). ^1^H NMR (300 MHz, DMSO-*d*_6_): δ 9.38 (s, 1H), 8.05–7.34 (m, 7H), 7.49 (d, *J* = 547.0 Hz, 1H) ppm. ^11^B NMR (96 MHz, CDCl_3_): δ 27.1, 2.9 ppm. ^13^C NMR (151 MHz, CDCl_3_): δ 147.7, 137.7 (d, *J* = 15.7 Hz), 136.3 (d, *J* = 110.9 Hz), 133.5, 132.9, 132.5, 131.9 (d, *J* = 11.2 Hz), 131.2, 130.9 (d, *J* = 11.9 Hz), 130.6 (d, *J* = 11.6 Hz), 129.2 (d, *J* = 13.3 Hz), 128.7 (d, *J* = 14.1 Hz), 127.0, 125.2 ppm. ^31^P­{^1^H} NMR (122 MHz, CDCl_3_): δ 17.4 ppm. ^31^P NMR (122 MHz, CDCl_3_): δ 18.1 (dt, *J* = 508.4, 14.7 Hz) ppm. HRMS (ESI, positive ion mode) *m*/*z*: [M + H]^+^ Calcd for C_10_H_11_BO_3_PS, 253.0254; Found: 253.0252, [M – H_2_ + H]^+^ Calcd for C_10_H_9_BO_3_PS, 251.0098; Found, 251.0096.

### 1-Hydroxy-3,3-dimethyl-4-phenyl-1,3-dihydrobenzo­[*c*]­[1,5,2]­oxaphosphaborinine 4-Oxide (**11**)

Compound **5** (150 mg, 0.6 mmol) was dissolved in acetone (2 mL) under an argon atmosphere. The resultant solution was stirred for 24 h at room temperature. After that time, the precipitation of a white solid was observed. It was filtered under reduced pressure, washed several times with hexane, and finally dried in vacuo (∼10^–3^ mbar) to give **11** as a white powder, mp 200–203 °C. Yield 90 mg (52%). ^1^H NMR (300 MHz, CDCl_3_): δ 8.10–8.02 (m, 1H), 8.02–7.92 (m, 1H), 7.69–7.62 (m, 4H), 7.56–7.47 (m, 1H), 7.47–7.37 (m, 2H), 5.63 (s, 1H), 1.62 (d, *J* = 12.0 Hz, 3H), 1.34 (d, *J* = 12.9 Hz, 3H) ppm. ^11^B NMR (96 MHz, CDCl_3_): δ 26.8 (broad) ppm. ^13^C­{^1^H} NMR (101 MHz, CDCl_3_): δ 134.24 (d, *J* = 93.1 Hz), 134.17 (d, *J* = 11.0 Hz), 132.4 (d, *J* = 11 Hz), 132.1 (d, *J* = 9 Hz), 131.9 (d, *J* = 3 Hz), 131.8 (d, *J* = 3 Hz), 130.8 (d, *J* = 7 Hz), 129.5 (d, *J* = 94 Hz), 128.4 (*J* = 11 Hz), 73.8 (d, *J* = 79 Hz), 25.5 (d, *J* = 6 Hz), 24.1 (d, *J* = 4 Hz) ppm. ^31^P­{^1^H} NMR (122 MHz, CDCl_3_): δ = 28.7 ppm. HRMS (ESI, positive ion mode) *m*/*z*: [M + H]^+^ Calcd for C_15_H_17_BO_3_P, 287.1003; Found, 287.1002.

### 1-Hydroxy-4-phenyl-1,3-dihydrobenzo­[*c*]­[1,5,2]­oxaphosphaborinine 4-Oxide (**12**)

Compound **5** (400 mg, 1.6 mmol) was dissolved in CHCl_3_ (6 mL) under an argon atmosphere, followed by the addition of formaldehyde (37% solution in water, 0.4 mL, 5.3 mmol). The reaction mixture was stirred for 3 days at room temperature. After that time, the precipitation of a white solid was observed. The resultant suspension was concentrated under high vacuum (∼10^–3^ mbar) and a sticky solid residue was obtained. It was dissolved in the mixture of CHCl_3_ and hexane (10 mL, 1:1 v/v) and stirred for 1 h. Then, solvents and other volatile components were removed under reduced pressure to give a white solid. It was washed with hexane, filtered, and dried in vacuo (∼10^–3^ mbar), to afford **12** as a white powder, mp 150–155 °C. Yield 210 mg (51%). ^1^H NMR (300 MHz, CDCl_3_): δ 8.05 (q, *J* = 4.3 Hz, 1H), 7.94–7.87 (m, 1H), 7.73–7.62 (m, 4H), 7.60–7.54 (m, 1H), 7.52–7.42 (m, 2H), 4.69 (dd, *J* = 14.5, 8.1 Hz, 1H), 4.57 (dd, *J* = 14.5, 5.0 Hz, 1H) ppm. ^11^B NMR (96 MHz, CDCl_3_): δ 27.4 ppm. ^13^C­{^1^H} NMR (101 MHz, CDCl_3_): δ 135.5 (d, *J* = 98 Hz), 134.8 (d, *J* = 11 Hz), 132.5 (d, *J* = 3 Hz), 132.2 (*J* = 2 Hz), 132.1 (*J* = 11 Hz), 131.3 (*J* = 10 Hz), 129.7 (*J* = 8 Hz), 128.7 (*J* = 12 Hz), 63.6 (d, *J* = 75 Hz) ppm. ^31^P­{^1^H} NMR (122 MHz, CDCl_3_): δ 19.8 ppm. HRMS (ESI, positive ion mode) *m*/*z*: [M + H]^+^ Calcd for C_13_H_13_BO_3_P, 259.0690; Found, 259.0687.

### 1-Hydroxy-4-phenyl-1*H*-spiro­[benzo­[*c*]­[1,5,2]­oxaphosphaborinine-3,1′-cyclohexane] 4-Oxide (**13**)

Compound **5** was (120 mg, 0.5 mmol) dissolved in anhydrous cyclohexanone (3 mL) under an argon atmosphere. The reaction mixture was stirred for 24 h at room temperature. After that time, the precipitation of a yellow solid was observed. The resultant suspension was concentrated under high vacuum (∼10^–3^ mbar) and a sticky solid residue was obtained. It was dissolved in a mixture of Et_2_O and hexane (10 mL, 1:1 v/v) and stirred for 1 h. Then, solvents and other volatile components were removed under reduced pressure (∼10^–3^ mbar) to give a yellow solid. It was washed with hexane, filtered, and dried in vacuo (∼10^–3^ mbar) to afford **13** as a pale-yellow powder, mp 188–192 °C. Yield 63 mg (39%). ^1^H NMR (400 MHz, CDCl_3_): δ 8.00 (tdd, *J* = 4.6, 3.1, 0.7 Hz, 1H), 7.98–7.91 (m, 1H), 7.66–7.56 (m, 4H), 7.51–7.46 (m, 1H), 7.42–7.35 (m, 2H), 2.15–2.05 (m, 1H), 1.87–1.78 (m, 2H), 1.64–1.48 (m, 6H), 1.27 (dt, *J* = 12.8, 7.6 Hz, 1H) ppm. ^11^B NMR (96 MHz, CDCl_3_): δ 27.2 ppm. ^13^C­{^1^H} NMR (101 MHz, CDCl_3_): δ 133.9 (d, *J* = 10.8 Hz), 133.7 (d, *J* = 92.7 Hz), 132.3 (d, *J* = 11.4 Hz), 132.2 (d, *J* = 8.9 Hz), 131.8 (d, *J* = 3.0 Hz), 131.7 (d, *J* = 2.7 Hz), 130.9 (d, *J* = 6.8 Hz), 129.2 (d, *J* = 94.0 Hz), 128.4 (d, *J* = 11.4 Hz), 74.8 (d, *J* = 81.8 Hz), 31.9 (d, *J* = 4.1 Hz), 30.3 (d, *J* = 2.3 Hz), 24.9, 20.1 (d, *J* = 8.3 Hz), 19.7 (d, *J* = 8.9 Hz) ppm. ^31^P­{^1^H} NMR (122 MHz, CDCl_3_): δ = 27.6 ppm. HRMS (ESI, positive ion mode) *m*/*z*: [M + H]^+^ Calcd for C_18_H_21_BO_3_P, 327.1316; Found, 327.1312.

### 1-Hydroxy-3,4-diphenyl-1,3-dihydrobenzo­[*c*]­[1,5,2]­oxaphosphaborinine 4-Oxide (**14**, Mixture of Cis and Trans Diastereoisomers)

Compound **5** (300 mg, 1.2 mmol) was dissolved in CHCl_3_ (5 mL) under an argon atmosphere, followed by the addition of benzaldehyde (0.13 mL, 1.2 mmol). The reaction mixture was stirred for 2 days at ambient temperature. The solvent was removed under reduced pressure and a solid residue was suspended in hexane and stirred overnight. Then, it was filtered, dissolved in CHCl_3_, and stirred for 30 min. Upon the addition of hexane, the precipitation of a white solid was observed. It was finally filtered under reduced pressure and dried in vacuo (∼10^–3^ mbar) to give **14** as a white powder, mp 168–171 °C. Yield 260 mg (64%). ^1^H NMR (300 MHz, CDCl_3_): δ 8.23:7.97 (m, 2H), 7.85:7.51 (m, 4H), 7.48:7.35 (m, 2H), 7.24:7.16 (m, 6H), 7.10:6.96 (m, 1H), 5.89:5.48 (m, 1H), 5.07 (s, 1H) ppm. ^11^B NMR (96 MHz, CDCl_3_): δ 27.6 ppm. ^13^C NMR (151 MHz, DMSO-*d*_6_): δ 136.5 (d, *J* = 94.7 Hz), 135.9 (d, *J* = 93.9 Hz), 134.8 (d, *J* = 3.1 Hz), 134.6 (d, *J* = 3.7 Hz), 134.18 (d, *J* = 11.7 Hz), 134.12 (d, *J* = 10.3 Hz), 132.4, 131.16, 132.10, 132.0, 131.91, 131.88, 131.8 (d, *J* = 9.2 Hz), 131.3 (d, *J* = 8.7 Hz), 130.1, 130.0, 129.8 (d, *J* = 8.5 Hz), 129.6 (d, *J* = 6.9 Hz), 128.6 (d, *J* = 11.8 Hz), 128.1 (d, *J* = 11.2 Hz), 127.71 (d, *J* = 32.8 Hz), 127.68 (d, *J* = 35.5 Hz), 127.4 (d, *J* = 4.3 Hz), 126.3 (d, *J* = 4.0 Hz), 74.8 (d, *J* = 72.6 Hz). 74.7 (d, *J* = 72.9 Hz) ppm. ^31^P­{^1^H} (122 MHz, DMSO-*d*_6_): δ 20.9, 19.5.ppm. HRMS (ESI, positive ion mode) *m*/*z*: [M + H]^+^ Calcd for C_19_H_17_BO_3_P, 335.1003; Found, 335.0998.

### 6-Fluoro-1-hydroxy-3,3-dimethyl-4-phenyl-1,3-dihydrobenzo­[*c*]­[1,5,2]­oxaphosphaborinine 4-Oxide (**15**)

The synthesis was performed as described for **11** starting with **6** (264 mg, 1.0 mmol) and acetone (2.0 mL). The product **15** was obtained as a white powder, mp 196–199 °C. Yield 78 mg (26%). ^1^H NMR (400 MHz, CDCl_3_): δ 8.07 (dt, *J* = 8.2, 5.1 Hz, 1H), 7.69–7.58 (m, 3H), 7.55–7.47 (m, 1H), 7.40 (td, *J* = 7.8, 2.7 Hz, 2H), 7.29 (td, *J* = 8.7, 2.6 Hz, 1H), 1.58 (d, *J* = 12.2 Hz, 3H), 1.29 (d, *J* = 13.2 Hz, 3H) ppm. ^11^B NMR (96 MHz, CDCl_3_): δ 26.5 (broad) ppm. ^13^C­{^1^H} NMR (101 MHz, DMSO-*d*_6_): δ 165.0 (dd, *J* = 253.2, 14.9 Hz), 138.9–137.8 (m), 137.8 (dd, *J* = 12.4, 8.0 Hz), 132.8 (d, *J* = 2.7 Hz), 132.0 (d, *J* = 8.7 Hz), 130.8, 129.7 (d, *J* = 93.3 Hz), 129.2 (d, *J* = 11.2 Hz), 119.7 (dd, *J* = 20.2, 2.4 Hz), 117.2 (dd, *J* = 21.3, 7.7 Hz), 73.3 (d, *J* = 80.0 Hz), 25.6 (d, *J* = 6.1 Hz), 24.3 (d, *J* = 4.6 Hz) ppm. ^19^F NMR (376 MHz, CDCl_3_): δ −104.39 (tt, *J* = 8.5, 5.0 Hz) ppm. ^31^P­{^1^H} NMR (162 MHz, CDCl_3_): δ 28.5 (d, *J* = 4.5 Hz) ppm. HRMS (ESI, positive ion mode) *m*/*z*: [M + H]^+^ Calcd for C_15_H_16_BFO_3_P, 305.0909; Found, 305.0908.

### 6-Fluoro-1-hydroxy-4-phenyl-1,3-dihydrobenzo­[*c*]­[1,5,2]­oxaphosphaborinine 4-Oxide (**16**)

The synthesis was performed as described for **12** starting with **6** (264 mg, 1.0 mmol) and formaldehyde (37% solution in water, 0.5 mL, 6.6 mmol). The product **16** was obtained as a white powder, mp 180–185 °C. Yield 181 mg (66%). ^1^H NMR (400 MHz, CDCl_3_): δ 8.09–8.01 (m, 1H), 7.64 (dd, *J* = 11.7, 7.8 Hz, 2H), 7.55 (dd, *J* = 9.1, 8.2 Hz, 1H), 7.47 (bd, *J* = 6.9 Hz, 2H), 7.35–7.27 (m, 2H), 4.66 (dd, *J* = 14.3, 8.7 Hz, 1H), 4.55 (bdd, *J* = 14.4, 4.8 Hz, 1H) ppm. ^11^B NMR (96 MHz, CDCl_3_): δ 28.0 ppm. ^13^C­{^1^H} NMR (101 MHz, CDCl_3_): δ 165.0 (d, *J* = 255.3 Hz), 138.0, 132.7, 132.1, 131.2 (d, *J* = 9.5 Hz), 128.9 (d, *J* = 12.1 Hz), 128.3, 120.6–118.8 (m), 116.7 (d, *J* = 22.2 Hz), 63.3 (d, *J* = 76.5 Hz) ppm. ^19^F NMR (376 MHz, CDCl_3_): δ −104.23 ppm. ^31^P­{^1^H} NMR (162 MHz, CDCl_3_): δ 18.7 ppm. HRMS (ESI, positive ion mode) *m*/*z*: [M + H]^+^ Calcd for C_13_H_12_BFO_3_P, 277.0596; Found, 277.0596.

### 6-Fluoro-1-hydroxy-4-phenyl-1*H*-spiro­[benzo­[*c*]­[1,5,2]­oxaphosphaborinine-3,1′-cyclohexane] 4-Oxide (**17**)

The synthesis was performed as described for **13** starting with **6** (264 mg, 1.0 mmol) and anhydrous cyclohexanone (3.0 mL). The product **17** was obtained as a pale-yellow powder, mp 165–170 °C. Yield 219 mg (64%). ^1^H NMR (400 MHz, CDCl_3_): δ 8.03 (dt, *J* = 8.3, 5.2 Hz, 1H), 7.69–7.63 (m, 1H), 7.62–7.54 (m, 2H), 7.53–7.46 (m, 1H), 7.41 (ddd, *J* = 8.4, 6.8, 2.9 Hz, 2H), 7.35–7.26 (m, 1H), 2.23–1.99 (bm, 1H), 1.95–1.72 (bm, 2H), 1.72–1.49 (bm, 6H), 1.42 (br s, 1H), 1.34–1.21 (m, 1H) ppm. ^11^B NMR (96 MHz, CDCl_3_): δ 28.1 ppm. ^13^C­{^1^H} NMR (101 MHz, CDCl_3_): δ 160.2 (dd, *J* = 256.0, 14.9 Hz), 131.8 (dd, *J* = 90.6, 5.9 Hz), 131.8 (dd, *J* = 12.4, 7.6 Hz), 126.9 (d, *J* = 8.0 Hz), 126.9 (d, *J* = 4.0 Hz), 125.7 (broad), 123.3 (d, *J* = 11.5 Hz), 123.2 (d, *J* = 95.2 Hz), 114.0 (dd, *J* = 20.3, 2.5 Hz), 112.8 (dd, *J* = 21.6, 7.3 Hz), 69.8 (d, *J* = 82.3 Hz), 26.7, 25.1, 19.6, 14.9 (d, *J* = 8.5 Hz), 14.6 (d, *J* = 8.8 Hz) ppm. ^19^F NMR (376 MHz, CDCl_3_): δ −104.15 (tt, *J* = 8.9, 4.7 Hz) ppm. ^31^P­{^1^H} NMR (162 MHz, CDCl_3_): δ 27.0 (d, *J* = 4.7 Hz) ppm. HRMS (ESI, positive ion mode) *m*/*z*: [M + H]^+^ Calcd for C_18_H_20_BFO_3_P 345.1222; Found, 345.1221.

### 6-Chloro-1-hydroxy-3,3-dimethyl-4-phenyl-1,3-dihydrobenzo­[*c*]­[1,5,2]­oxaphosphaborinine 4-Oxide (**18**)

The synthesis was performed as described for **11** starting with **7** (280 mg, 1.0 mmol) and acetone (3.0 mL). The product **18** was obtained as a white powder, mp 196–200 °C. Yield 196 mg (61%). ^1^H NMR (400 MHz, DMSO-*d*_6_): δ 7.94 (dd, *J* = 8.0, 4.4 Hz, 1H), 7.76 (ddd, *J* = 8.1, 2.1, 0.9 Hz, 1H), 7.69 (dd, *J* = 11.3, 2.1 Hz, 1H), 7.61–7.57 (m, 1H), 7.55–7.49 (m, 4H), 1.44 (d, *J* = 12.2 Hz, 3H), 1.24 (d, *J* = 13.1 Hz, 3H) ppm. ^11^B NMR (96 MHz, CDCl_3_): δ 26.6 (broad) ppm. ^13^C­{^1^H} NMR (101 MHz, DMSO-*d*_6_): δ 137.6 (d, *J* = 74.5 Hz), 136.6 (d, *J* = 11.3 Hz), 132.8 (d, *J* = 2.8 Hz), 132.5 (d, *J* = 2.3 Hz), 132.0 (d, *J* = 8.7 Hz), 129.9 (d, *J* = 7.8 Hz), 129.6 (d, *J* = 93.3 Hz), 129.3 (d, *J* = 11.2 Hz), 128.2 (d, *J* = 12.3 Hz), 73.3 (d, *J* = 79.8 Hz), 25.6 (d, *J* = 6.1 Hz), 24.3 (d, *J* = 4.7 Hz) ppm. ^31^P­{^1^H} NMR (162 MHz, DMSO-*d*_6_): δ 25.7 ppm. HRMS (ESI, positive ion mode) *m*/*z*: [M + H]^+^ Calcd for C_15_H_16_BClO_3_P, 321.0613; Found, 321.0609.

### 6-Chloro-1-hydroxy-4-phenyl-1*H*-spiro­[benzo­[*c*]­[1,5,2]­oxaphosphaborinine-3,1′-cyclohexane] 4-Oxide (**19**)

The synthesis was performed as described for **13** starting with **7** (200 mg, 0.7 mmol) and anhydrous cyclohexanone (2.5 mL). The product **19** was obtained as a pale-yellow powder, mp 187–191 °C. Yield 103 mg (41%). ^1^H NMR (400 MHz, CDCl_3_): δ 7.97:7.88 (m, 1H), 7.83:7.09 (bm, 7H), 2.16:1.74 (m, 2H), 1.58 (m, 5H), 1.52:1.33 (m, 1H), 1.26 (s, 2H) ppm. ^11^B NMR (96 MHz, CDCl_3_): δ 28.0 ppm. ^13^C­{^1^H} NMR (101 MHz, CDCl_3_): δ 139.5 (d, *J* = 13.9 Hz), 135.6 (d, *J* = 11.5 Hz), 132.2, 132.1 (d, *J* = 9.1 Hz), 131.0 (d, *J* = 7.5 Hz), 128.6 (d, *J* = 11.7 Hz), 128.3 (d, *J* = 86.6 Hz), 75.4 (d, *J* = 82.0 Hz), 31.9, 30.3, 24.7, 20.1 (d, *J* = 8.5 Hz), 19.8 (d, *J* = 9.2 Hz) ppm. ^31^P­{^1^H} NMR (162 MHz, CDCl_3_): δ 27.0 ppm. HRMS (ESI, positive ion mode) *m*/*z*: [M + H]^+^ Calcd for C_18_H_20_BClO_3_P, 361.0926; Found, 361.0920.

### 6,7-Difluoro-1-hydroxy-3,3-dimethyl-4-phenyl-1,3-dihydrobenzo­[*c*]­[1,5,2]­oxaphosphaborinine 4-Oxide (**20**)

The synthesis was performed as described for **11** starting with **8** (282 mg, 1.0 mmol) and acetone (2.5 mL). The product **20** was obtained as a white powder, mp 194–198 °C. Yield 133 mg (41%). ^1^H NMR (400 MHz, DMSO-*d*_6_): δ 9.41 (s, 1H), 7.87 (ddd, *J* = 10.7, 8.0, 3.8 Hz, 1H), 7.75 (ddd, *J* = 11.3, 9.8, 7.6 Hz, 1H), 7.60 (tdd, *J* = 5.9, 3.1, 1.4 Hz, 1H), 7.56–7.48 (m, 4H), 1.45 (d, *J* = 12.2 Hz, 3H), 1.23 (d, *J* = 13.2 Hz, 3H) ppm. ^11^B NMR (96 MHz, CDCl_3_): δ 26.1 (broad) ppm. ^13^C­{^1^H} NMR (101 MHz, DMSO-*d*_6_): δ 152.7 (dd, *J* = 256.0, 31.9 Hz), 152.6 (ddd, *J* = 253.7, 29.4, 2.7 Hz), 133.7 (t, *J* = 4.3 Hz), 132.9 (d, *J* = 2.6 Hz), 132.0 (d, *J* = 8.7 Hz), 130.1 (dd, *J* = 123.8, 12.6 Hz), 129.3 (d, *J* = 11.2 Hz), 130.4–128.6 (m), 123.6 (dd, *J* = 15.4, 12.7 Hz), 120.4 (dd, *J* = 17.1, 8.7 Hz), 73.4 (d, *J* = 80.3 Hz), 25.6 (d, *J* = 6.1 Hz), 24.3 (d, *J* = 4.8 Hz) ppm. ^19^F NMR (376 MHz, CDCl_3_): δ −129.71 (dtd, *J* = 20.4, 8.5, 3.3 Hz), −131.70 (ddd, *J* = 20.6, 10.4, 7.6 Hz) ppm. ^31^P­{^1^H} NMR (162 MHz, CDCl_3_): δ 28.0 ppm. HRMS (ESI, positive ion mode) *m*/*z*: [M + H]^+^ Calcd for C_15_H_15_BF_2_O_3_P, 323.0814; Found, 323.0815.

### 6,7-Difluoro-1-hydroxy-4-phenyl-1,3-dihydrobenzo­[*c*]­[1,5,2]­oxaphosphaborinine 4-Oxide (**21**)

The synthesis was performed as described for **12** starting with **8** (282 mg, 1.0 mmol) and formaldehyde (37% solution in water, 0.5 mL, 6.6 mmoL). The product **21** was obtained as a white powder, mp 172–176 °C. Yield 236 mg (80%). ^1^H NMR (400 MHz, CDCl_3_): δ 7.85 (bd, *J* = 11.2 Hz, 1H), 7.71–7.51 (m, 4H), 7.47 (td, *J* = 7.7, 3.0 Hz, 2H), 4.64 (dd, *J* = 14.6, 8.6 Hz, 1H), 4.53 (dd, *J* = 14.7, 3.7 Hz, 1H) ppm. ^11^B NMR δ 26.2 ppm. ^13^C­{^1^H} NMR (101 MHz, CDCl_3_): δ 132.9 (d, *J* = 2.7 Hz), 131.1 (d, *J* = 10.0 Hz), 129.0 (d, *J* = 12.2 Hz), 119.8–119.3 (m), 63.8 (d, *J* = 77.0 Hz) ppm. ^19^F NMR (376 MHz, CDCl_3_): δ −129.74, −130.52 to −131.23 (m) ppm. ^31^P­{^1^H} NMR (162 MHz, CDCl_3_): δ 18.6 ppm. HRMS (ESI, positive ion mode) *m*/*z*: [M + H]^+^ Calcd for C_13_H_11_BF_2_O_3_P, 295.0501; Found, 295.0499.

### 6,7-Difluoro-1-hydroxy-4-phenyl-1*H*-spiro­[benzo­[*c*]­[1,5,2]­oxaphosphaborinine-3,1′-cyclohexane] 4-Oxide (**22**)

The synthesis was performed as described for **13** starting with **8** (282 mg, 1.0 mmol) and anhydrous cyclohexanone (3.0 mL). The product **22** was obtained as a pale-yellow powder, mp 185–190 °C. Yield 155 mg (43%). ^1^H NMR (400 MHz, CDCl_3_): δ 7.89–7.64 (m, 2H), 7.55 (dt, *J* = 16.4, 8.2 Hz, 3H), 7.44 (d, *J* = 9.0 Hz, 2H), 1.80 (s, 1H), 1.58 (d, *J* = 26.1 Hz, 5H), 1.42 (s, 1H), 1.23 (d, *J* = 21.6 Hz, 2H), 1.04–0.76 (m, 1H) ppm. ^11^B NMR (96 MHz, CDCl_3_): δ 26.9 ppm. ^13^C­{^1^H} NMR (101 MHz, CDCl_3_): δ 132.3 (d, *J* = 2.6 Hz), 132.0 (d, *J* = 9.0 Hz), 128.6 (d, *J* = 11.6 Hz), 123.5 (t, *J* = 14.2 Hz), 120.7 (dd, *J* = 17.1, 9.0 Hz), 75.4 (d, *J* = 82.4 Hz), 31.9, 30.3 (d, *J* = 3.2 Hz), 24.7, 20.0 (d, *J* = 8.5 Hz), 19.8 (d, *J* = 9.2 Hz) ppm. ^19^F NMR (376 MHz, CDCl_3_): δ −127.81 to −130.92 (m), −131.20 to −133.94 (m) ppm. ^31^P­{^1^H} NMR (162 MHz, CDCl_3_): δ 26.6 ppm. HRMS (ESI, positive ion mode) *m*/*z*: [M + H]^+^ Calcd for C_18_H_19_BF_2_O_3_P, 363.1127; Found, 363.1127.

### 1-Hydroxy-3,3-dimethyl-4-phenyl-1,3-dihydrothieno­[3,2-*c*]­[1,5,2]­oxaphosphaborinine 4-Oxide (**23**)

The synthesis was performed as described for **11** starting with **10** (441 mg, 1.8 mmol) and acetone (5.3 mL). The product **23** was obtained as a white powder, mp 186–188 °C. Yield 141 mg (48%). ^1^H NMR (400 MHz, CDCl_3_): δ 7.77 (dd, *J* = 4.7, 3.8 Hz, 1H), 7.67–7.58 (m, 3H), 7.58–7.48 (m, 1H), 7.48–7.38 (m, 2H), 1.67 (d, *J* = 12.3 Hz, 3H), 1.28 (d, *J* = 14.0 Hz, 3H) ppm. ^11^B NMR (96 MHz, CDCl_3_): δ 25.6 (broad) ppm. ^13^C­{^1^H} NMR (101 MHz, DMSO-*d*_6_): δ 144.3, 139.5 (d, *J* = 93.2 Hz), 135.1 (d, *J* = 5.5 Hz), 133.1 (d, *J* = 11.6 Hz), 132.9 (d, *J* = 2.7 Hz), 131.8 (d, *J* = 9.2 Hz), 130.0 (d, *J* = 98.7 Hz), 129.2 (d, *J* = 11.6 Hz), 75.1 (d, *J* = 80.5 Hz), 25.7 (d, *J* = 5.6 Hz), 24.8 (d, *J* = 6.4 Hz) ppm. ^31^P­{^1^H} NMR (162 MHz, DMSO-*d*_6_): δ 25.7 ppm. HRMS (ESI, positive ion mode) *m*/*z*: [M + H]^+^ Calcd for C_13_H_15_BO_3_PS, 293.0567; Found, 293.0563.

### 1′-Hydroxy-4′-phenyl-1′H-spiro­[cyclohexane-1,3′-thieno­[3,2-*c*]­[1,5,2]­oxaphosphaborinine] 4′-Oxide (**24**)

The synthesis was performed as described for **13** starting with **10** (230 mg, 0.9 mmol) and anhydrous cyclohexanone (3.0 mL). The product **24** was obtained as a pale-yellow powder, mp 208–212 °C. Yield 172 mg (58%). ^1^H NMR (400 MHz, CDCl_3_): δ 7.77 (dd, *J* = 4.6, 3.8 Hz, 1H), 7.63–7.55 (m, 3H), 7.52 (td, *J* = 7.3, 1.5 Hz, 1H), 7.46–7.39 (m, 2H), 2.15–1.92 (m, 1H), 1.79–1.56 (m, 6H), 1.51 (d, *J* = 11.7 Hz, 1H), 1.44–1.17 (m, 3H) ppm. ^11^B NMR (96 MHz, CDCl_3_): δ 25.7 ppm. ^13^C­{^1^H} NMR (101 MHz, CDCl_3_): δ 133.8 (d, *J* = 5.9 Hz), 132.3 (d, *J* = 11.4 Hz), 132.2 (d, *J* = 2.9 Hz), 132.0 (d, *J* = 9.4 Hz), 128.7 (d, *J* = 101.0 Hz), 128.5 (d, *J* = 11.9 Hz), 77.2 (d, *J* = 82.5 Hz), 31.8 (d, *J* = 3.7 Hz), 30.8 (d, *J* = 3.9 Hz), 24.8, 20.2 (d, *J* = 8.5 Hz), 20.1 (d, *J* = 9.7 Hz) ppm. ^31^P­{^1^H} NMR (162 MHz, CDCl_3_): δ 27.5 ppm. HRMS (ESI, positive ion mode) *m*/*z*: [M + H]^+^ Calcd for C_16_H_19_BO_3_PS, 333.0880; Found, 333.0875.

### Crystal Structure Determination

Single crystals suitable for X-ray diffraction measurements were obtained by the slow evaporation of DCM solutions. The crystallizations were performed in open vials at room temperature. X-ray diffraction data were collected on a SuperNova diffractometer (*T* = 100 K) equipped with an Atlas detector using Cu–Kα radiation (λ = 1.54184 Å). Data reduction and analysis were carried out with the CrysAlisPro program.^44^ The structures were solved via direct methods using SHELXS-97 ^45^ and refined using SHELXL-2014.^46^ All non-hydrogen atoms were refined anisotropically. All C–H hydrogen atoms were placed in calculated positions with C–H distances of 0.95 Å and *U*_iso_(H) = 1.2*U*_eq_(C). The positions of H atoms of hydroxy groups were located from difference electron density maps. The O–H distances were fixed to 0.84 Å with a standard deviation of 0.01 Å, and the directionality of the O–H was refined freely. The *U*_iso_(H) parameter was set to 1.5*U*_eq_ with respect to oxygen atoms. The diffraction of the crystal **22** was moderate. No diffraction peaks were observed at θ > 65°. In the case of structure **23**, the large positive residue density peaks (up to 2.2 e·Å^–3^) appear in the vicinity of the chloroform molecule indicating disorder of this molecule. The structure of **23** is of moderate quality, but it was difficult to obtain better quality single crystals. In addition, **23** crystallizes as a merohedral twin and contains some voids filled with highly disordered solvent molecules (CHCl_3_). These result in the appearance of several checkCIF B alerts. The response to checkCIF B alerts for structures **22** and **23** is provided in corresponding CIF files. Selected crystal data are summarized in Table S1. The CIF files can be retrieved from the Cambridge Structural Database^47^ (deposition numbers: 2416543–2416547) or the Supporting Information.

### Theoretical Calculations

Theoretical calculations were performed at the M06-2X^22^/6-311++G­(d,p)^23^ level of theory using the Gaussian16 program.^48^ Molecular geometries were taken from the crystal structures, or they were modified using GaussView software. Following geometry optimization, the vibrational frequencies were calculated, and the results showed that optimized structures are stable geometric structures (no imaginary frequencies). To optimize the structures of transition states, a synchronous-transit-guided quasi-Newton approach (QST3) was applied. In this method, three input structures are needed: one corresponds to reactants, one to products, and one is a guess of a transition state. To verify the structures of the transition states, frequency calculations were carried out at the same level of theory. One imaginary frequency was found in both the HB and FLT pathways. As the reaction proceeded in the acetone, the calculations were performed using reaction field calculation with the integral equation formalism model [SCRF­(CPCM, solvent: acetone)].^49–51^ The Gibbs free energies were calculated at *T* = 298 K. The GIAO calculations of ^31^P chemical shifts for various forms of **5** and **10** were performed at the same level of theory.^52^ As the NMR experiments were performed in CDCl_3_, the calculations were also performed in the chloroform solvent field. PPh_3_ and P­(O)­Ph_3_ were used as reference compounds for chemical shift calculations.

## Supplementary Material



## Data Availability

The data underlying this study are available in the published article and its Supporting Information.

## Notes

Single-crystal X-ray structural data; details of theoretical DFT calculation results; and ^1^H, ^13^C, ^11^B, and ^31^P NMR and mass spectra for all compounds (PDF)
