# Supplementary material for: Formation and Structural Characterization of Benzoxa- and Thienoxaphosphaborinines
Source: J Org Chem. 2025 May 21;90(23):7688–99. doi: 10.1021/acs.joc.5c00501 (PMC12172047; doi:10.1021/acs.joc.5c00501)
Supplement: Supplementary file 1 [file jo5c00501_si_001.pdf]

On the formation and structural characterization of benzoxa- and thienoxaphosphaborinines

Krzysztof Nowicki,* Maja Grądecka, Patrycja Kurasz, Paweł A. Wieczorkiewicz, Damian Dąbrowski, Krzysztof Durka,* Sergiusz Luliński*

Warsaw University of Technology, Faculty of Chemistry, Noakowskiego 3, 00-664 Warsaw, Poland

Supporting Information

List of contents

Crystal structures S2
Theoretical calculations S12
NMR spectra S14
Mass spectra S50

Crystal structures

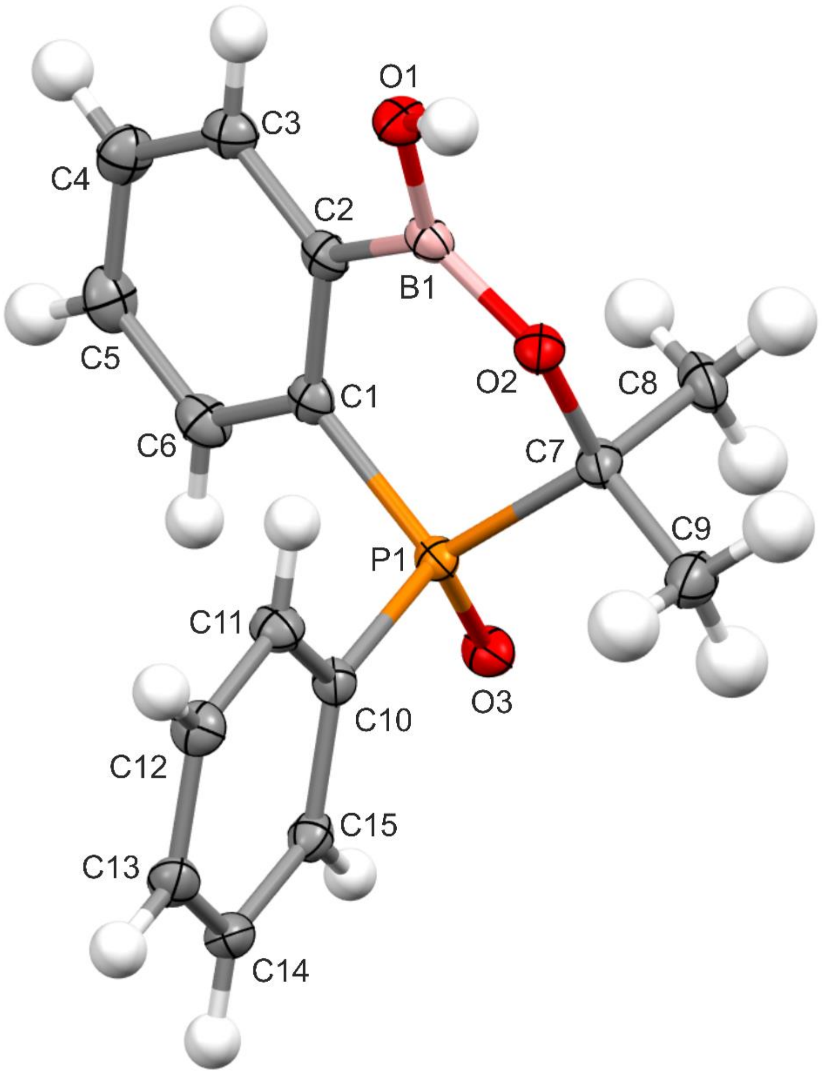

Figure S1. The molecular structure of **11**. Ellipsoids drawn at the 50% probability level. Note that unit cell consist of two symmetrically independent molecules (**11A**, **11B**) and only one molecule has been shown (**11A**). The conformation molecule **11B** is very similar.

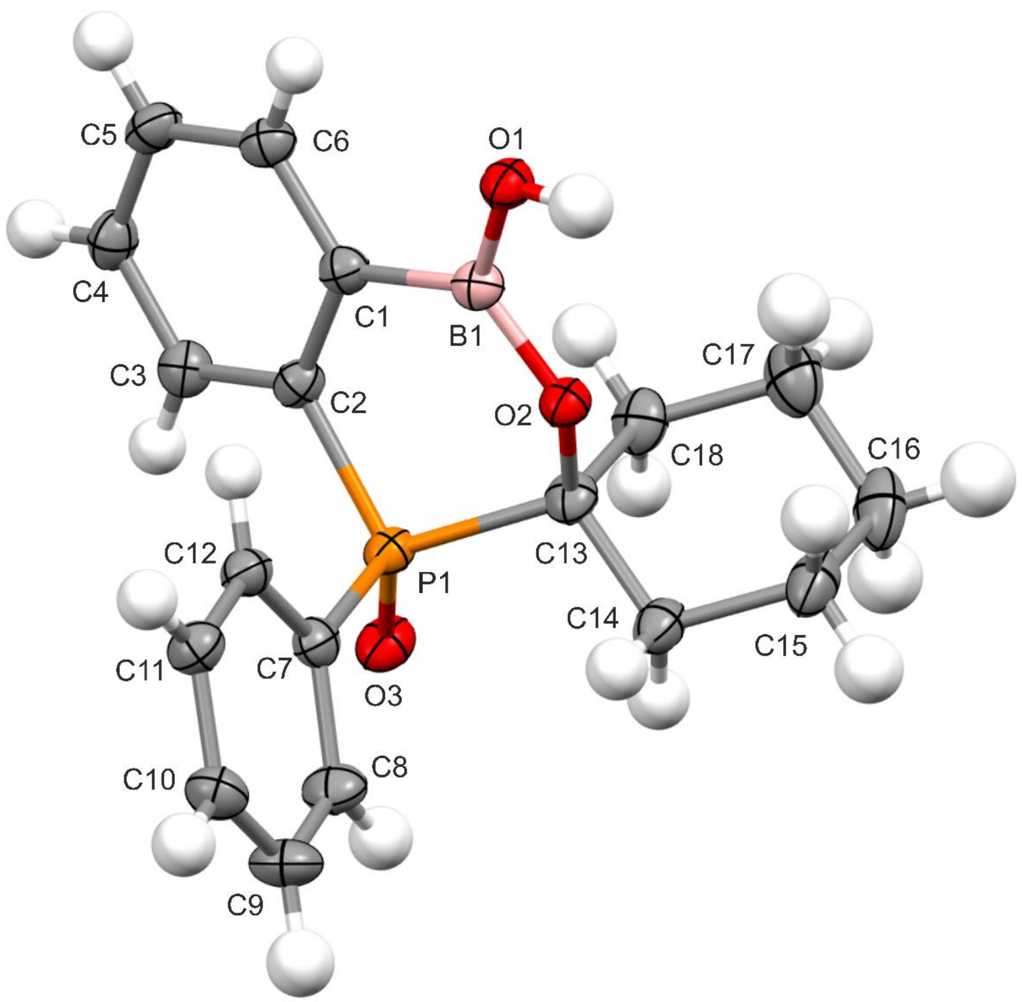

Figure S2. The molecular structure of **13**. Ellipsoids drawn at the 50% probability level. Note that unit cell consist of two symmetrically independent molecules (**13A-C**) and only one molecule has been shown (**13A**). The conformations of remaining molecules **13** are almost identical.

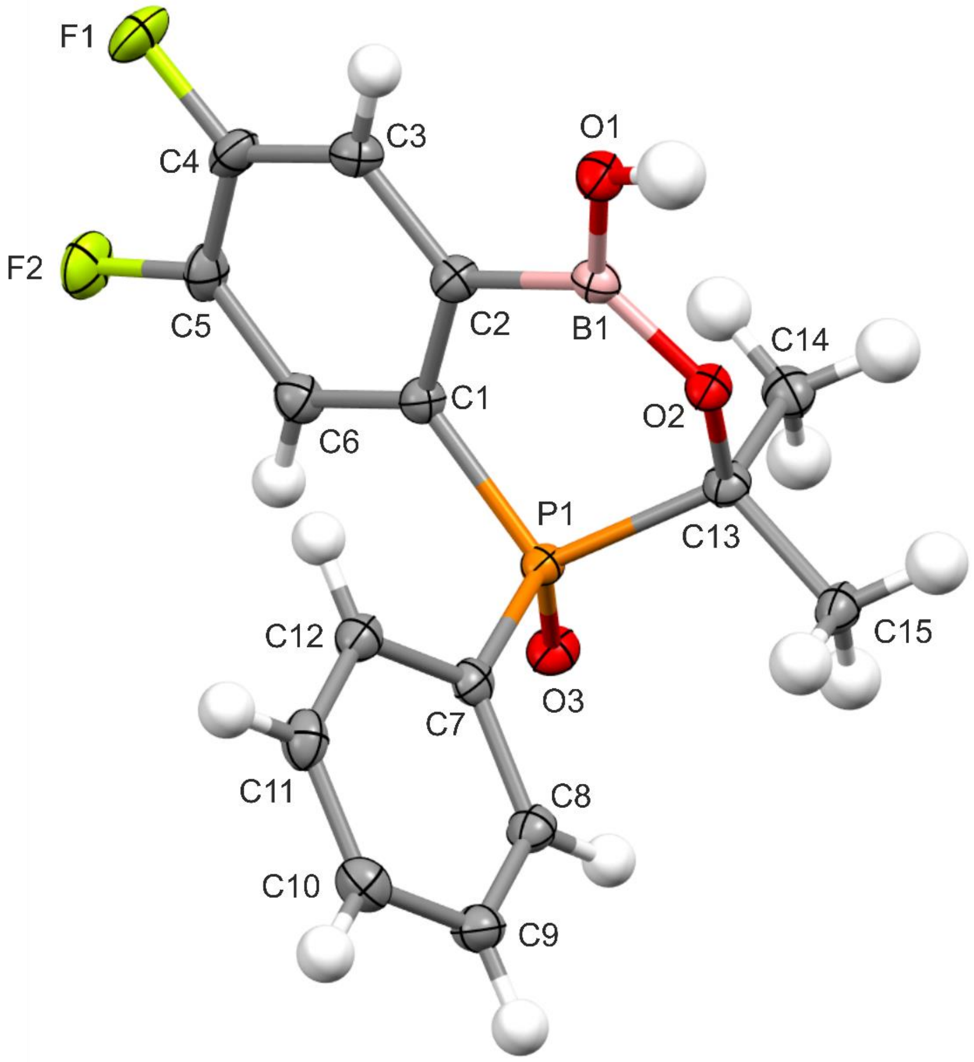

Figure S3. The molecular structure of **20**. Ellipsoids drawn at the 50% probability level.

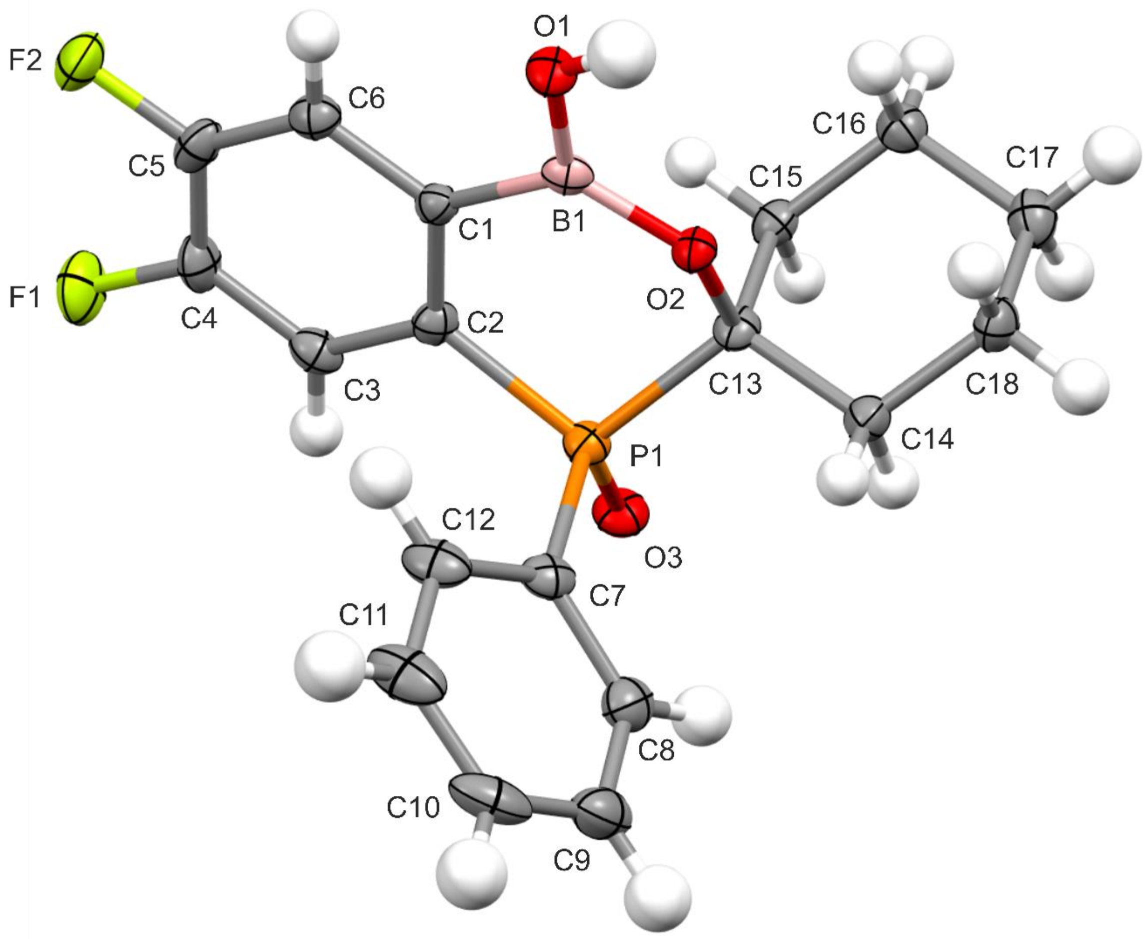

Figure S4. The molecular structure of **22**. Ellipsoids drawn at the 50% probability level.

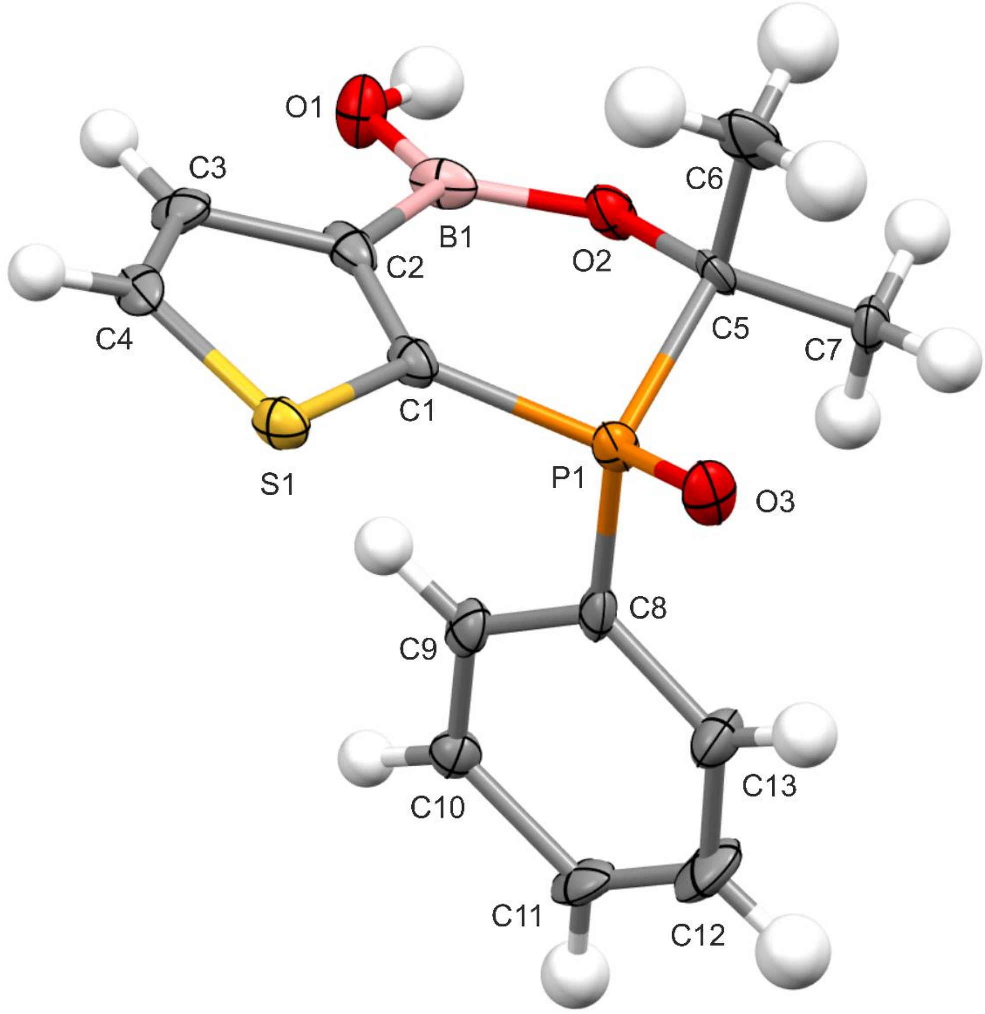

Figure S5. The molecular structure of **23**. Ellipsoids drawn at the 50% probability level. Note that unit cell consist of eight symmetrically independent molecules (**23A-H**) and two molecules of chloroform. For clarity only molecule **23A** is presented. The conformations of remaining molecules **23** are almost identical.

Table S1. Selected crystal data, data collection and refinement parameters for **11**, **13**, **20**, **22** and **23**.

	**11**	**13**	**20**	**21**	**23**
Empirical formula	$C_{15}H_{16}O_3PB$	$C_{18}H_{20}BO_3P$	$C_{15}H_{14}BO_3F_2P$	$C_{18}H_{18}BO_3PF_2$	$C_{53}H_{53}B_4P_4S_4O_{12}Cl_3$
Formula weight	286.06	326.12	322.04	362.10	1283.66
Crystal system	triclinic	monoclinic	monoclinic	monoclinic	tetragonal
Space group	*P*-1	$P2_1/c$	$P2_1/c$	$P2_1/c$	$P4_1$
a / Å	10.1529(4)	12.9786(2)	12.1685(4)	14.9461(9)	16.69530(10)
b / Å	11.7946(5)	20.1338(3)	7.7983(3)	7.7181(3)	16.69530(10)
c / Å	13.0022(5)	19.4885(3)	16.3160(5)	14.5659(10)	47.1117(4)
α / °	79.863(4)	90	90	90	90
β / °	76.170(4)	98.4840(10)	108.590(4)	97.324(7)	90
γ / °	75.913(4)	90	90	90	90
Volume / $Å^3$	1454.88(11)	5036.78(13)	1467.50(9)	1666.55(17)	13131.58(19)
Z	4	12	4	4	8
ρ_{calc} gcm^{-3}	1.306	1.290	1.458	1.443	1.299
μ/mm^{-1}	1.703	1.540	1.959	1.790	3.822
F(000)	600.0	2064.0	664.0	752.0	5296.0
Crystal size/mm^3	0.12 × 0.11 × 0.10	0.035 × 0.024 × 0.022	0.13 × 0.13 × 0.10	0.120 × 0.069 × 0.043	0.221 × 0.122 × 0.085
Radiation	CuKα (λ = 1.54178)	Cu Kα (λ = 1.54184)	Cu Kα (λ = 1.54184)	Cu Kα (λ = 1.54184)	Cu Kα (λ = 1.54184)
2Θ range for data collection/°	7.058 to 141.416	6.348 to 141.896	7.664 to 145.26	5.962 to 122.822	5.294 to 145.522
Index ranges	$-10 \le h \le 12$, $-13 \le k \le 14$, $-15 \le l \le 14$	$-15 \le h \le 15$, $-23 \le k \le 24$, $-23 \le l \le 20$	$-14 \le h \le 14$, $-8 \le k \le 9$, $-19 \le l \le 16$	$-16 \le h \le 16$, $-8 \le k \le 4$, $-16 \le l \le 15$	$-15 \le h \le 18$, $-20 \le k \le 20$, $-58 \le l \le 58$
Reflections collected	15966	27295	10008	4998	69338
Independent reflections	5503 [R_{int} = 0.0219, R_{sigma} = 0.0219]	9532 [R_{int} = 0.0309, R_{sigma} = 0.0323]	2849 [R_{int} = 0.0292, R_{sigma} = 0.0283]	2532 [R_{int} = 0.0406, R_{sigma} = 0.0718]	24644 [R_{int} = 0.0309, R_{sigma} = 0.0298]
Data/restraints/parameters	5503/2/369	9532/0/622	2849/1/203	2532/1/230	24644/55/1458
Goodness-of-fit	1.021	1.023	1.058	1.025	1.039
Final *R* indexes [I>=2σ (I)]	R_1 = 0.0304, wR_2 = 0.0766	R_1 = 0.0369, wR_2 = 0.0862	R_1 = 0.0329, wR_2 = 0.0807	R_1 = 0.0485, wR_2 = 0.0973	R_1 = 0.0712, wR_2 = 0.1974
Final *R* indexes [all data]	R_1 = 0.0342, wR_2 = 0.0793	R_1 = 0.0507, wR_2 = 0.0932	R_1 = 0.0405, wR_2 = 0.0847	R_1 = 0.0844, wR_2 = 0.1105	R_1 = 0.0771, wR_2 = 0.2060
Largest diff. peak/hole / e $Å^{-3}$	0.45/–0.36	0.41/–0.33	0.31/–0.37	0.27/–0.32	2.21/–0.90

Table S2. Selected bond distances in crystal structures **11**, **13**, **20**, **21** and **23**.

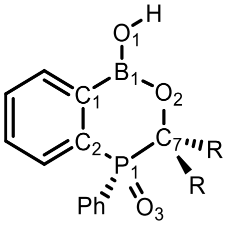

	$d_{P1\text{-}C1}$ /Å	$d_{P1\text{-}C7}$ /Å	$d_{P1\text{-}O3}$ /Å	$d_{B1\text{-}O1}$ /Å	$d_{B1\text{-}O2}$ /Å	$d_{B1\text{-}C2}$ /Å	$d_{O2\text{-}C7}$ /Å
**11A**	1.803(2)	1.852(1)	1.496(1)	1.344(2)	1.378(2)	1.573(2)	1.449(2)
**11B**	1.796(2)	1.853(1)	1.494(1)	1.346(2)	1.377(2)	1.572(2)	1.446(2)
**13A**	1.800(2)	1.843(2)	1.488(1)	1.346(2)	1.377(2)	1.574(3)	1.448(2)
**13B**	1.799(2)	1.837(2)	1.491(1)	1.347(2)	1.378(2)	1.576(3)	1.442(2)
**13C**	1.797(2)	1.851(2)	1.492(1)	1.338(3)	1.375(3)	1.571(3)	1.443(2)
**20**	1.802(1)	1.842(2)	1.493(1)	1.342(3)	1.371(2)	1.579(2)	1.451(2)
**22**	1.800(3)	1.844(3)	1.494(2)	1.348(5)	1.374(5)	1.571(5)	1.455(3)
**23A**	1.78(1)	1.858(8)	1.492(6)	1.31(1)	1.41(1)	1.57(1)	1.421(9)
**23B**	1.761(7)	1.868(7)	1.503(5)	1.380(9)	1.400(9)	1.56(1)	1.409(8)
**23C**	1.778(7)	1.864(7)	1.507(5)	1.385(9)	1.395(9)	1.55(1)	1.426(8)
**23D**	1.791(8)	1.846(7)	1.493(5)	1.39(1)	1.34(1)	1.58(1)	1.483(9)
**23E**	1.779(7)	1.839(7)	1.485(5)	1.30(1)	1.37(1)	1.59(1)	1.479(8)
**23F**	1.795(7)	1.842(7)	1.482(5)	1.30(1)	1.38(1)	1.56(1)	1.478(8)
**23G**	1.775(8)	1.851(7)	1.500(5)	1.34(1)	1.39(1)	1.56(1)	1.47(1)
**23H**	1.758(8)	1.853(7)	1.491(5)	1.32(1)	1.39(1)	1.57(1)	1.414(8)

Table S3. Bond angles (°) within phosphoxaborinine ring.

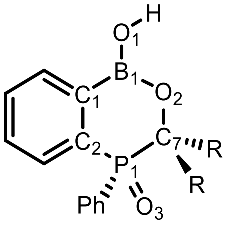

	$\alpha_{C1\text{-}B1\ldots O1}$ / °	$\alpha_{C1\text{-}B1\ldots O2}$ / °	$\alpha_{B1\text{-}O2\ldots C7}$ / °	$\alpha_{O2\text{-}C7\ldots P1}$ / °	$\alpha_{C7\text{-}P1\ldots C2}$ / °	$\alpha_{C1\text{-}C2\ldots P1}$ / °	$\alpha_{C1\text{-}C2\ldots B2}$ / °
**11A**	118.1(1)	122.2(1)	123.3(1)	110.1(1)	103.62(7)	120.1(1)	120.4(1)
**11B**	118.3(1)	122.2(1)	123.2(1)	110.75(9)	103.45(7)	120.2(1)	120.8(1)
**13A**	118.0(2)	122.2(2)	122.9(1)	110.2(1)	103.76(8)	118.8(1)	122.0(2)
**13B**	120.0(2)	121.8(2)	123.4(1)	108.7(1)	102.53(8)	118.7(1)	121.1(2)
**13C**	117.9(2)	121.8(2)	123.8(1)	110.8(1)	104.20(8)	119.2(1)	122.3(2)
**20**	116.8(2)	121.9(2)	123.7(1)	109.6(1)	102.54(8)	118.5(1)	121.6(1)
**22**	117.7(3)	122.4(3)	122.7(3)	109.7(2)	103.2(1)	118.9(2)	121.6(3)
**23A**	118.3(7)	120.3(7)	124.3(6)	110.8(5)	102.8(4)	122.4(7)	121.3(7)
**23B**	121.0(6)	122.4(6)	123.8(5)	111.1(4)	101.7(3)	121.7(5)	118.9(6)
**23C**	120.7(6)	122.2(6)	124.2(5)	109.7(5)	101.0(3)	121.0(5)	118.7(6)
**23D**	118.2(7)	121.7(7)	121.5(6)	109.1(5)	101.3(3)	120.4(6)	121.1(7)
**23E**	119.4(7)	118.6(7)	124.0(6)	109.6(4)	100.2(3)	121.7(6)	122.2(7)
**23F**	119.7(7)	119.2(7)	123.8(6)	110.3(5)	100.3(3)	120.7(5)	123.8(7)
**23G**	116.3(8)	122.6(8)	121.4(6)	111.1(5)	101.5(4)	121.7(6)	121.4(7)
**23H**	119.6(7)	120.3(7)	123.3(6)	111.0(5)	100.4(3)	122.9(6)	119.5(7)

Table S4. Geometry of hydrogen bond interactions in studied structures.

	Interaction	$d_{O...O}$ / Å	$d_{H...O}$ / Å	$\alpha_{O\text{-}H...O}$ / °	Symmetry (#)
**11**	O1–H1A…O6#	2.643(1)	1.82(1)	165(2)	x,y,z
	O4–H4A…O3#	2.651(2)	1.83(2)	169(2)	x,-1+y,z
**13**	O7–H7…O6#	2.618(2)	1.80(2)	164(2)	x,y,z
	O4–H4…O3#	2.707(2)	1.89(2)	165(2)	1-x,1/2+y,1/2-z
	O1–H1…O9#	2.612(2)	1.80(2)	163(2)	1-x,1/2+y,1/2-z
**20**	O1–H1A…O3#	2.653(2)	1.83(2)	165(2)	x,-1+y,z
**22**	O1–H1A…O3#	2.670(3)	1.85(2)	168(3)	x,-1+y,z
**23**	O1–H1…O15#	2.666(8)	1.85(4)	163(4)	x,1+y,-1+z
	O4–H4…O12#	2.685(7)	1.85(4)	172(4)	-y,x,1/4+z
	O7–H7…O19#	2.645(7)	1.83(4)	165(4)	x,-1+y,z
	O10–H1…O9#	2.628(8)	1.82(4)	161(4)	1-x,-y,-1/2+z
	O13–H13…O3#	2.685(7)	1.85(4)	171(4)	y,1-x,3/4+z
	O16–H16…O24#	2.731(7)	1.89(4)	176(4)	y,1-x,-1/4+z
	O21–H21…O6#	2.600(8)	1.80(4)	158(4)	y,1-x,3/4+z
	O22–H22…O18#	2.710(8)	1.91(4)	158(4)	x,y,z

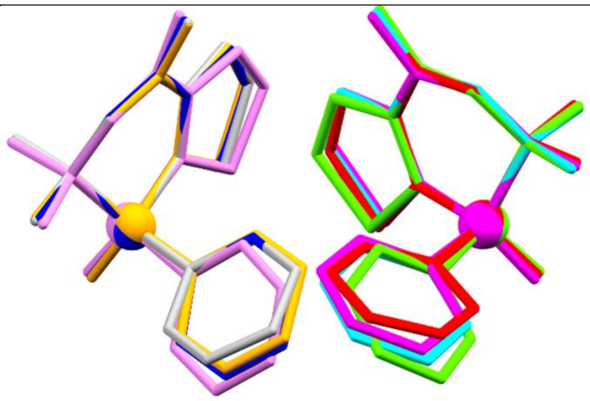

Figure S6. Overlay of molecules (R and S configurations) constituting the asymmetric part of the unit cell of structure **23**.

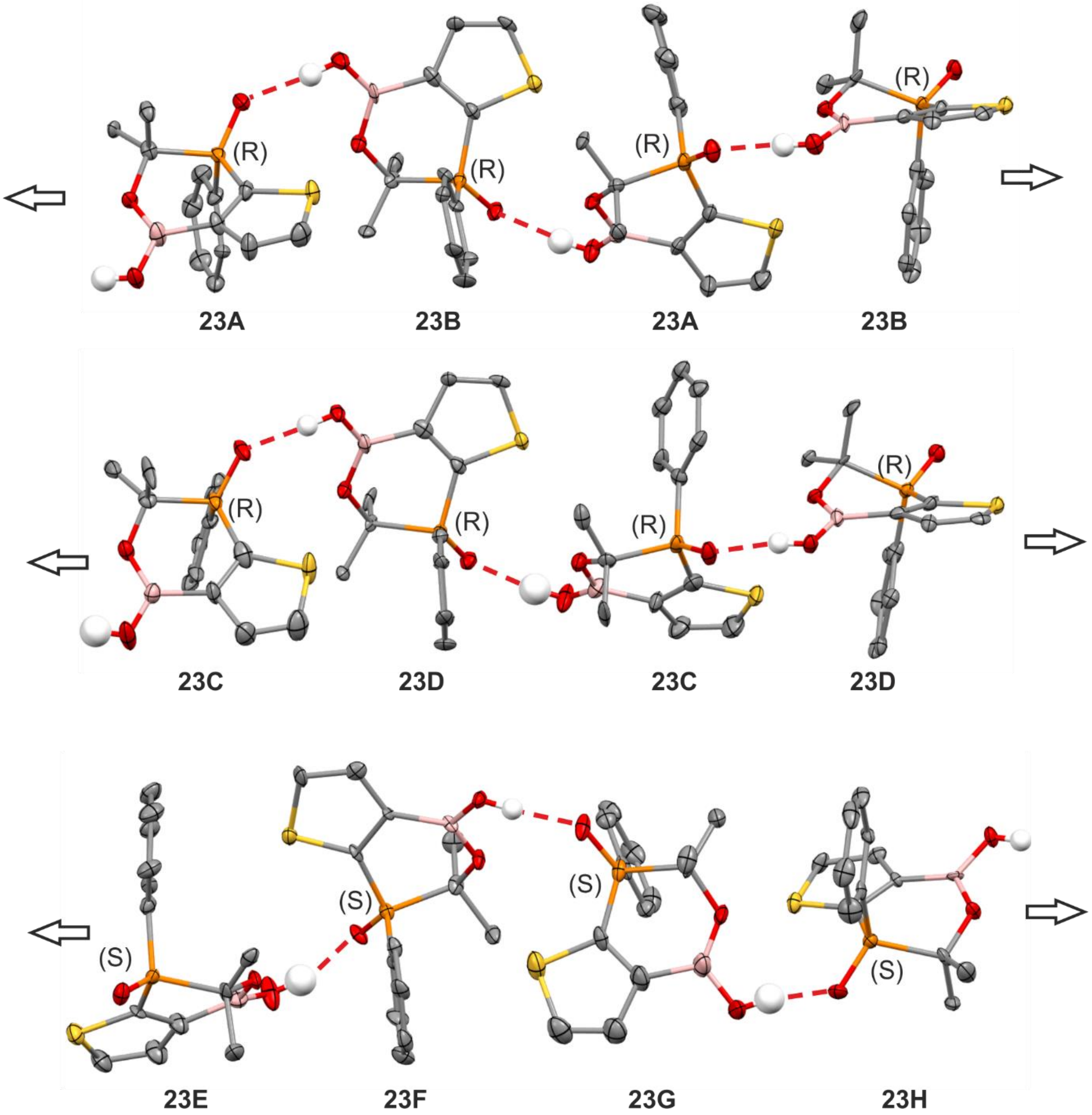

Figure S7. Hydrogen bonded chains in **23**.

Theoretical calculations

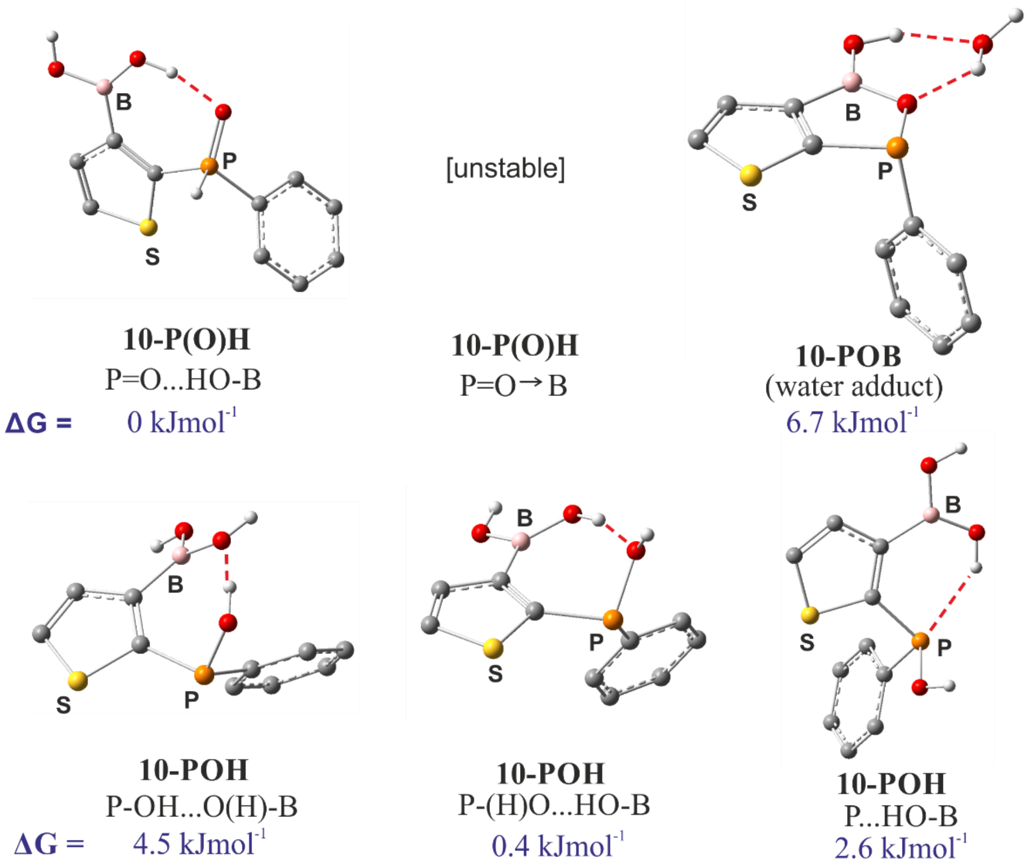

Figure S8. Various forms of **10**. Geometries were derived from theoretical calculations (M06-2X/6-311++G(d,p)), relative free enthalpy values (Δ*G*) are given with respect to the most stable 10-**P(O)H** tautomer (stabilized through P=O…HO-B intramolecular HB)

Table S5. Computed total energy values and number of imaginary frequencies for the optimized structures (M062X/6-311++G(d,p)).

Compound	G / kJmol^{-1}	N. im. Freq.	Compound	G / kJmol^{-1}	N. im. Freq.
**5-P(O)H** (P=O…HO−B)	−2773171.79788	0	**10-P(O)H** (P=O…HO−B)	−3615453.57430	0
**5-P(O)H** (P=O→B)	−2773171.78923	0	**10-POH** (P−OH…O(H)−B)	−3615453.15684	0
**5-POH** (P−OH…O(H)−B)	−2773162.11241	0	**10-POH** (P−(H)O…HO−B)	−3615449.05319	0
**5-POH** (P–(H)O…HO–B)	−2773166.58626	0	**10-POH** (P…HO–B)	−3615450.92517	0
**5-POH** (P…HO–B)	−2773159.83873	0	**10-POB** (water adduct)	−3615447.09456	0
**5-POB** (water adduct)	−2773181.50698	0	**10-POB**	−3414813.03894	0
**5-POB**	−2572547.54587	0	**5** + acetone (HB)	−3280114.67175	0
**TS-HB**	−3280082.78325	1	**TS-FLP**	−3280055.29425	1
**P-HB**	−3280159.74038	0	**P-FLP**	−3280115.36475	0
**11**	−3079526.87963	0			

NMR spectra

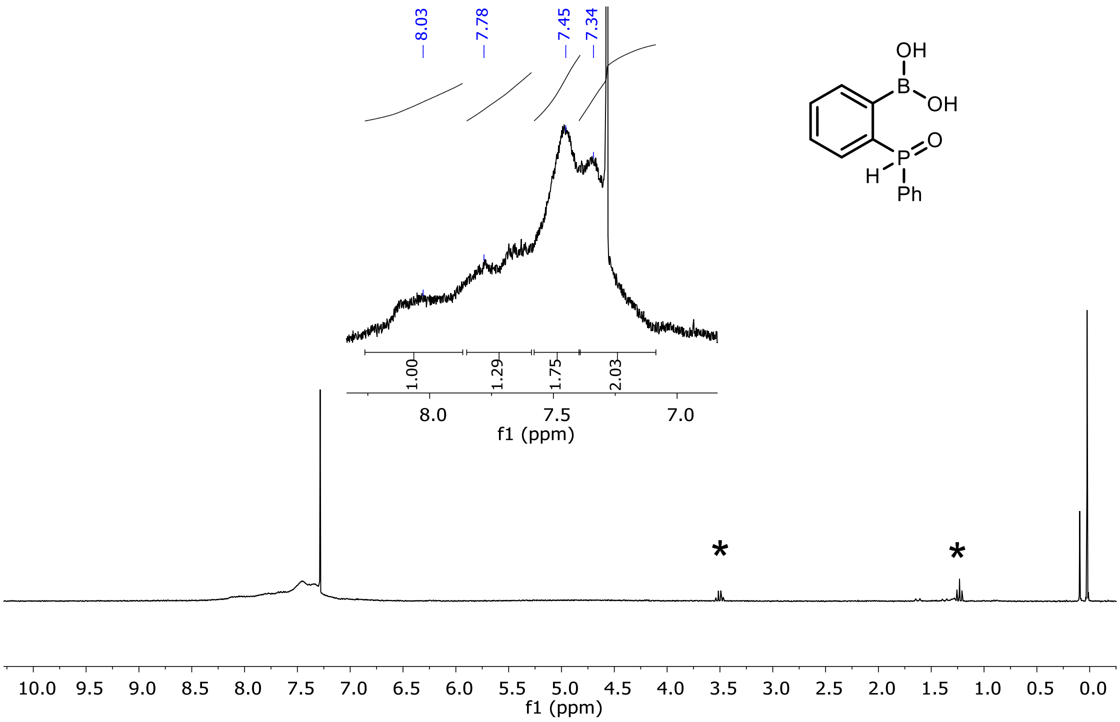

Figure S9. ^{1}H NMR spectrum (400 MHz, $CDCl_3$) of **5.** The signals of a residual solvent (Et_2O) are marked with asterisks.

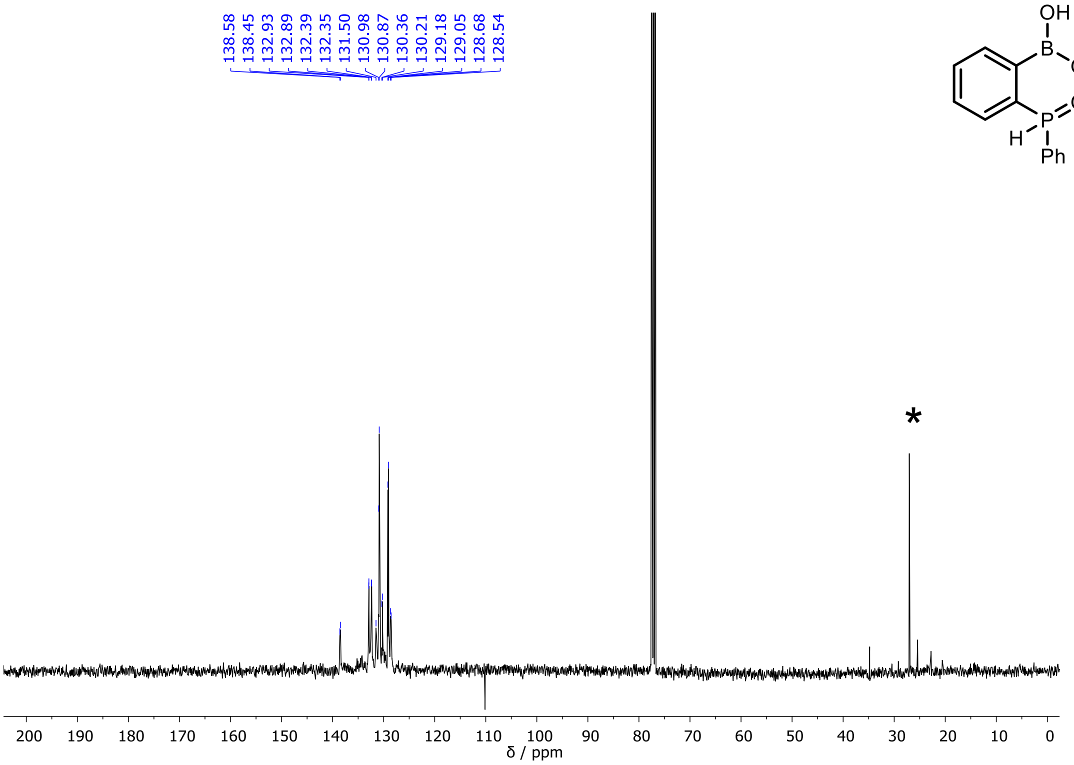

Figure S10. $^{13}C\{^{1}H\}$ NMR spectrum (101 MHz, $CDCl_3$) of **5**. The signals of a residual solvent (cyclohexane) is marked with an asterisk.

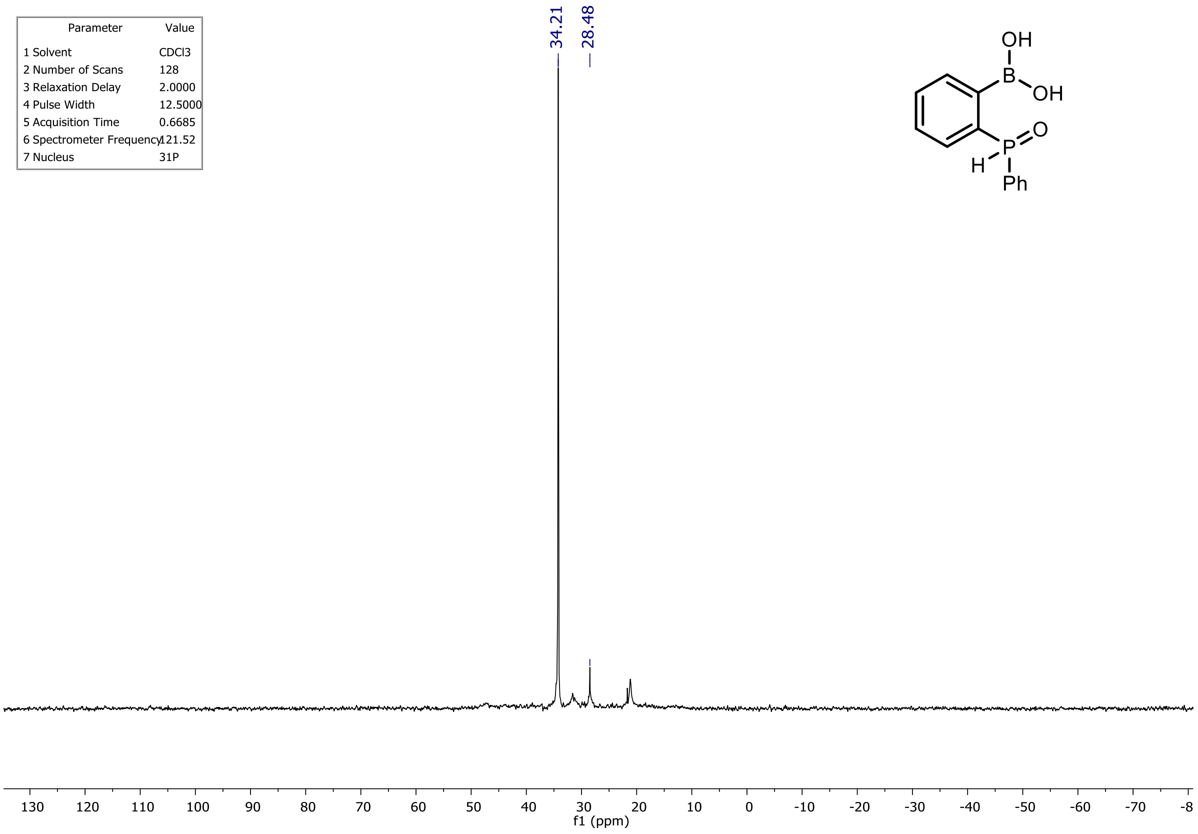

Figure S11. $^{31}P\{^{1}H\}$ NMR spectrum (162 MHz, $CDCl_3$) of **5**.

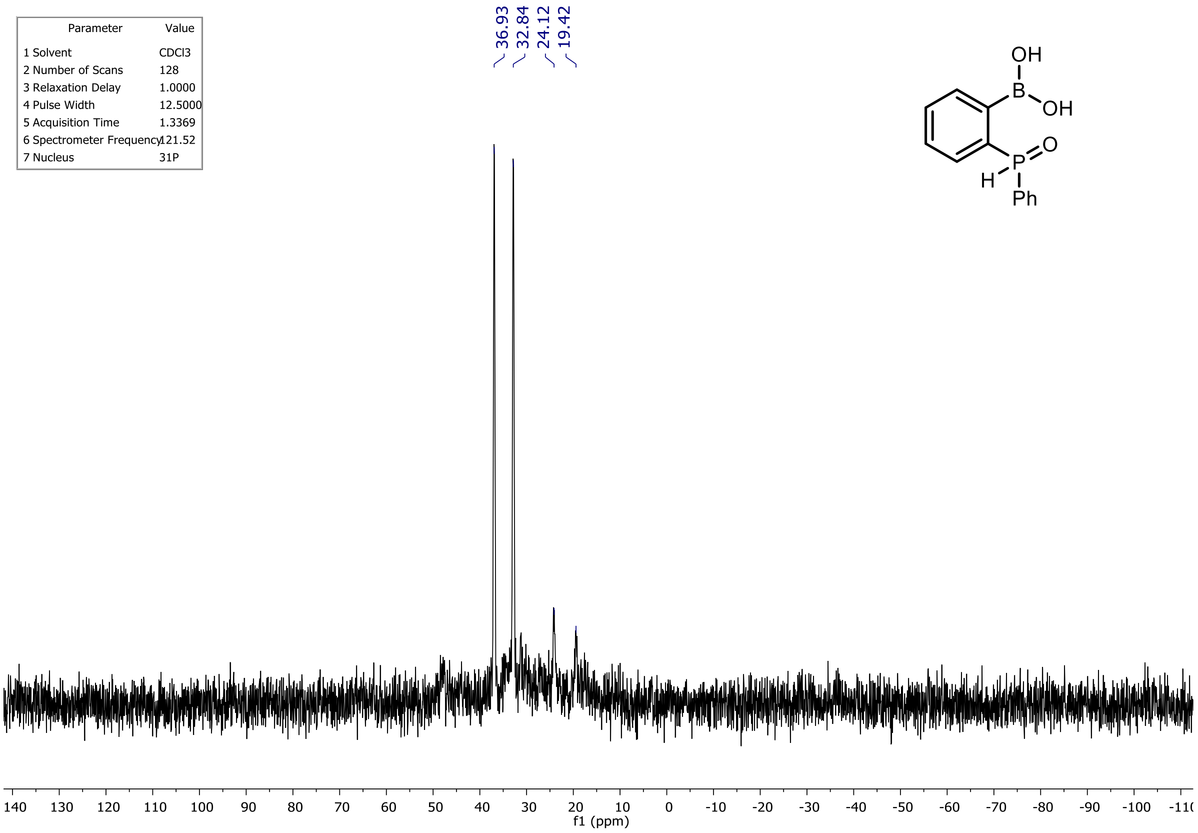

Figure S12. ^{31}P NMR spectrum (162 MHz, $CDCl_3$) of **5**.

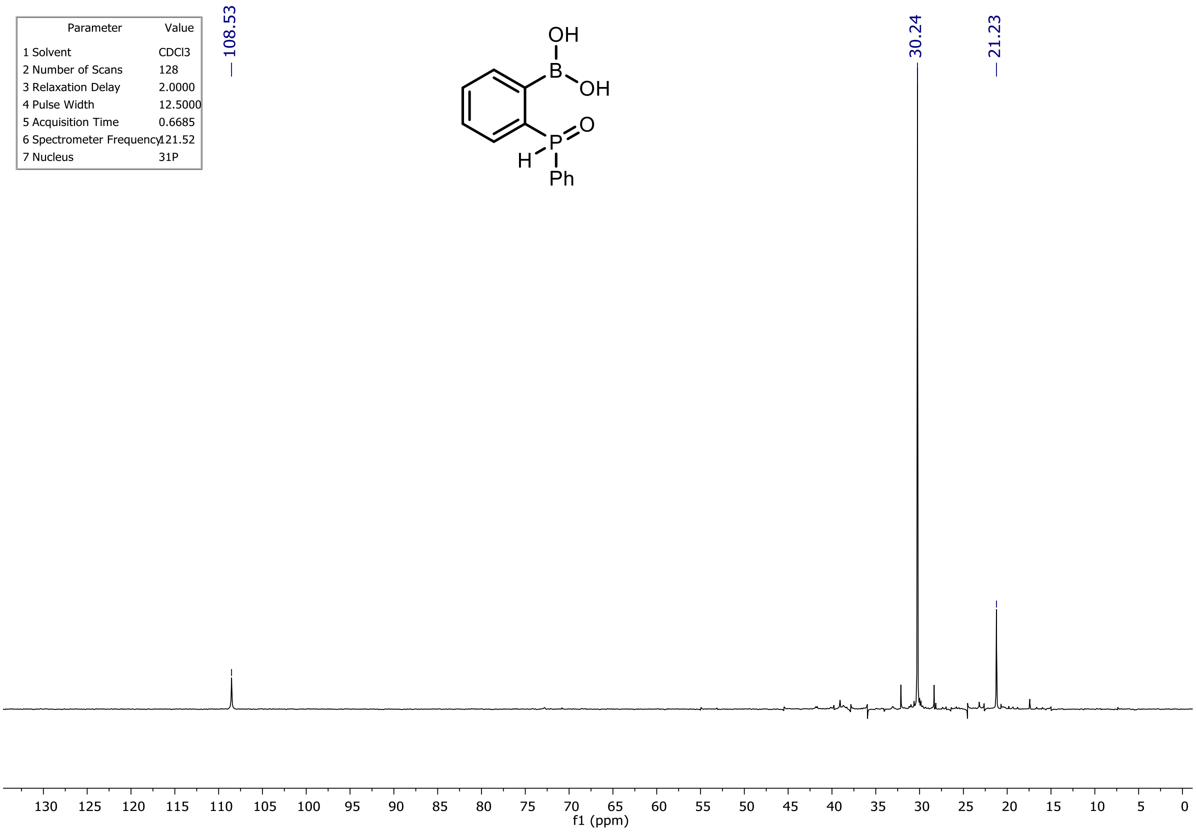

Figure S13. $^{31}P\{^{1}H\}$ NMR spectrum (162 MHz, DMSO-d_6) of **5**.

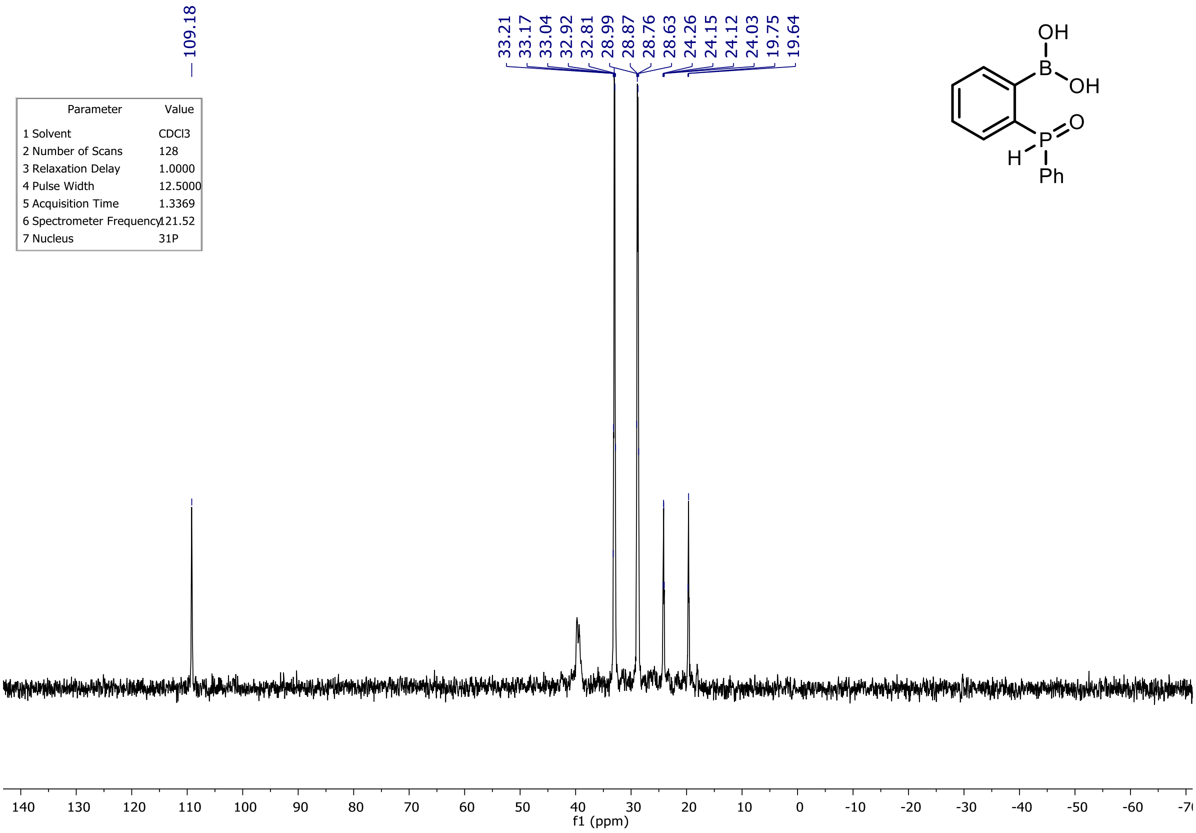

Figure S14. ^{31}P NMR spectrum (162 MHz, DMSO-d_6) of **5**.

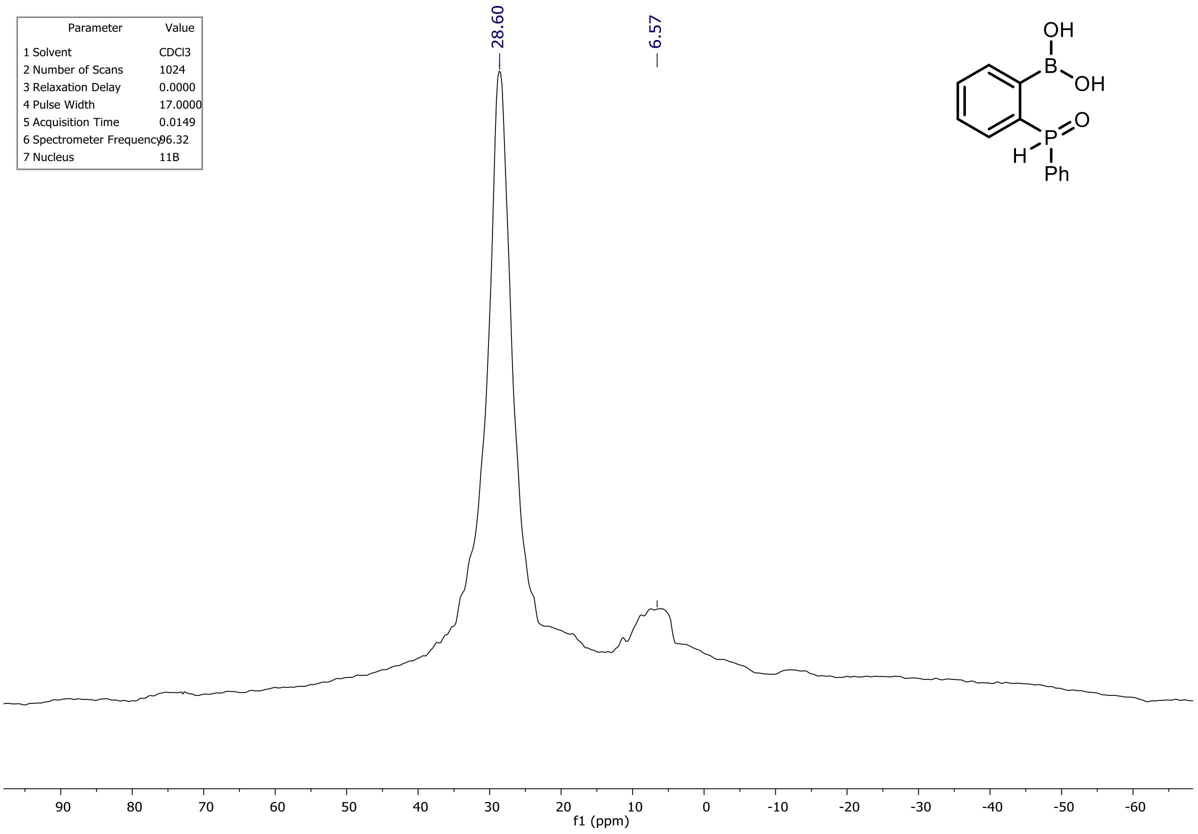

Figure S15. ^{11}B NMR spectrum (96 MHz, DMSO-d_6) of **5**.

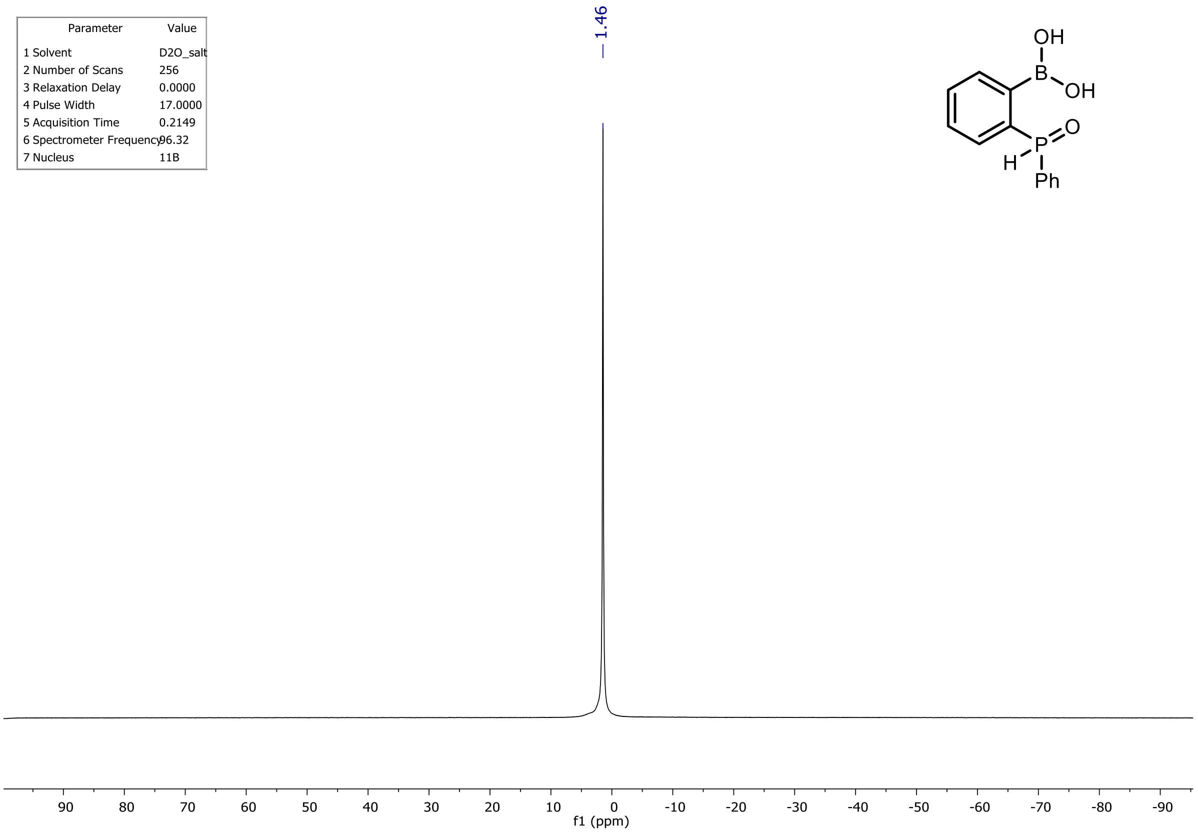

Figure S16. ^{31}B NMR spectrum (96 MHz, D_2O/NaOH) of **5**.

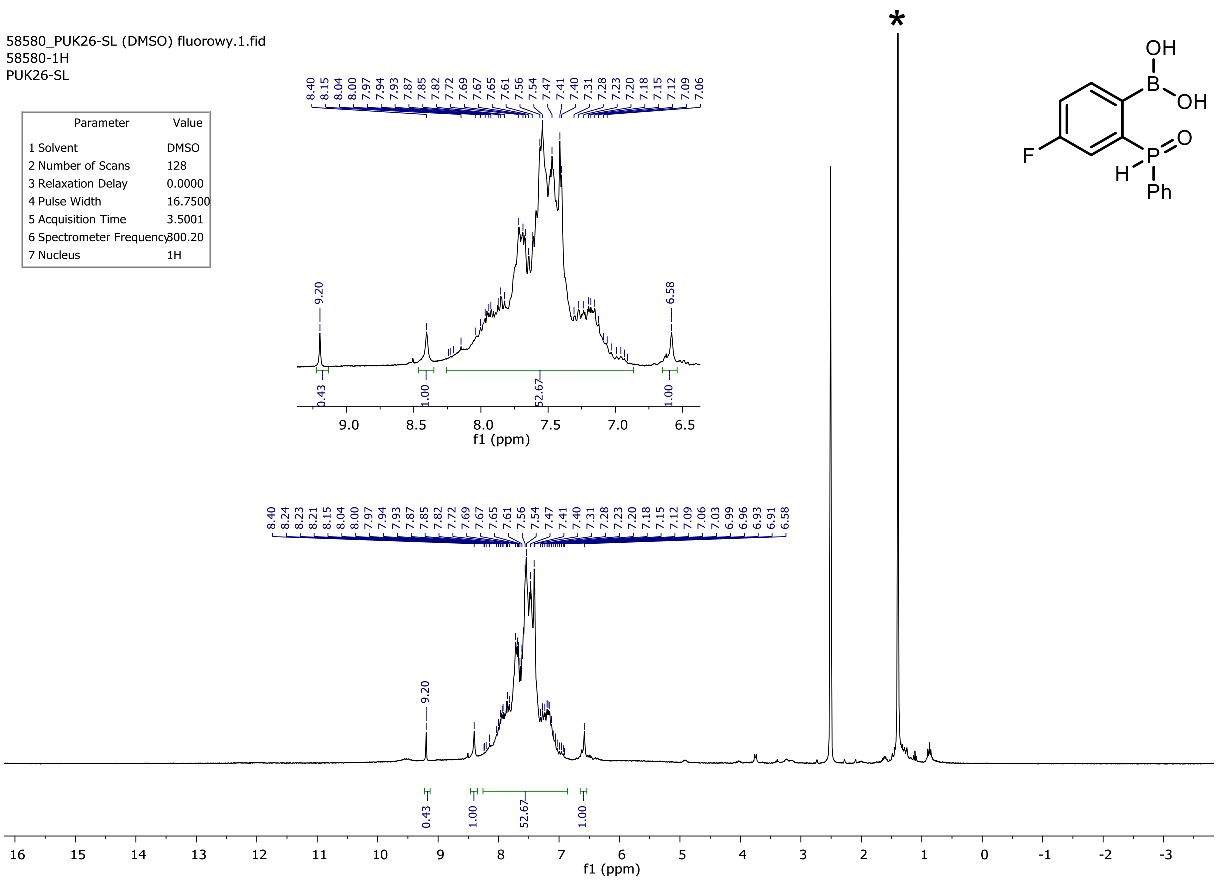

Figure S17. ^{1}H NMR spectrum (300 MHz, DMSO-d_6) of **6**. The signal of a residual solvent (cyclohexane) is marked with an asterisk.

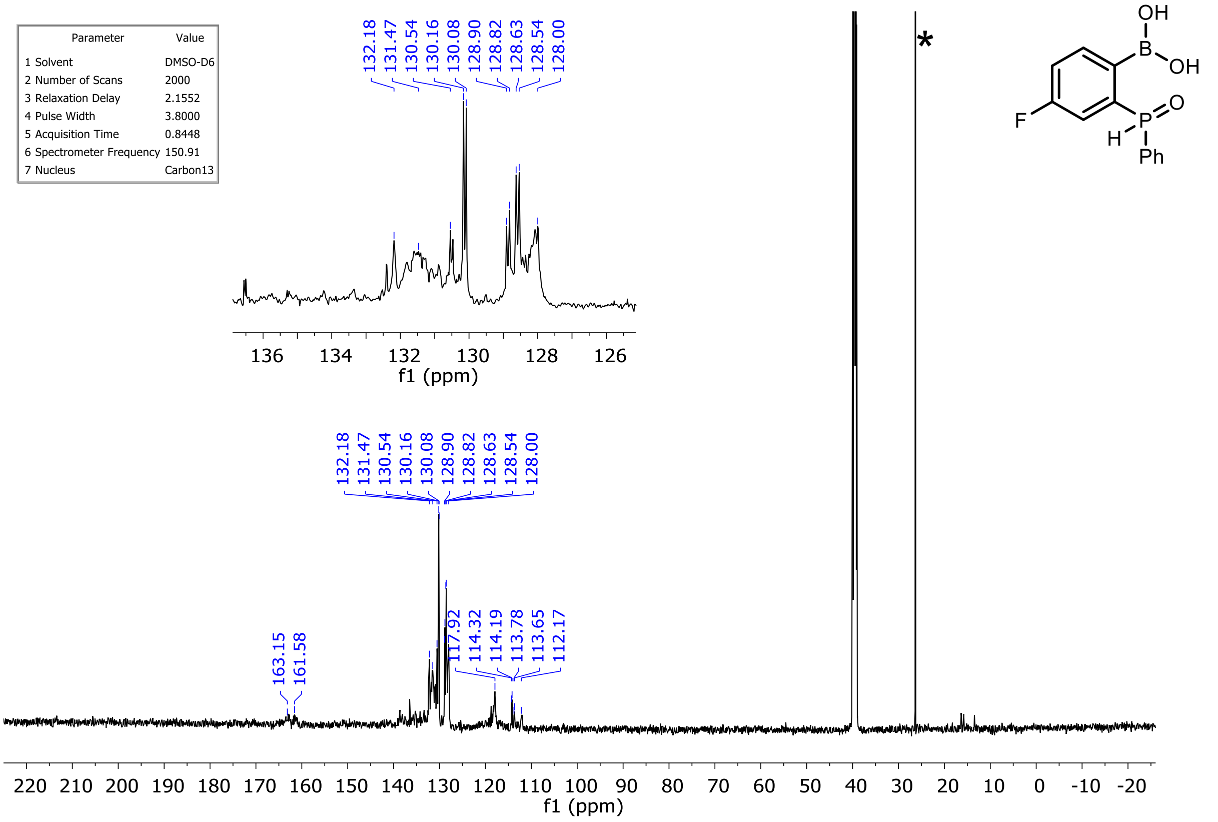

Figure S18. $^{13}C\{^{1}H\}$ NMR spectrum (101 MHz, DMSO-d_6) of **6**. The signal of a residual solvent (cyclohexane) is marked with an asterisk.

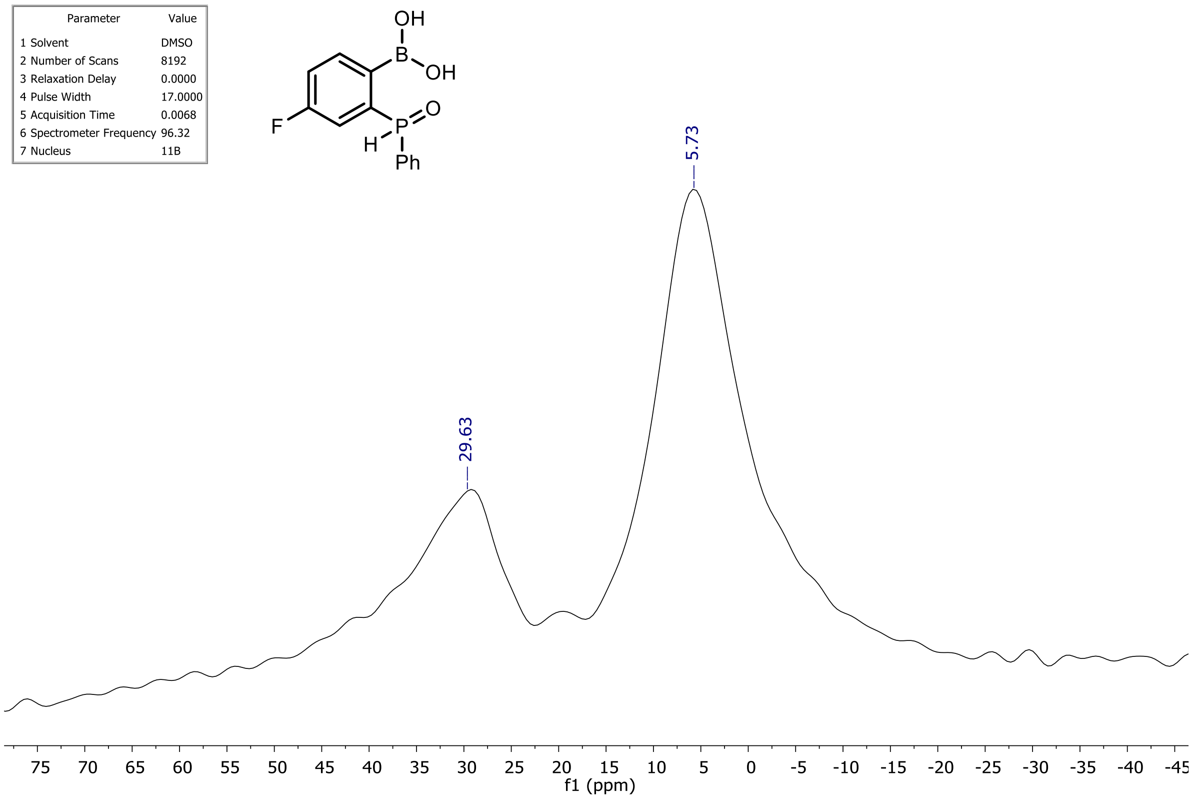

Figure S19. ^{11}B NMR spectrum (96 MHz, DMSO-d_6) of **6**.

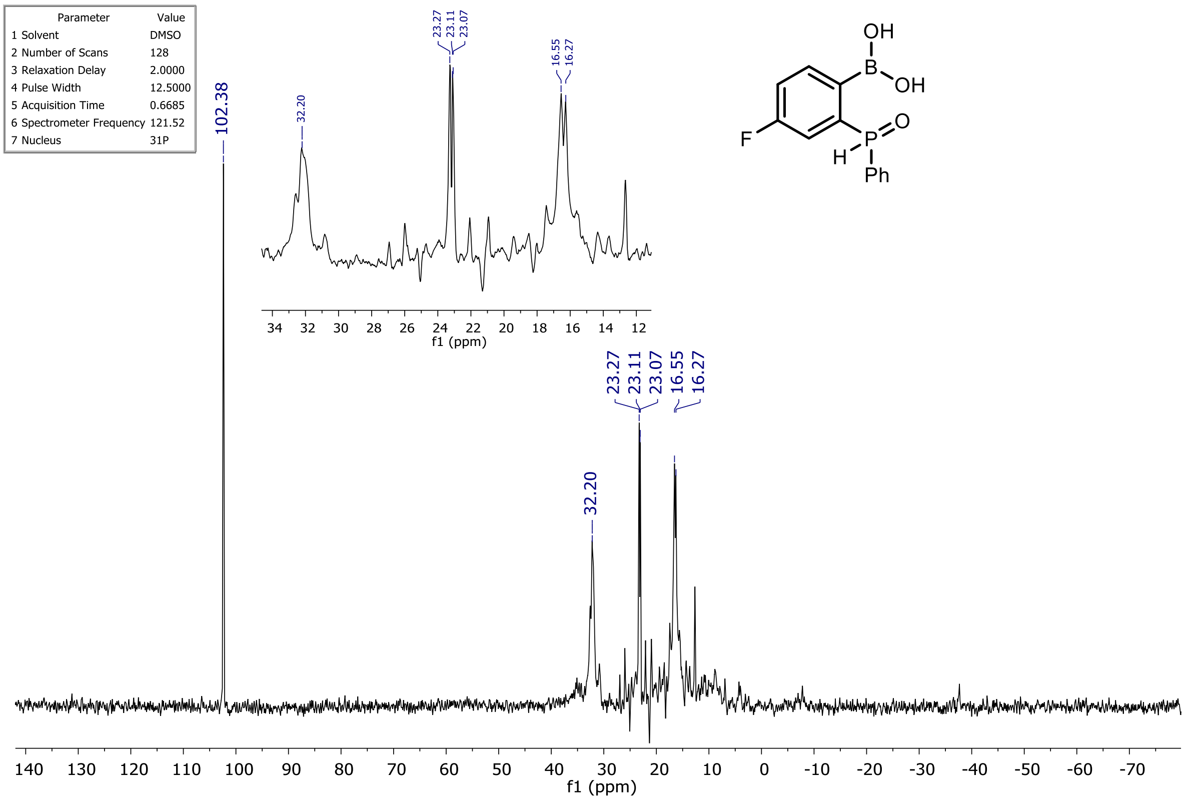

Figure S20. $^{31}P\{^{1}H\}$ NMR spectrum (122 MHz, $CDCl_3$) of **6**.

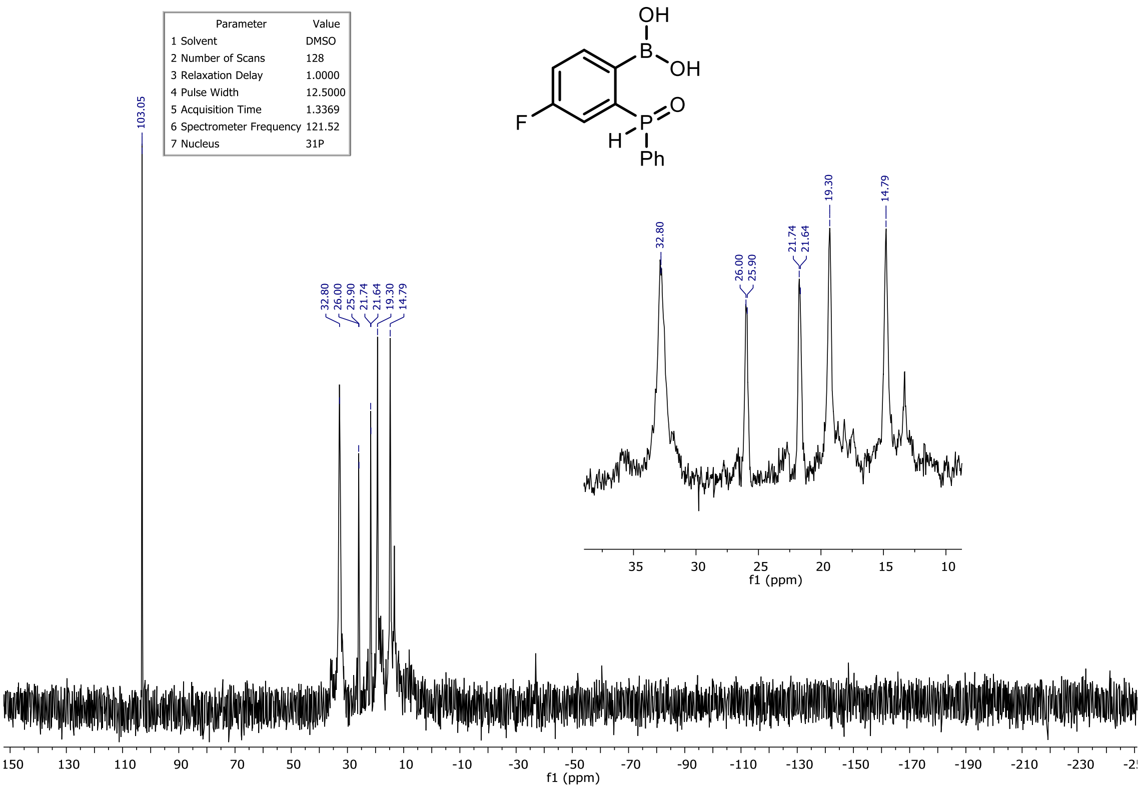

Figure S21. ^{31}P NMR spectrum (122 MHz, DMSO-d_6) of **6**.

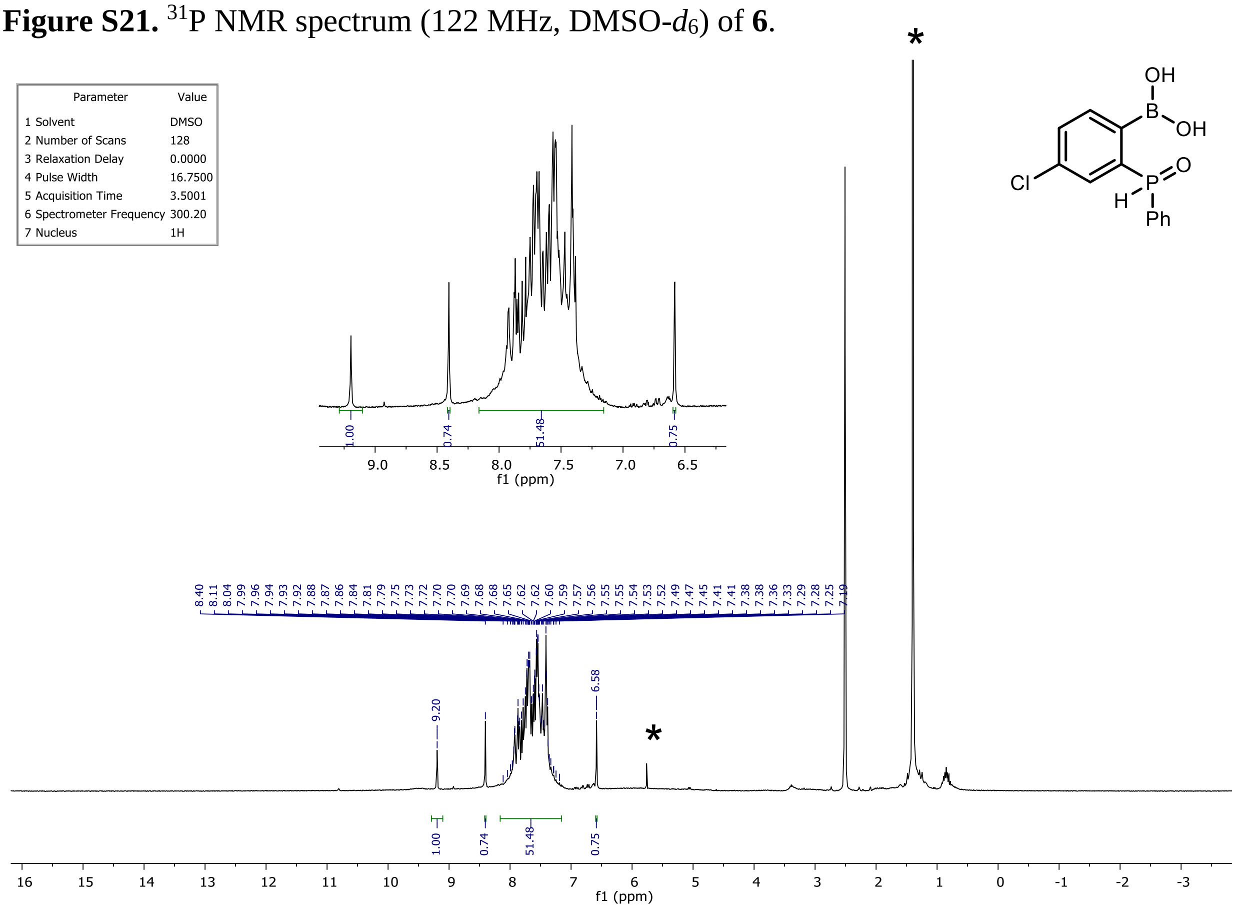

Figure S22. ^{1}H NMR spectrum (300 MHz, DMSO-d_6) of **7**. The signals of residual solvents (DCM and cyclohexane) are marked with asterisks.

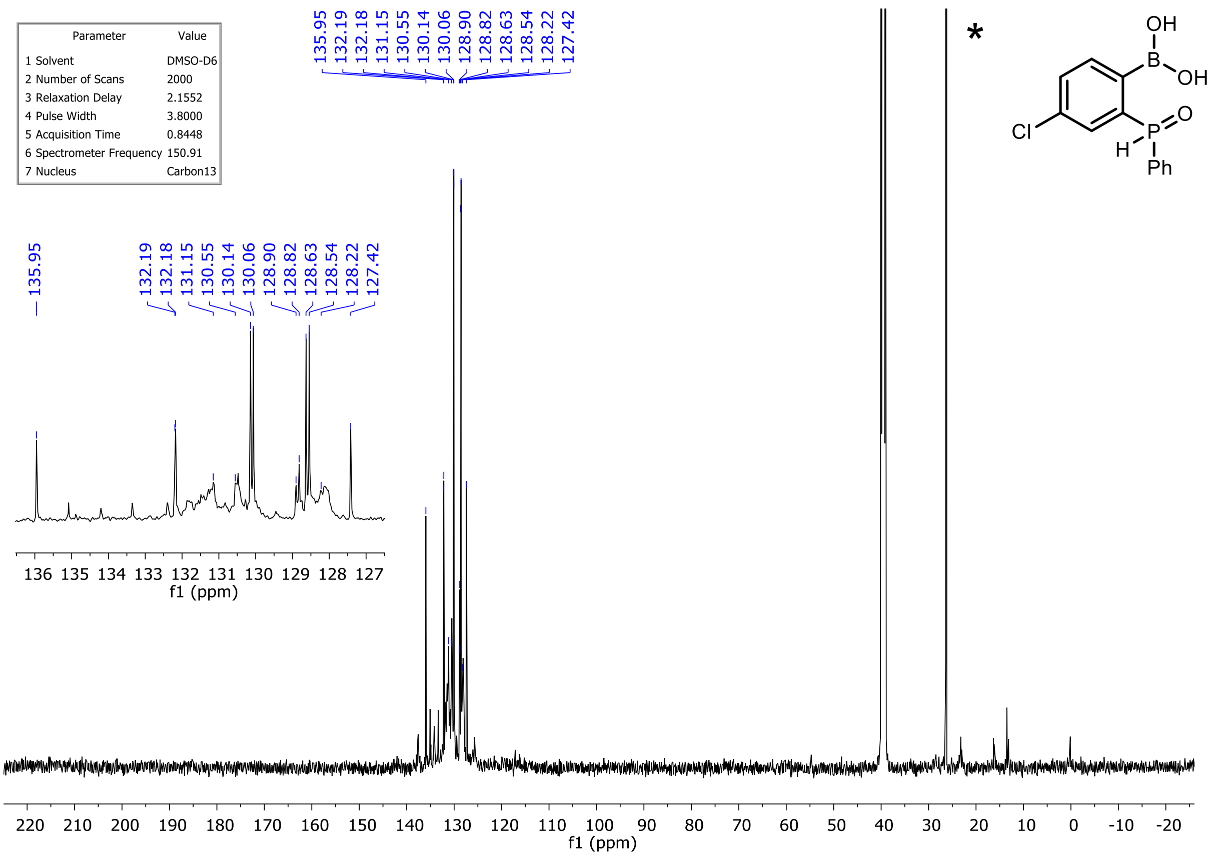

Figure S23. $^{13}C\{^1H\}$ NMR spectrum (101 MHz, DMSO-d_6) of **7**. The signal of a residual solvent (cyclohexane) is marked with an asterisk.

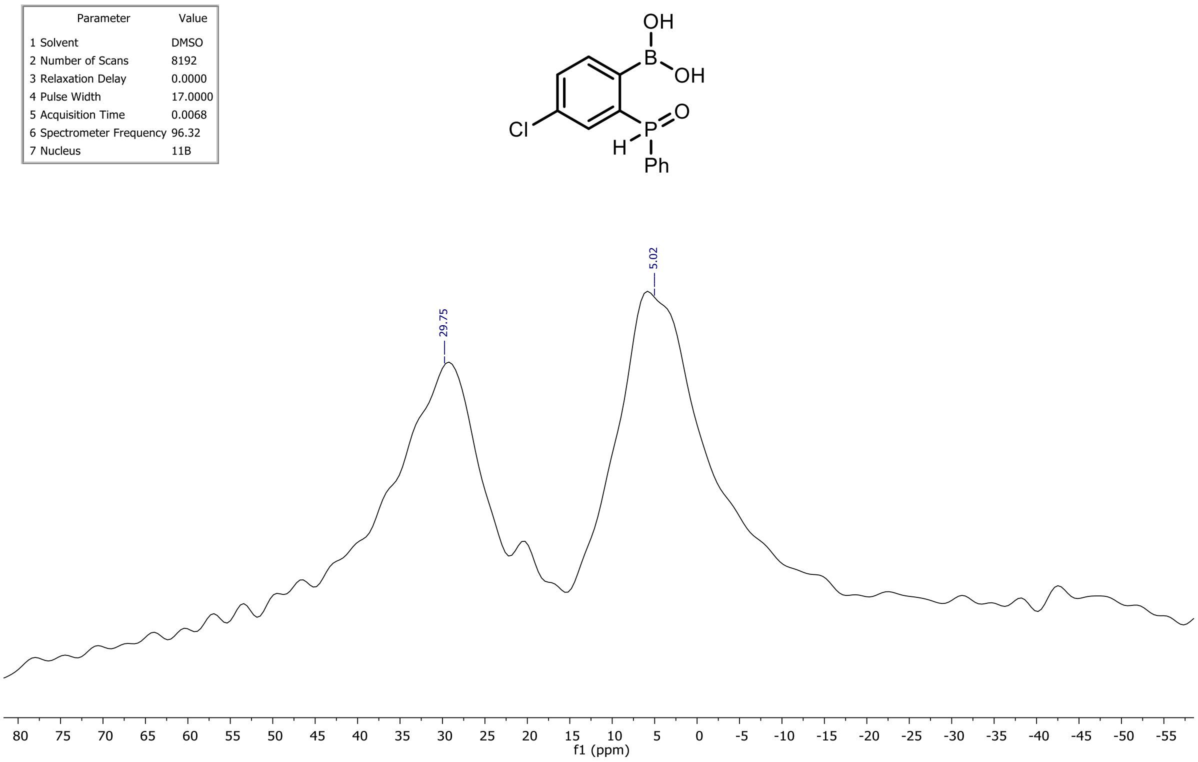

Figure S24. ^{11}B NMR spectrum (96 MHz, DMSO-d_6) of **7**.

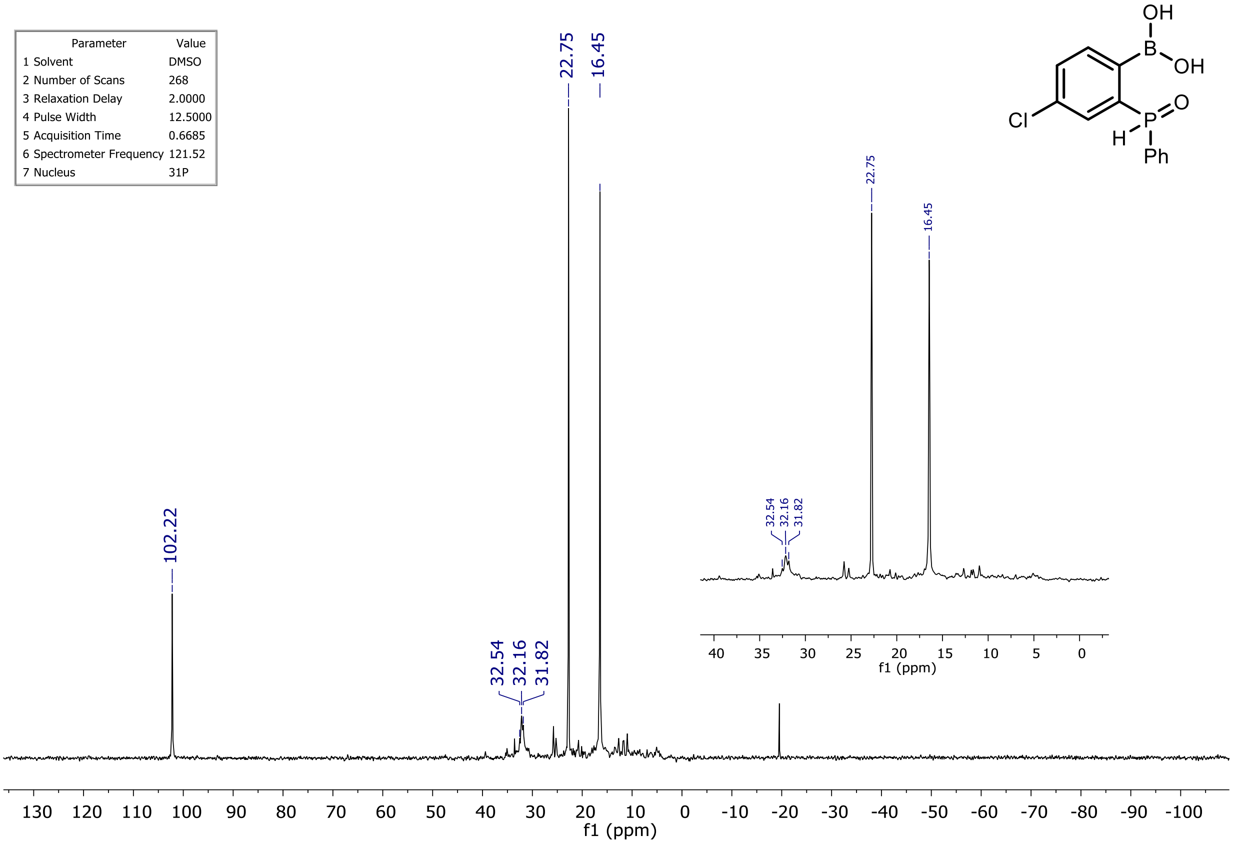

Figure S25. $^{31}P\{^{1}H\}$ NMR spectrum (122 MHz, $CDCl_3$) of **7**.

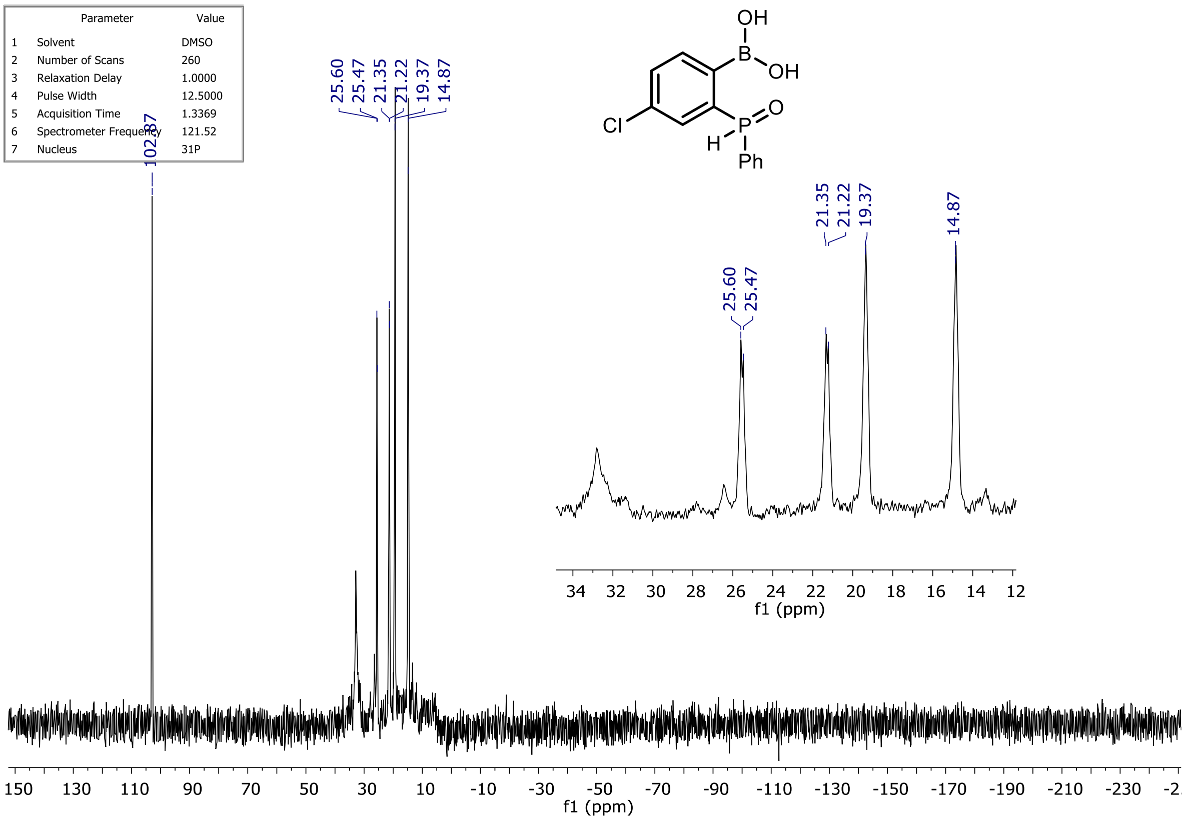

Figure S26. ^{31}P NMR spectrum (122 MHz, DMSO-d_6) of **7**.

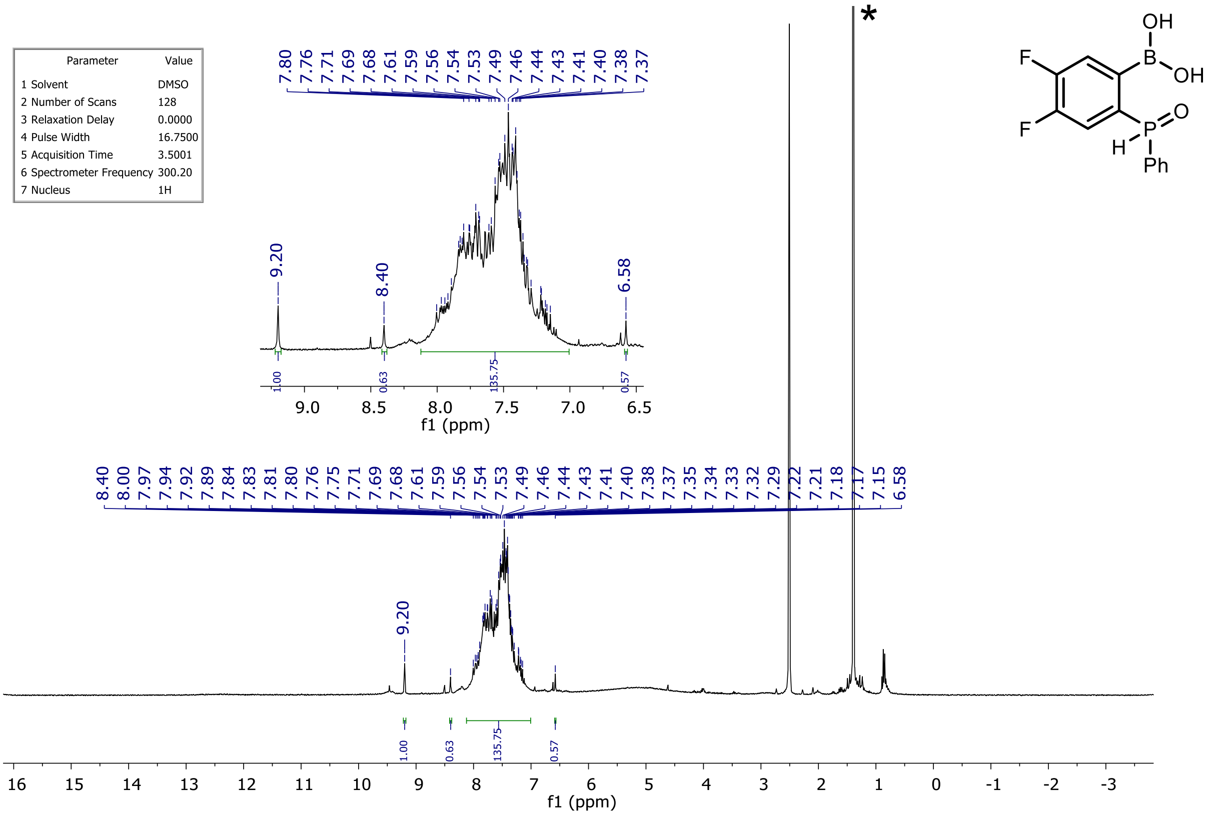

Figure S27. ^{1}H NMR spectrum (300 MHz, DMSO-d_6) of **8**. The signal of a residual solvent (cyclohexane) is marked with an asterisk.

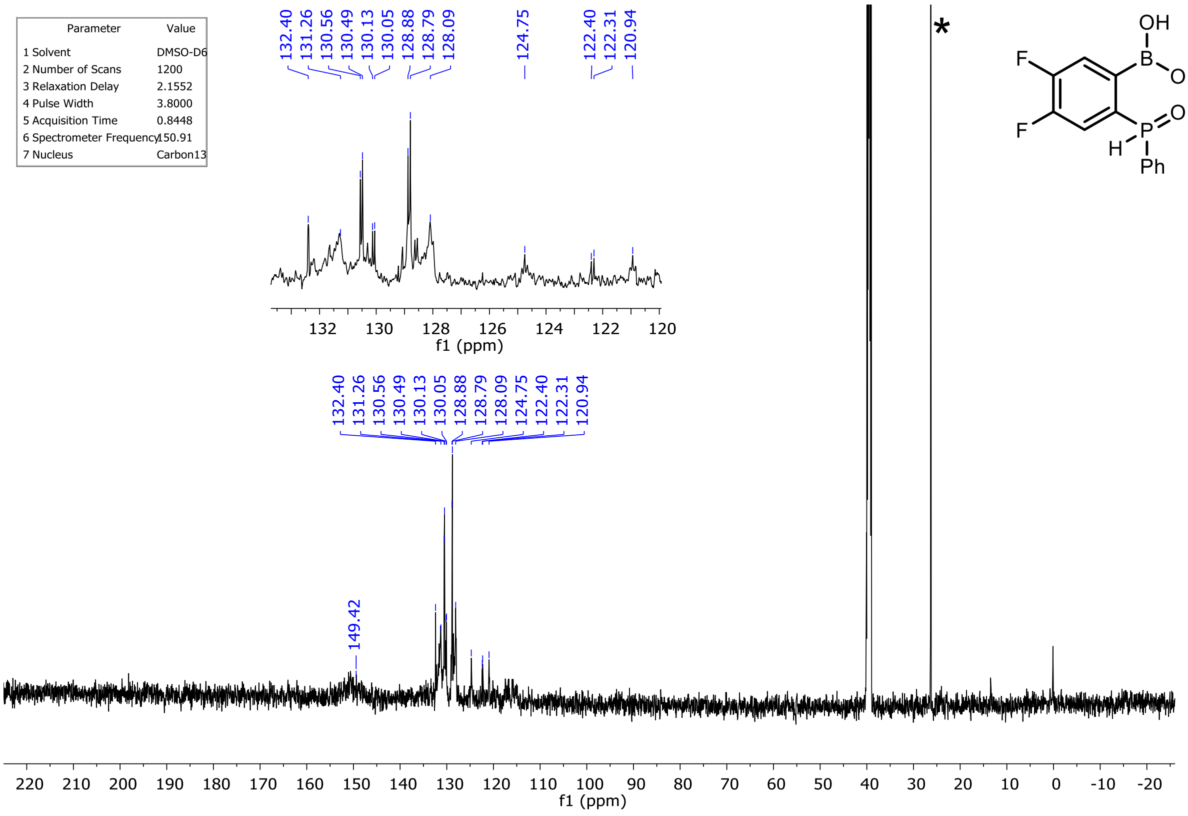

Figure S28. ^{13}C{^{1}H} NMR spectrum (101 MHz, DMSO-d_6) of **8**. The signal of a residual solvent (cyclohexane) is marked with an asterisk.

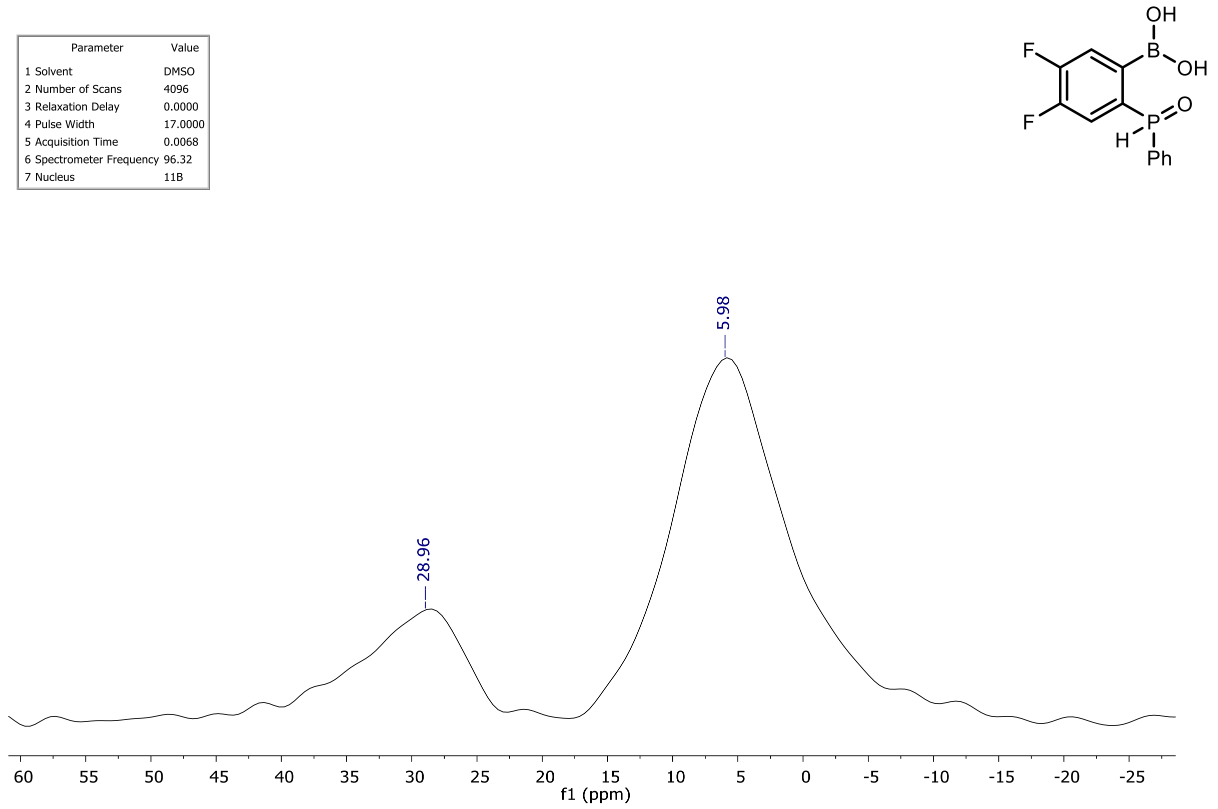

Figure S29. ^{11}B NMR spectrum (96 MHz, DMSO-d_6) of **8**.

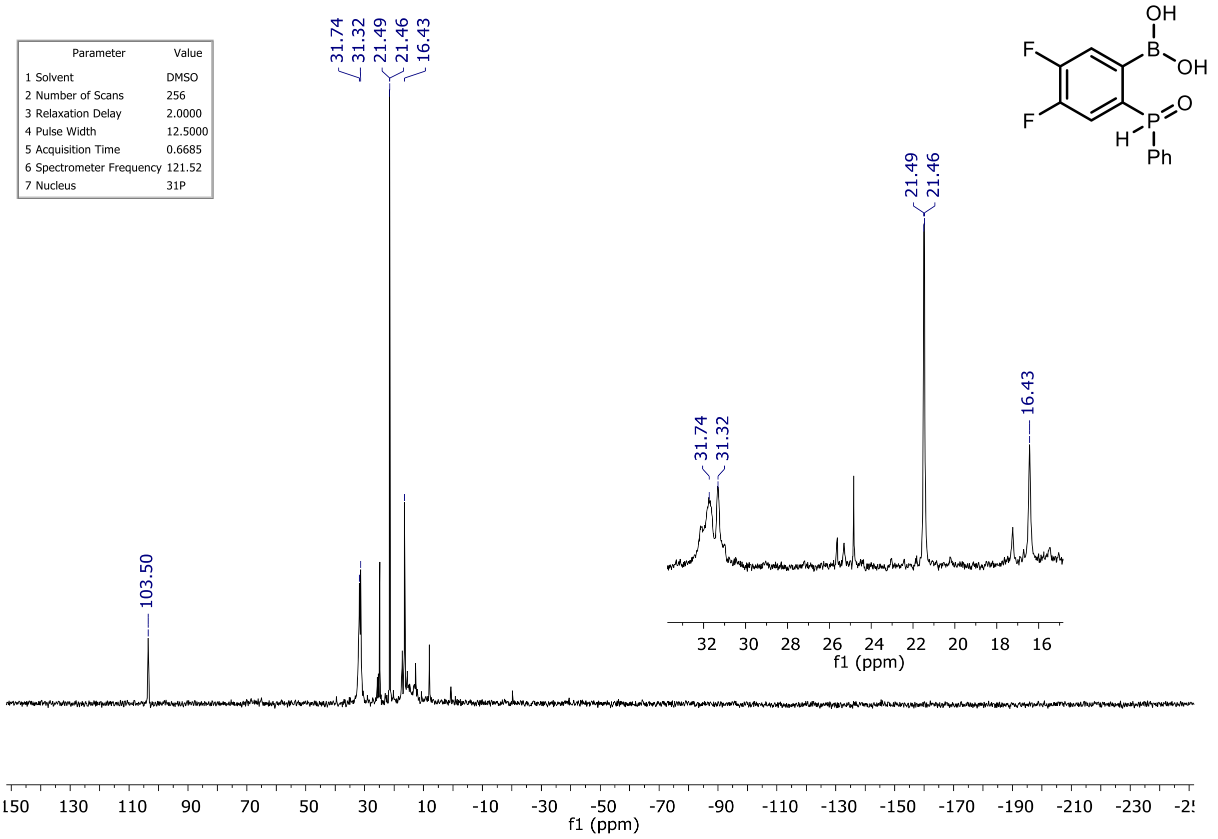

Figure S30. ^{31}P{^{1}H} NMR spectrum (122 MHz, $CDCl_3$) of **8**.

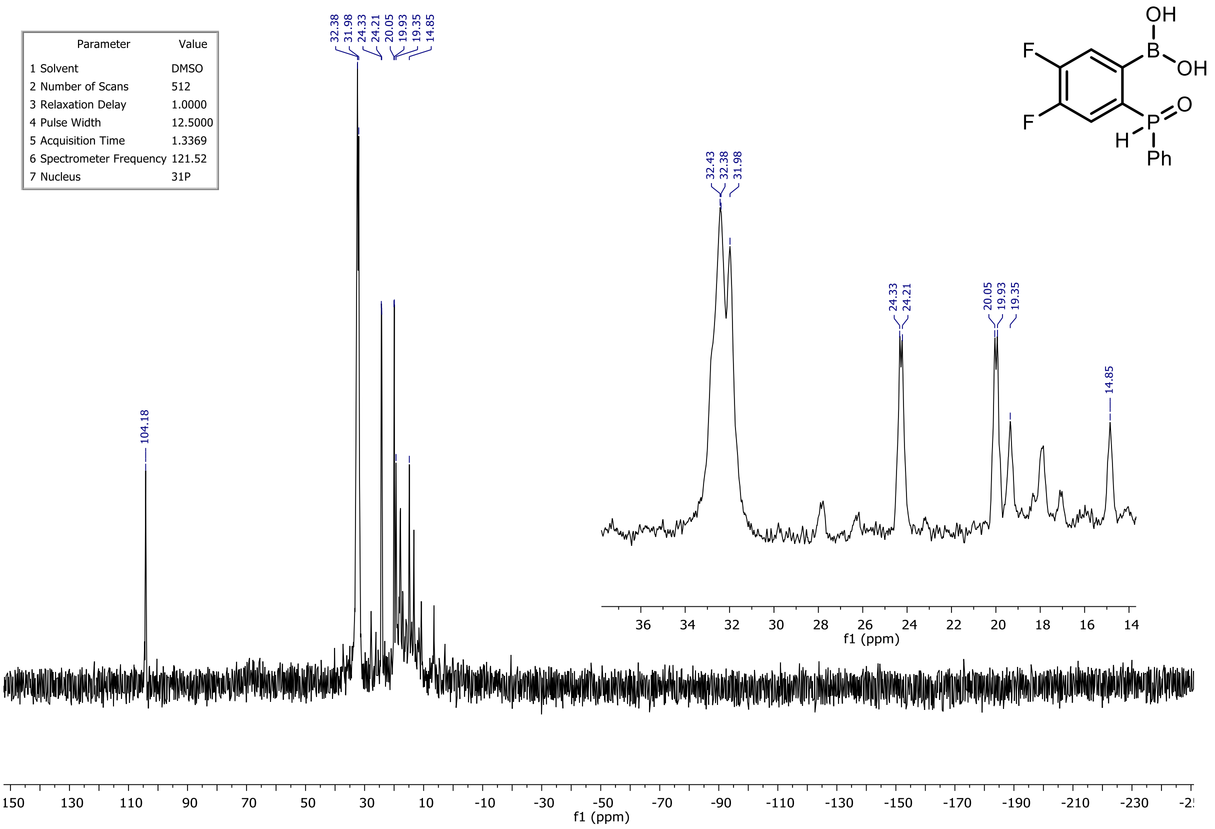

Figure S31. ^{31}P NMR spectrum (122 MHz, DMSO-d_6) of **8**.

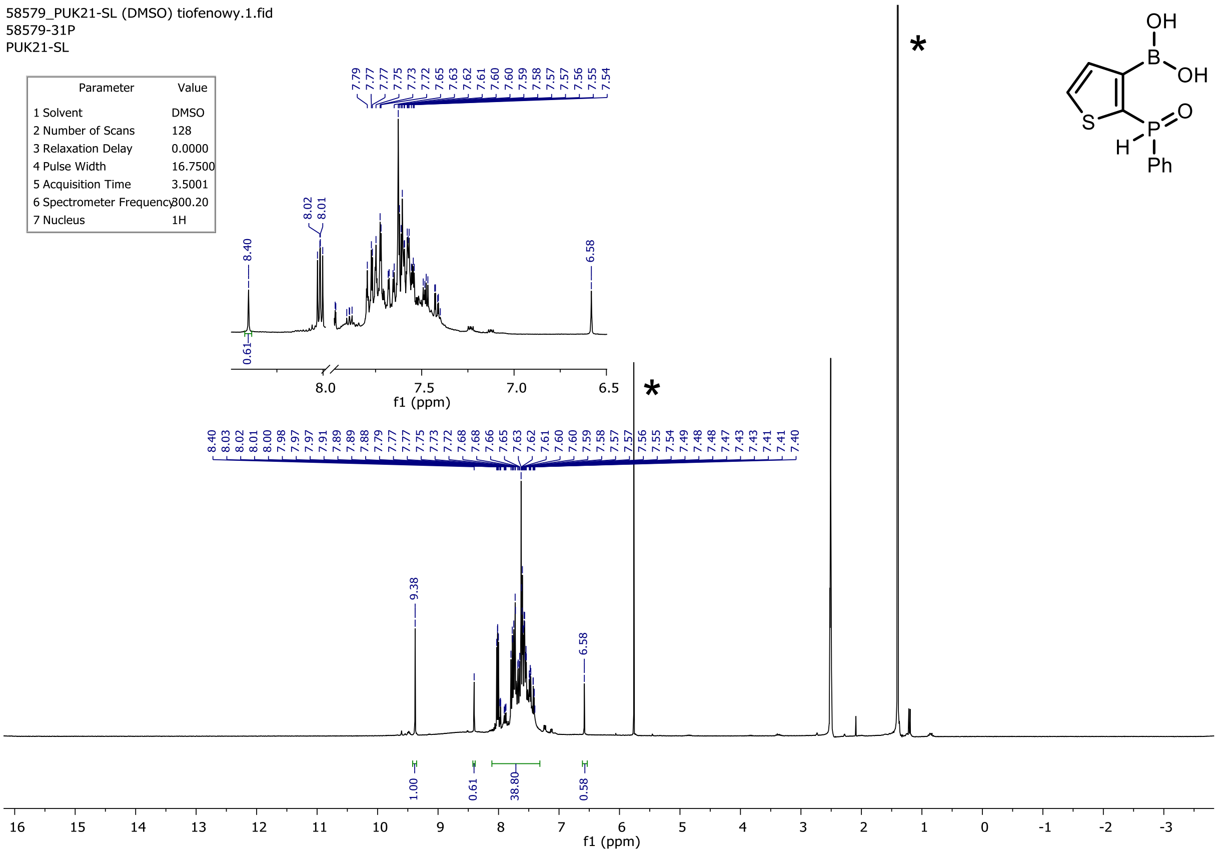

Figure S32. ^{1}H NMR spectrum (300 MHz, DMSO-d_6) of **10**. The signals of residual solvents (DCM and cyclohexane) are marked with asterisks.

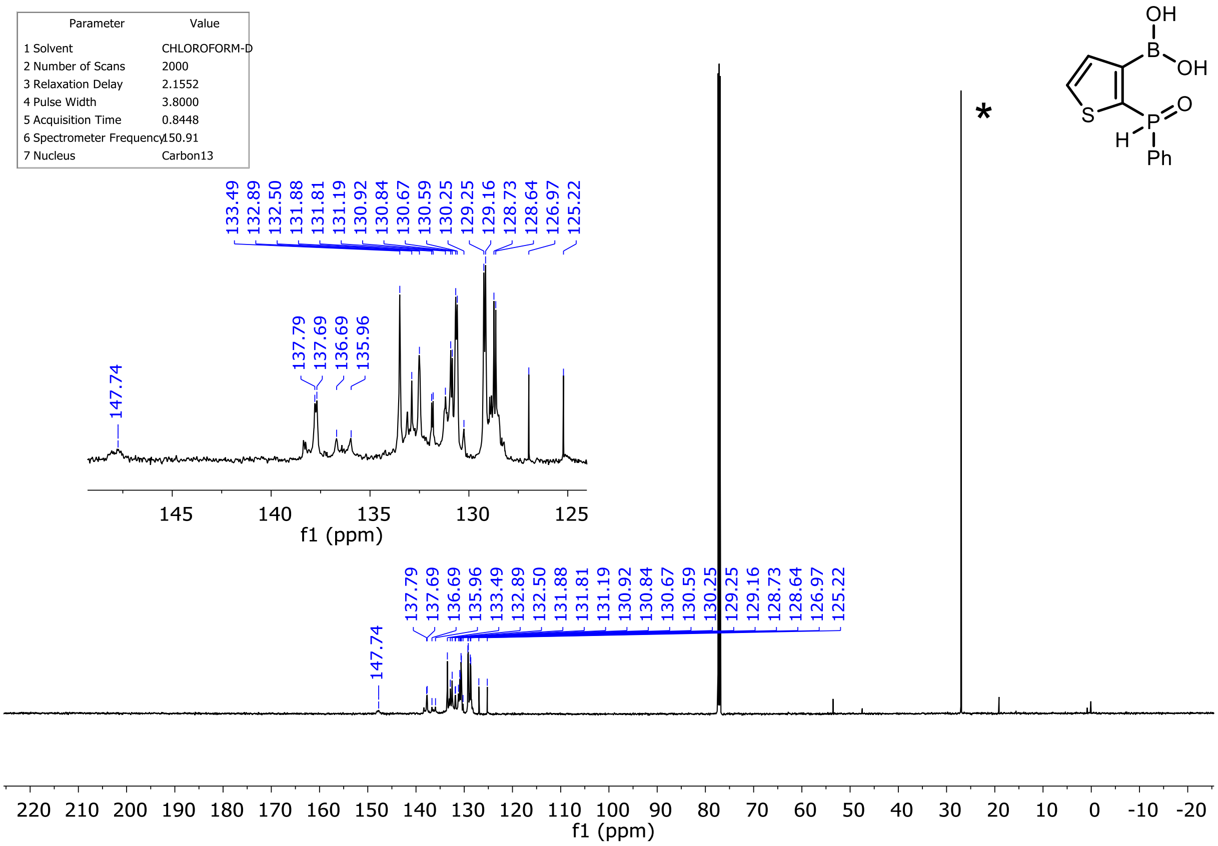

Figure S33. $^{13}C\{^{1}H\}$ NMR spectrum (101 MHz, $CDCl_3$) of **10**. The signal of a residual solvent (cyclohexane) is marked with an asterisk.

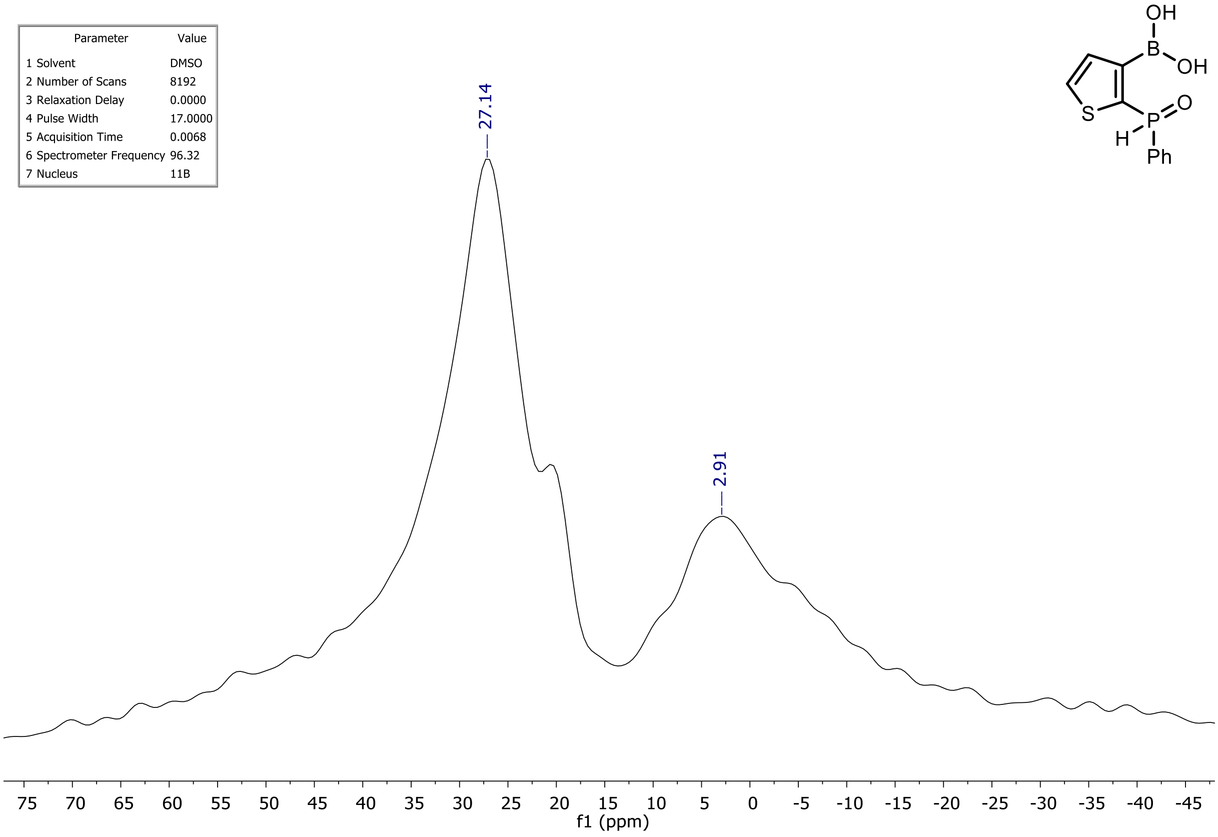

Figure S34. ^{11}B NMR spectrum (96 MHz, DMSO-d_6) of **10**.

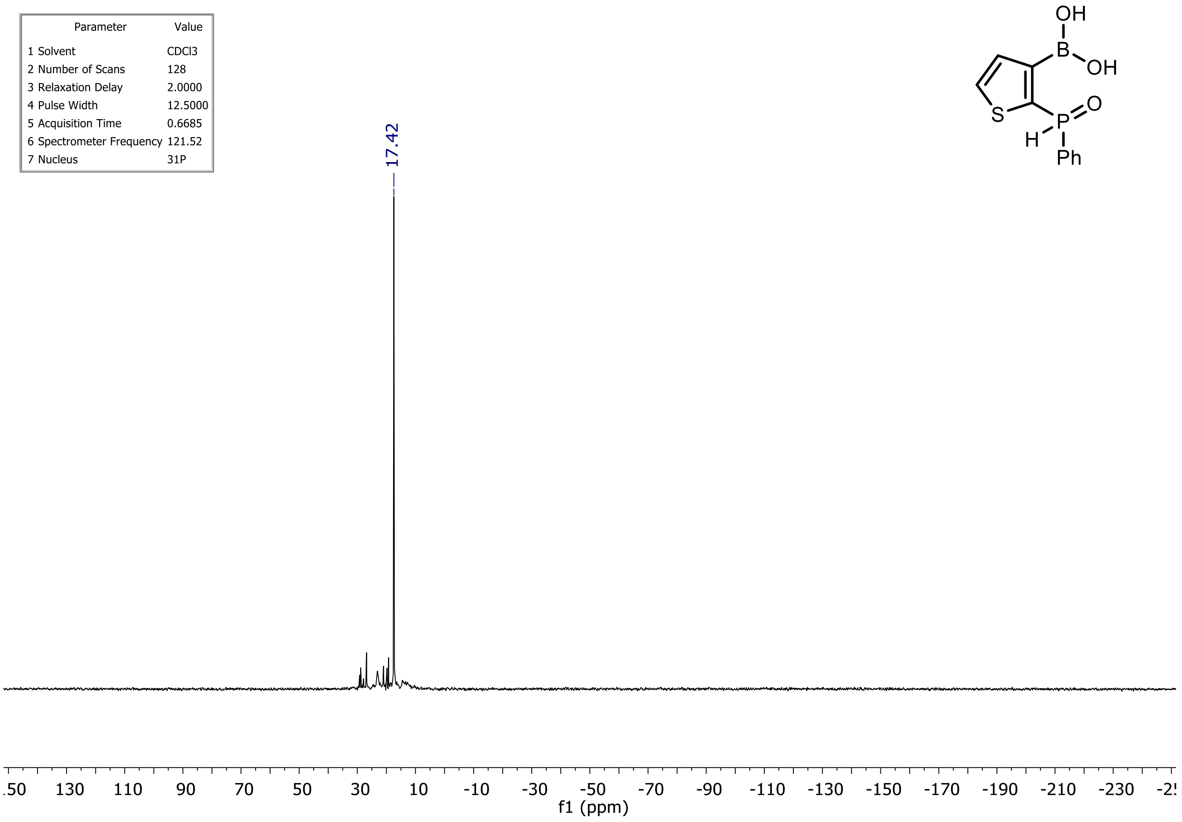

Figure S35. ^{31}P{^{1}H} NMR spectrum (122 MHz, CDCl$_3$) of **10**.

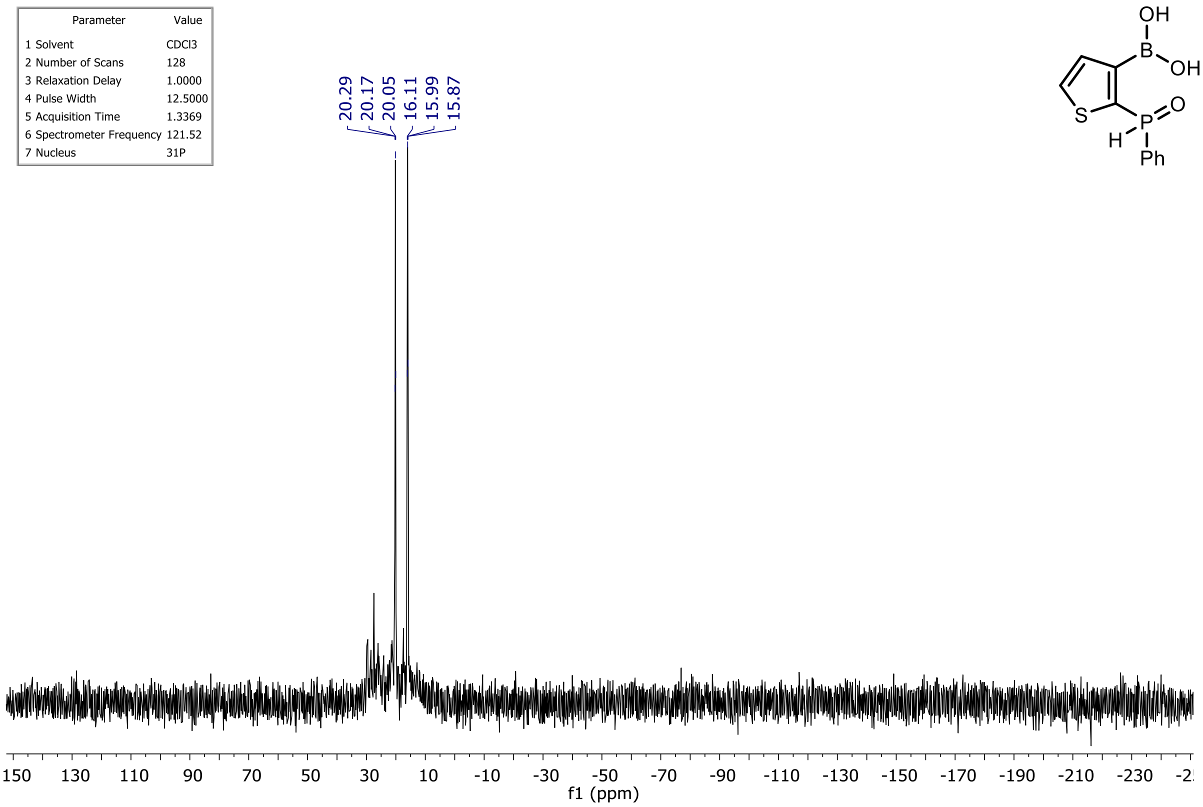

Figure S36. ^{31}P NMR spectrum (122 MHz, DMSO-d_6) of **10**.

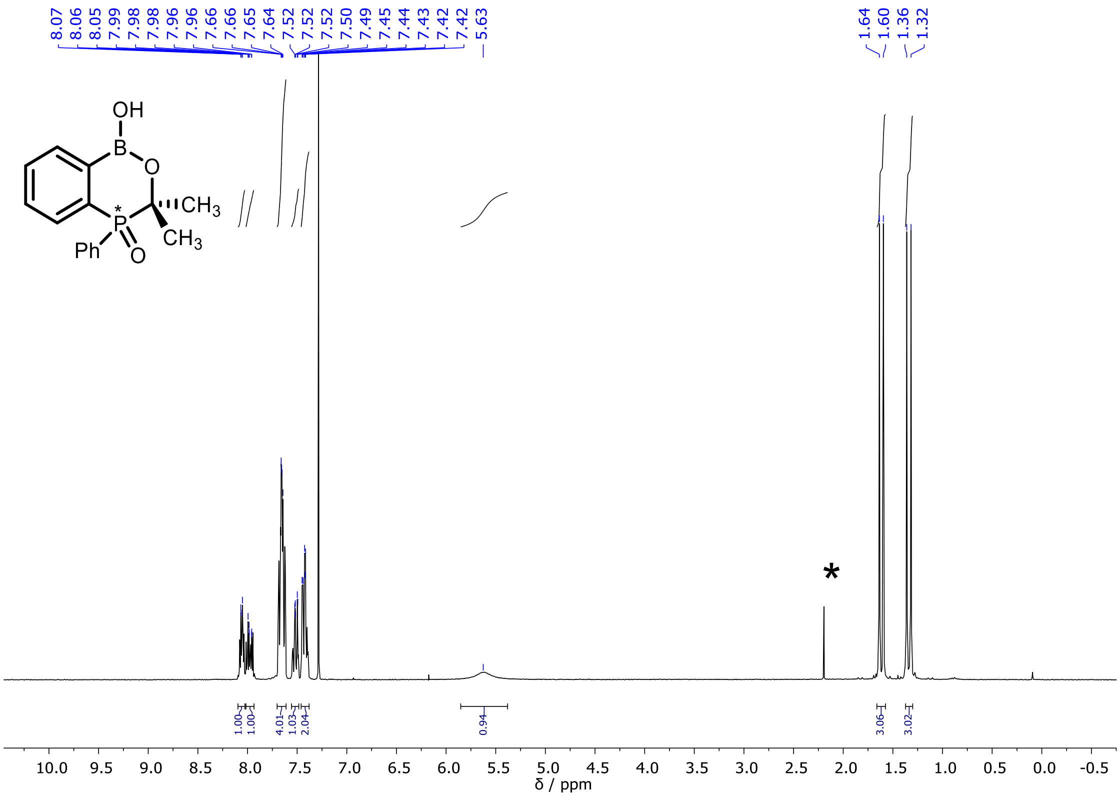

Figure S37. ^{1}H NMR spectrum (300 MHz, $CDCl_3$) of **11**. The signal of a residual solvent (acetone) is marked with an asterisk.

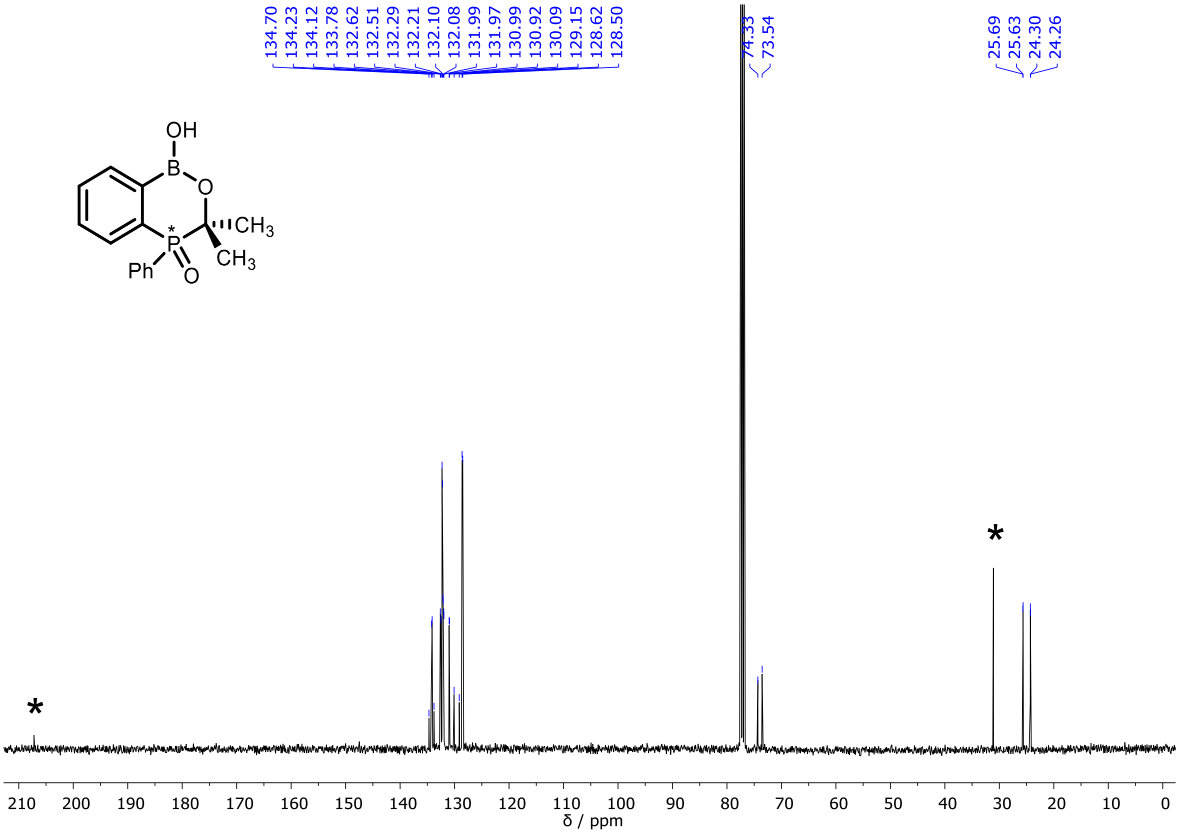

Figure S38. $^{13}C\{^1H\}$ NMR spectrum (101 MHz, $CDCl_3$) of **11**. The signals of a residual solvent (acetone) are marked with asterisks.

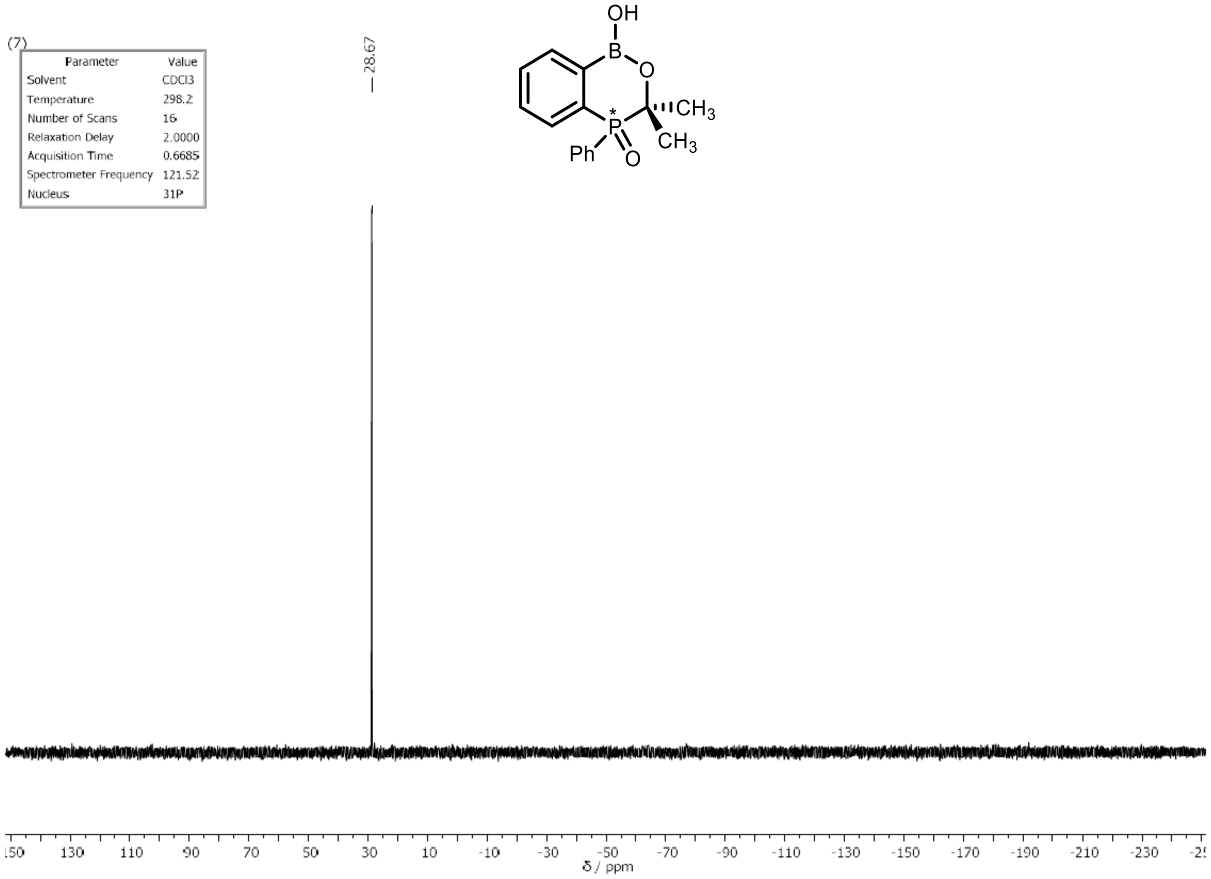

Figure S39. $^{31}P\{^1H\}$ NMR spectrum (122 MHz, $CDCl_3$) of **11**.

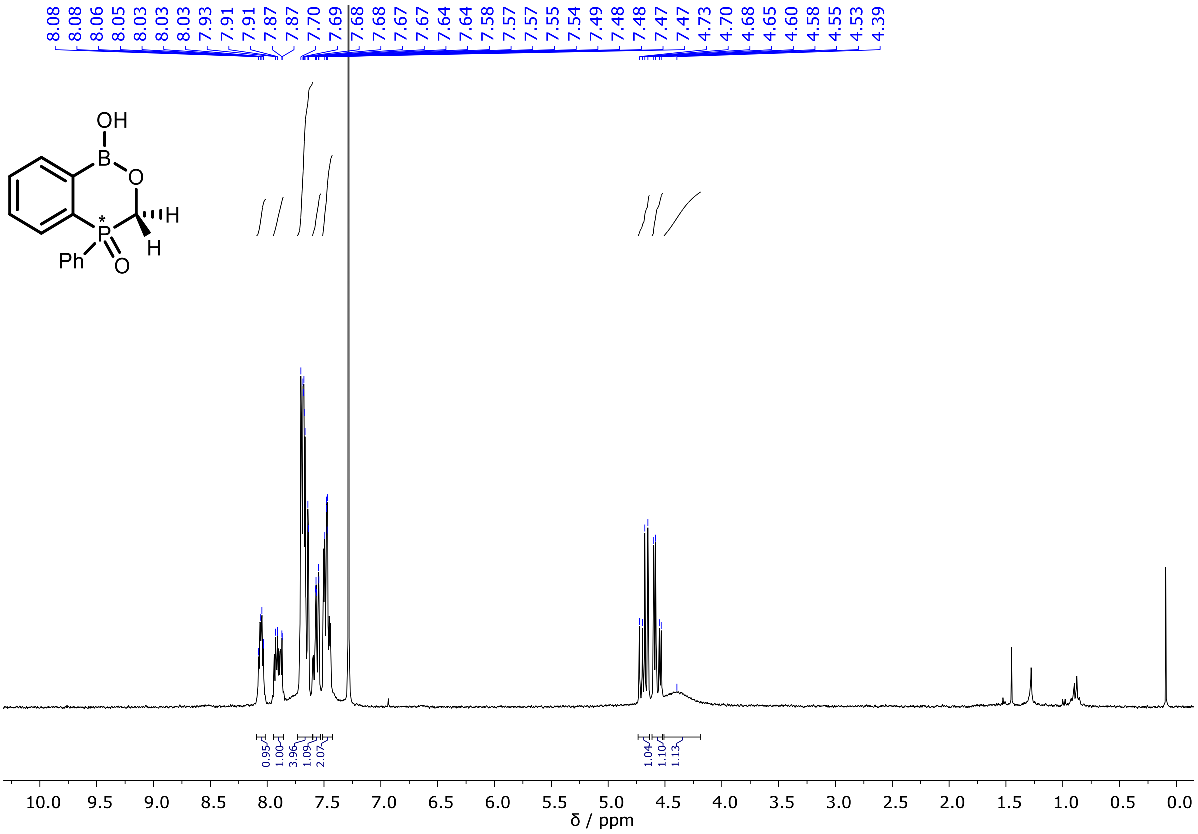

Figure S40. ^{1}H NMR spectrum (300 MHz, $CDCl_3$) of **12**.

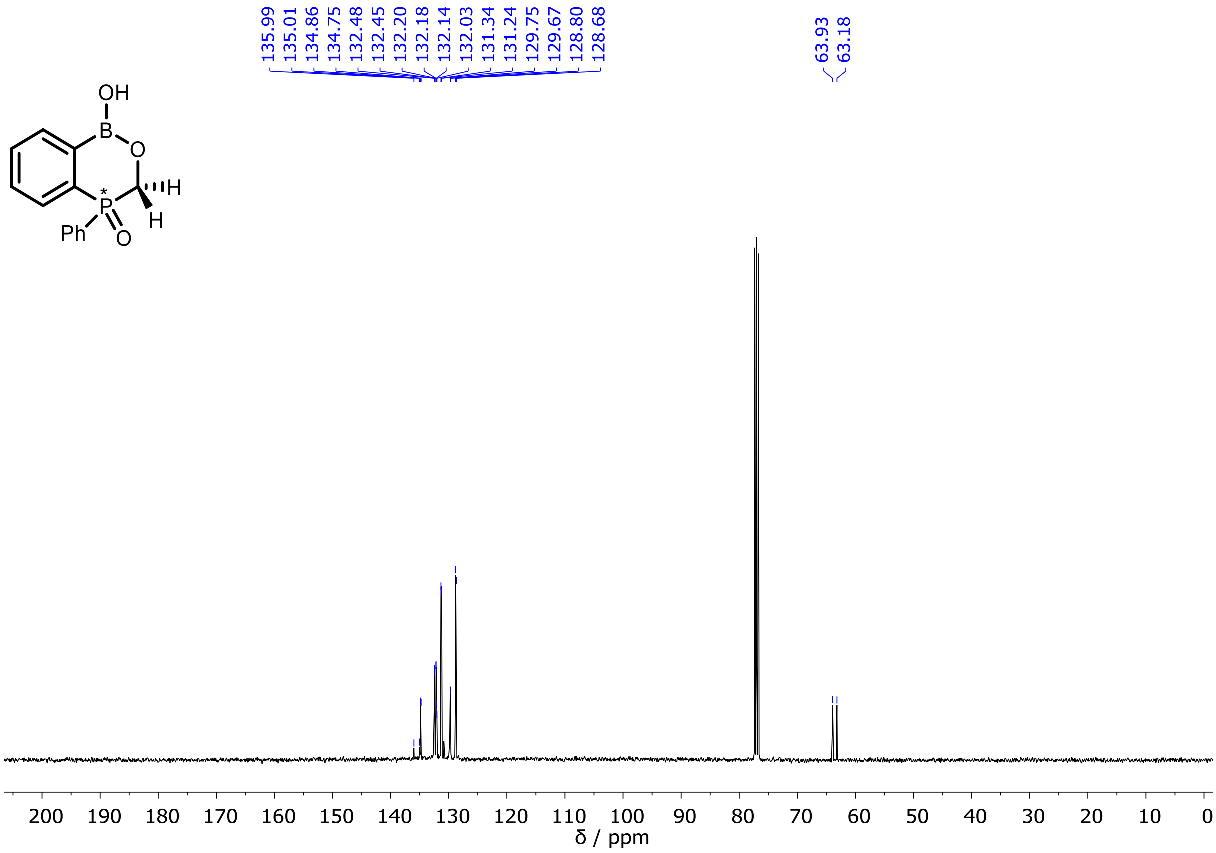

Figure S41. $^{13}C\{^{1}H\}$ NMR spectrum (101 MHz, $CDCl_3$) of **12**.

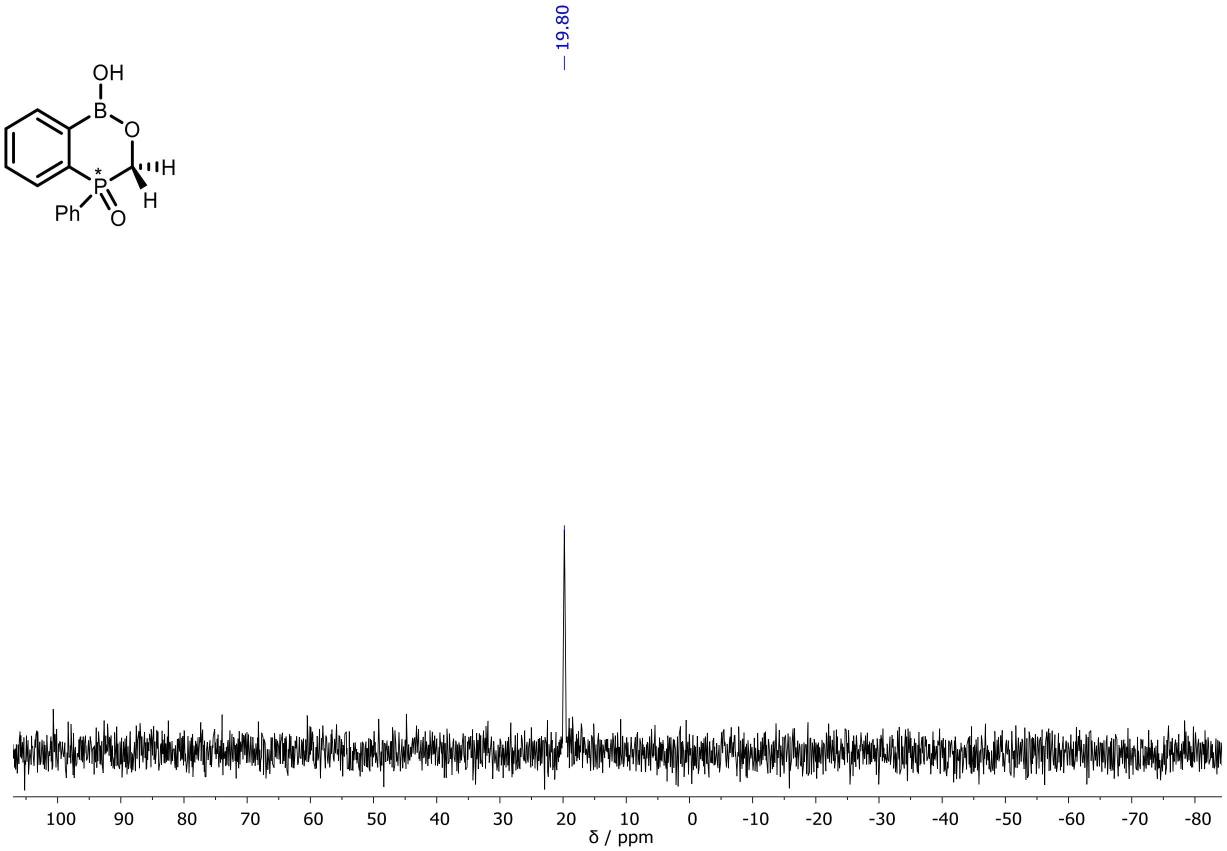

Figure S42. $^{31}P\{^{1}H\}$ NMR spectrum (122 MHz, $CDCl_3$) of **12**.

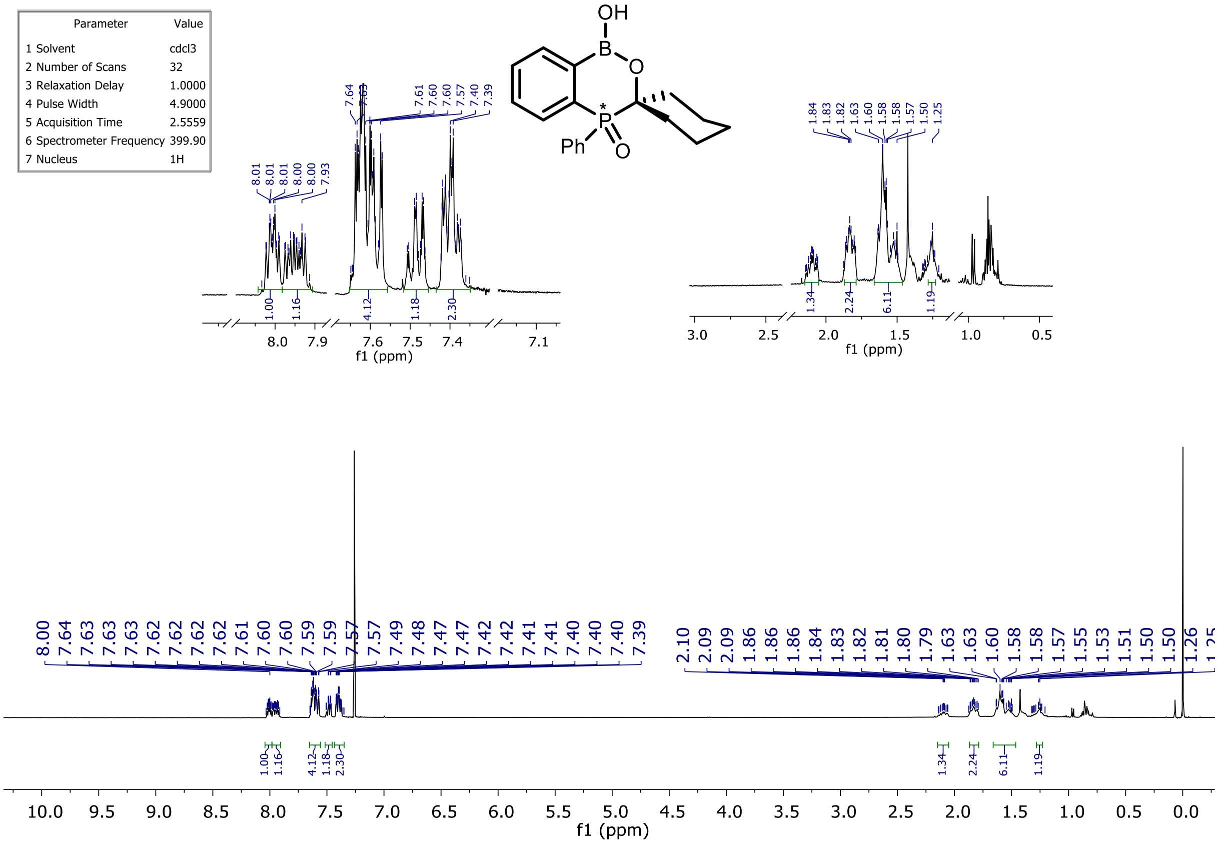

Figure S43. ^{1}H NMR spectrum (400 MHz, $CDCl_3$) of **13**.

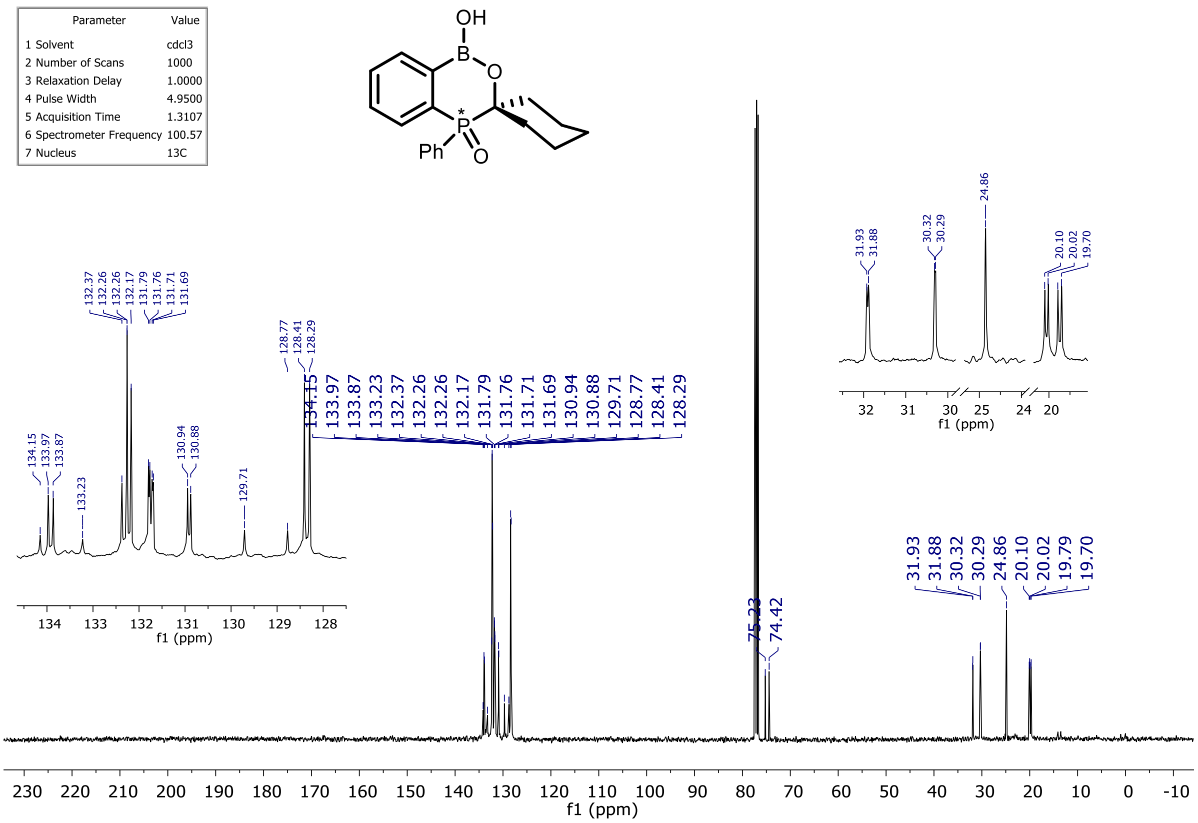

Figure S44. $^{13}C\{^{1}H\}$ NMR spectrum (101 MHz, $CDCl_3$) of **13**.

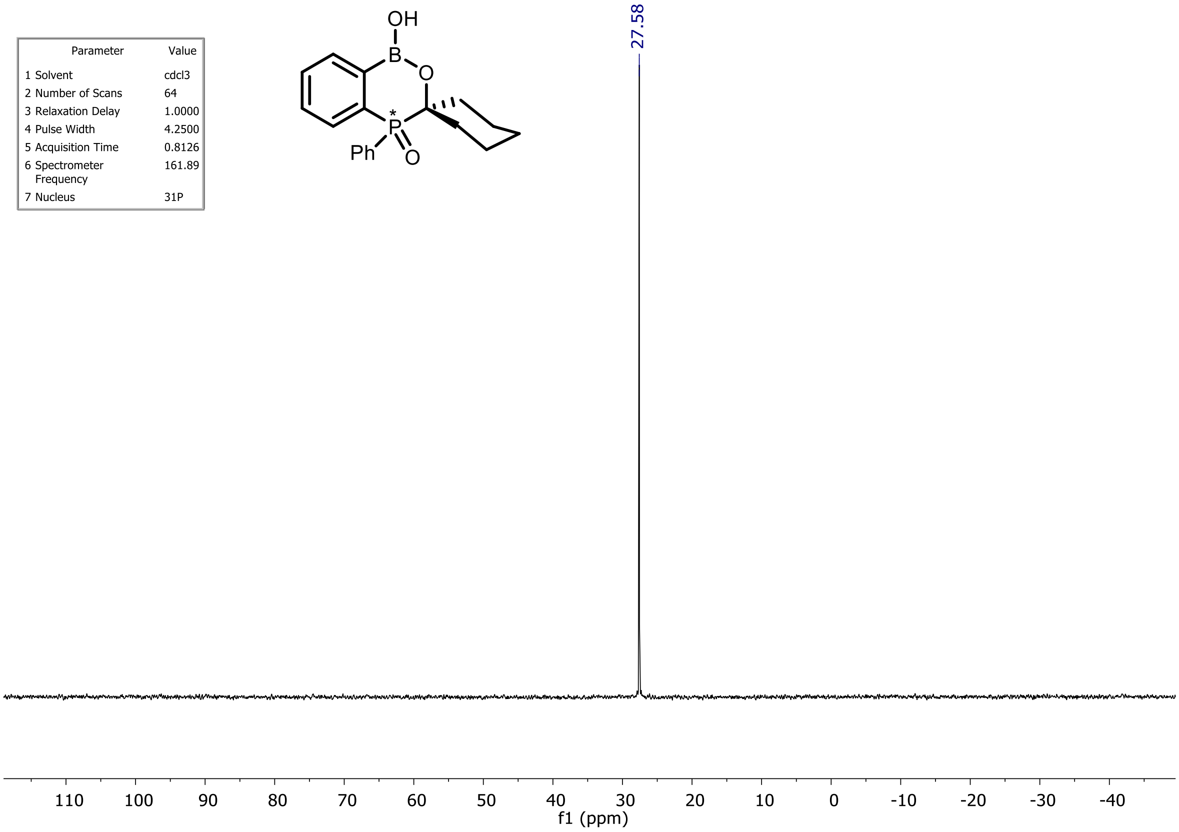

Figure S45. $^{31}P\{^{1}H\}$ NMR spectrum (162 MHz, $CDCl_3$) of **13**.

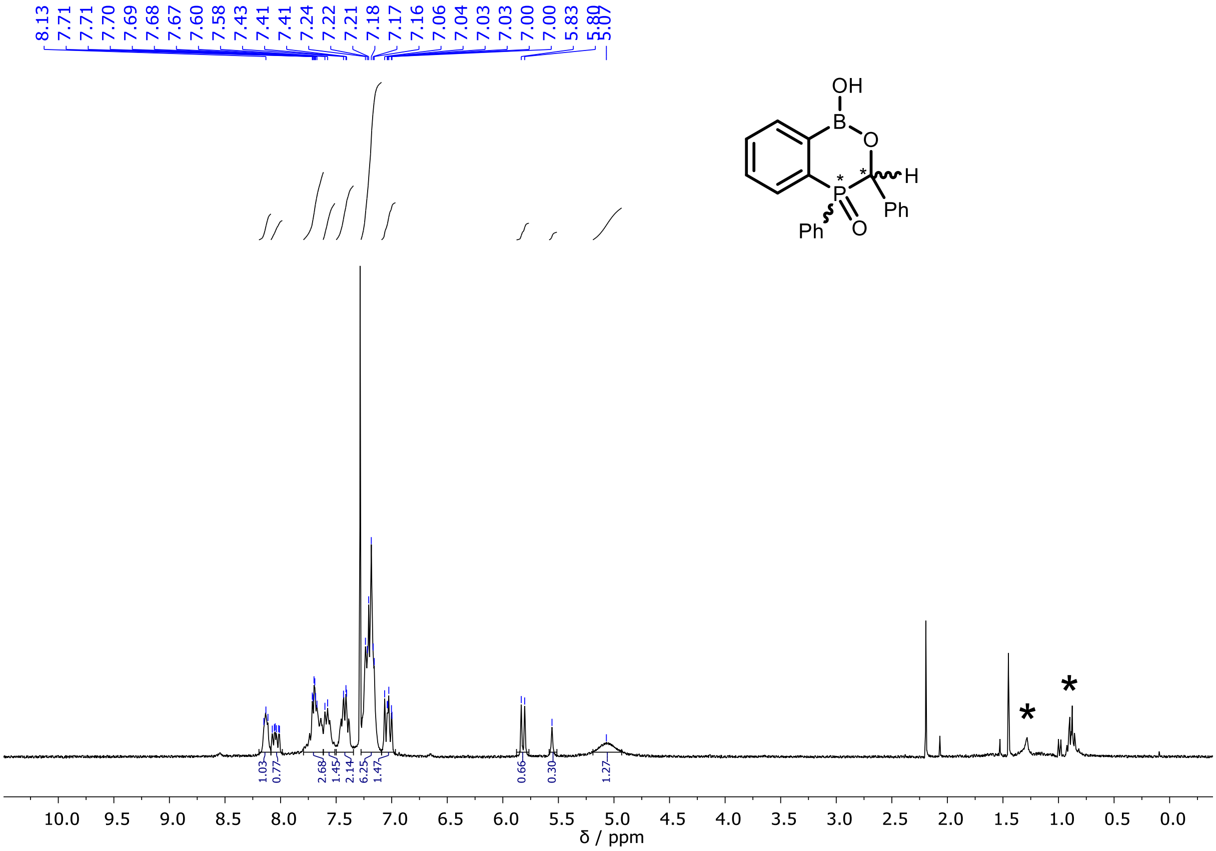

Figure S46. ^{1}H NMR spectrum (300 MHz, $CDCl_3$) of **14**. The signals of a residual solvent (hexane) is marked with asterisks.

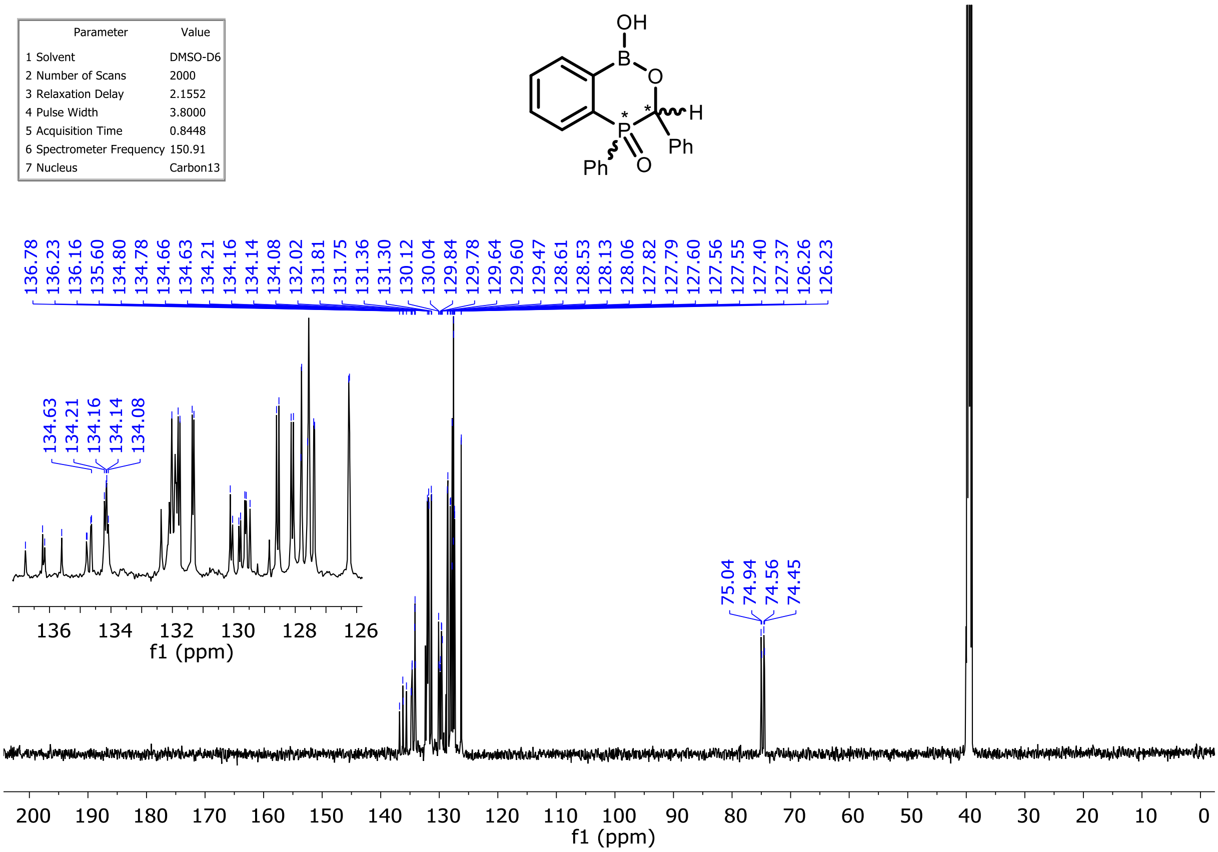

Figure S48. $^{13}C\{^{1}H\}$ NMR spectrum (101 MHz, DMSO-d_6) of **14**.

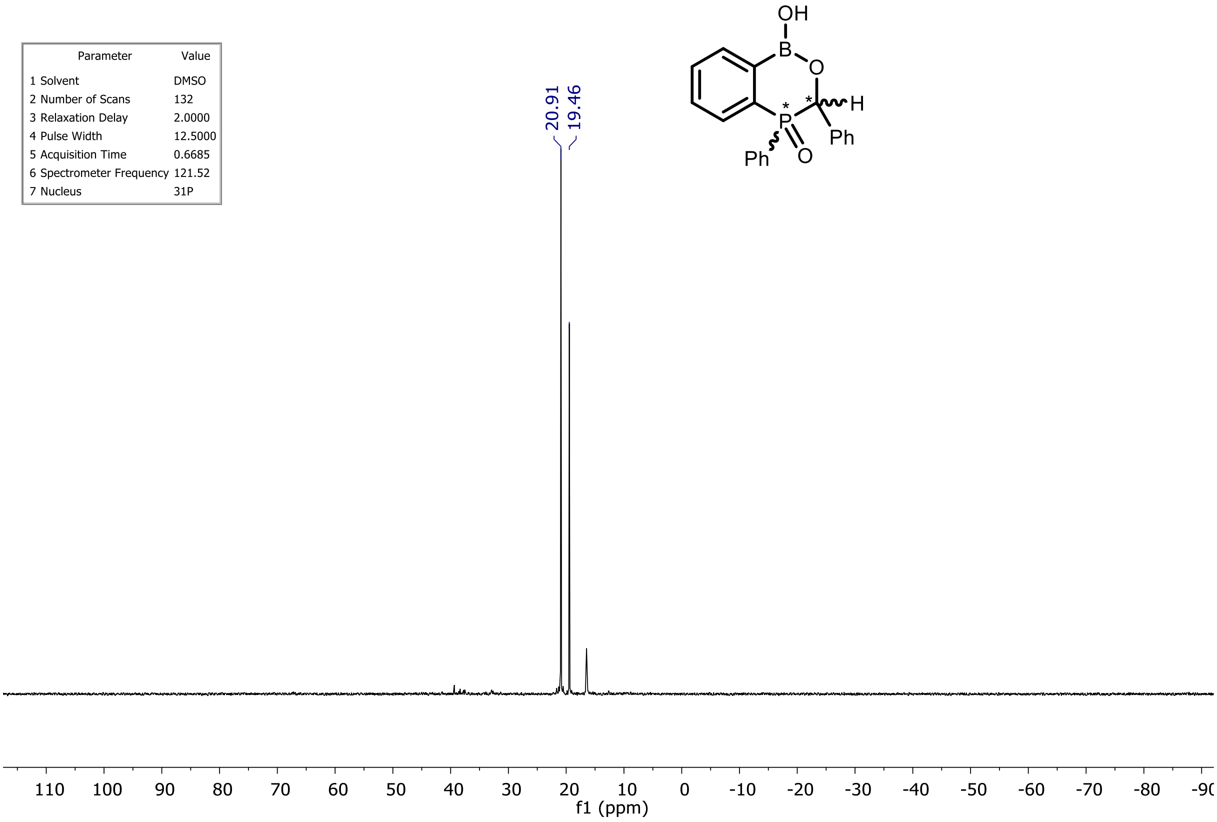

Figure S47. $^{31}P\{^{1}H\}$ NMR spectrum (122 MHz, $CDCl_3$) of **14**.

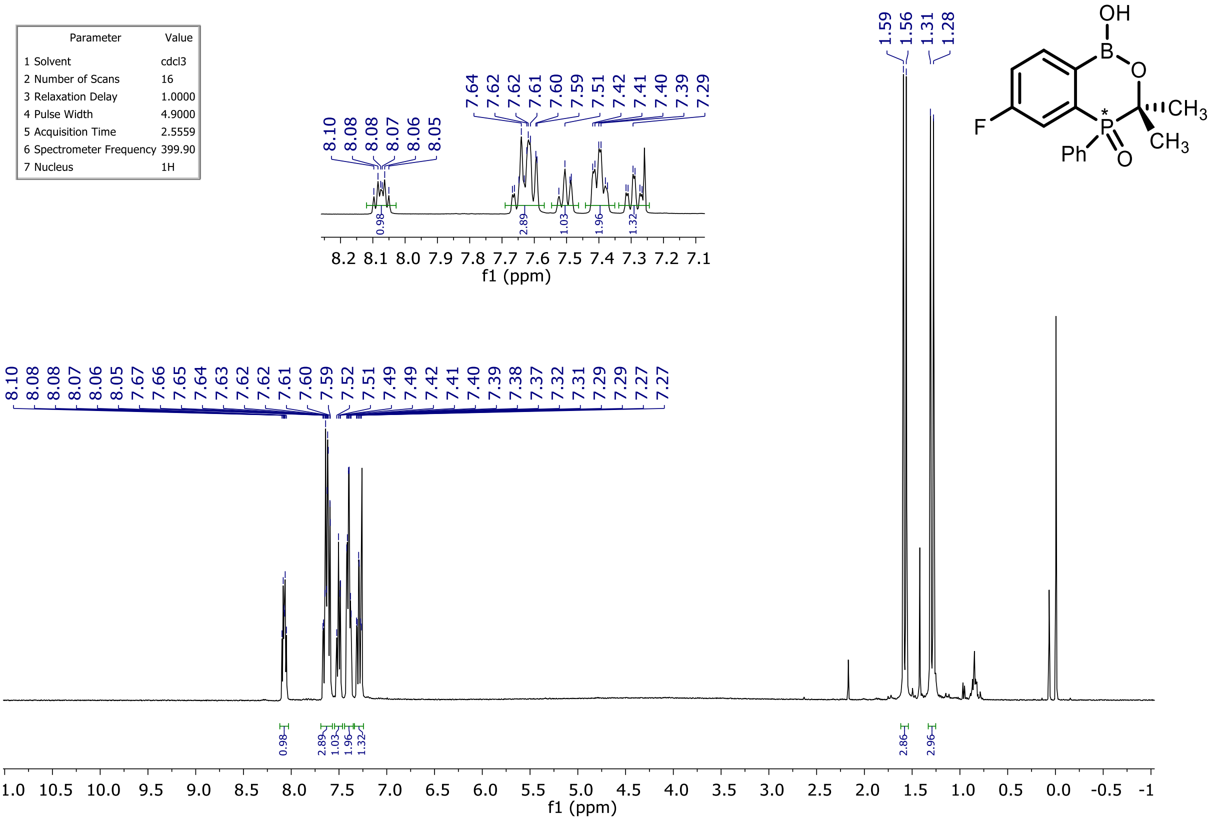

Figure S49. ^{1}H NMR spectrum (400 MHz, $CDCl_3$) of **15**.

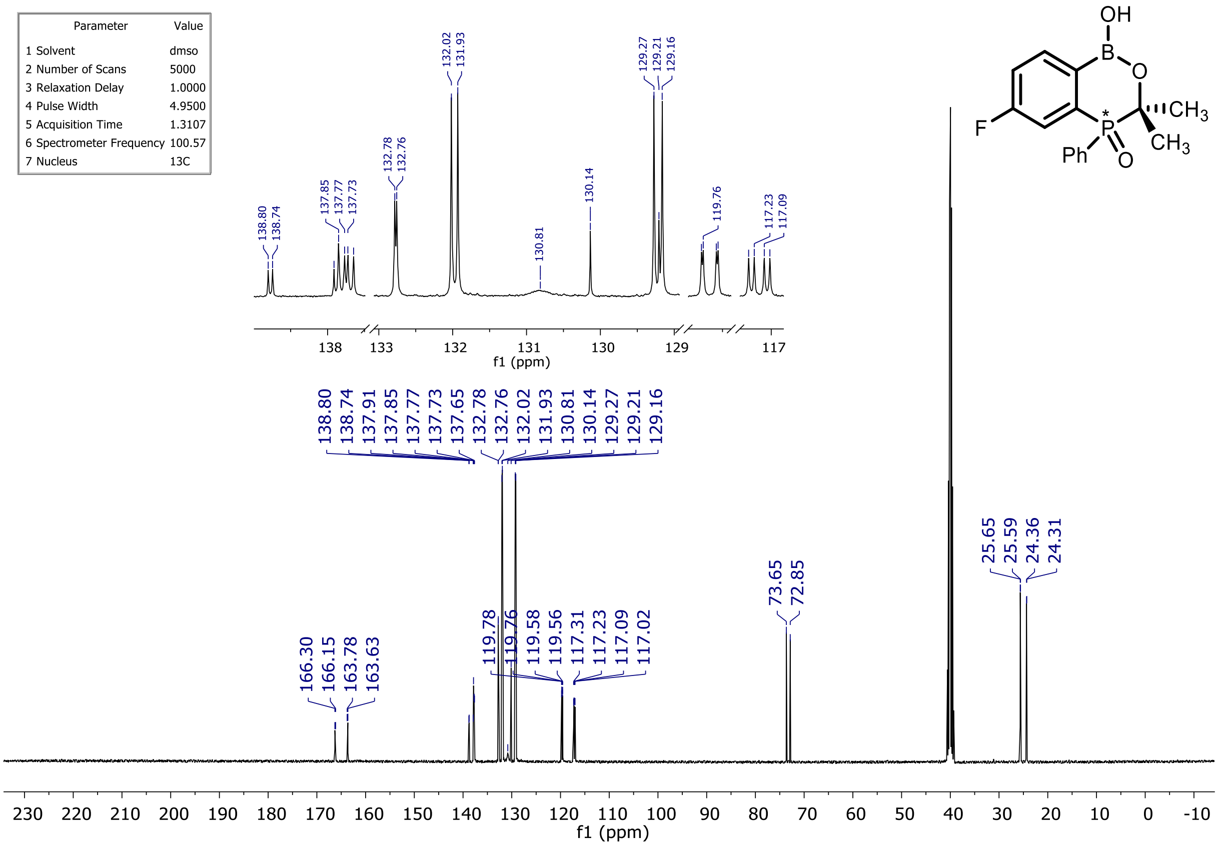

Figure S50. $^{13}C\{^1H\}$ NMR spectrum (101 MHz, $CDCl_3$) of **15**.

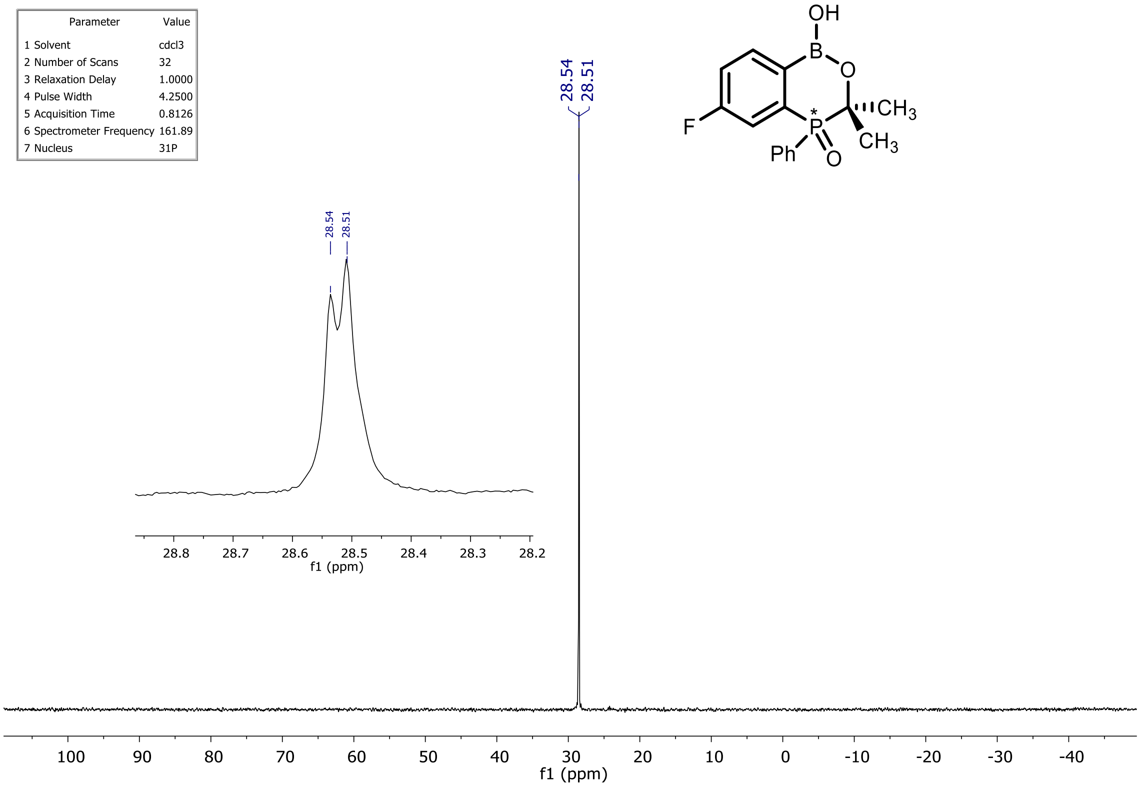

Figure S51. $^{31}P\{^1H\}$ NMR spectrum (162 MHz, $CDCl_3$) of **15**.

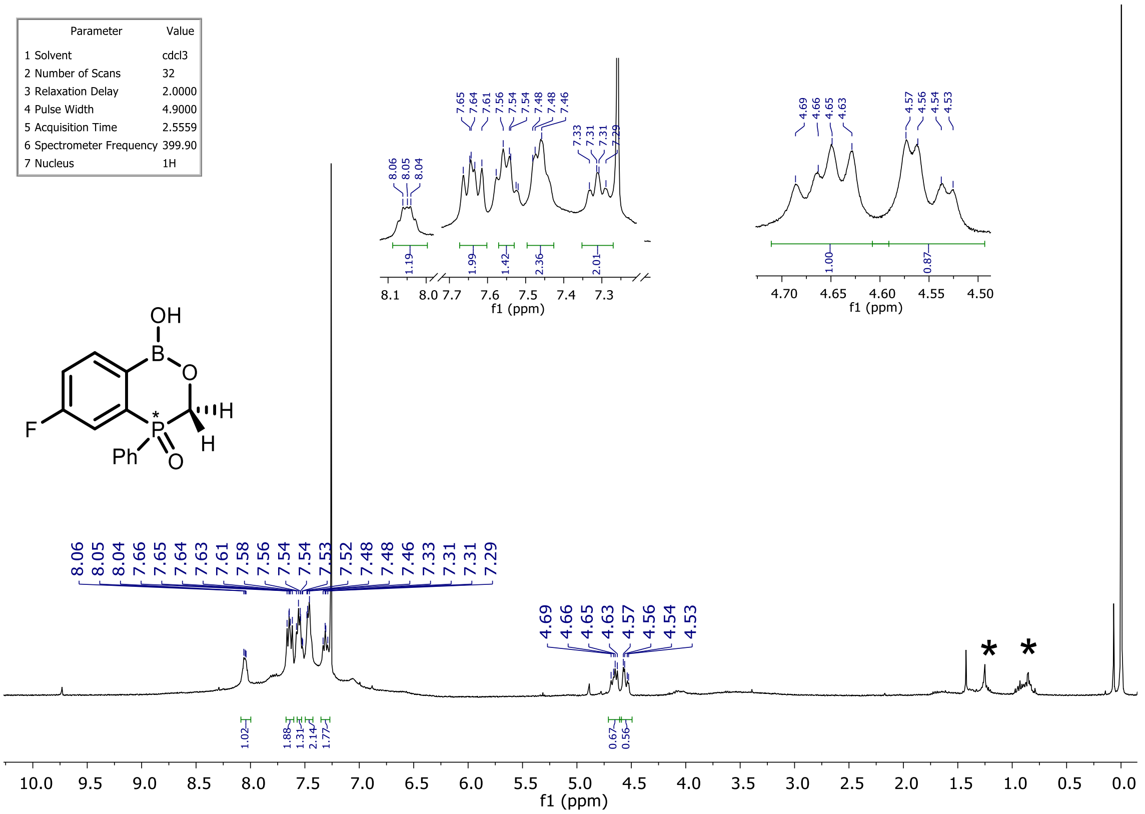

Figure S52. ^{1}H NMR spectrum (400 MHz, $CDCl_3$) of **16**. The signals of a residual solvent (hexane) is marked with asterisks.

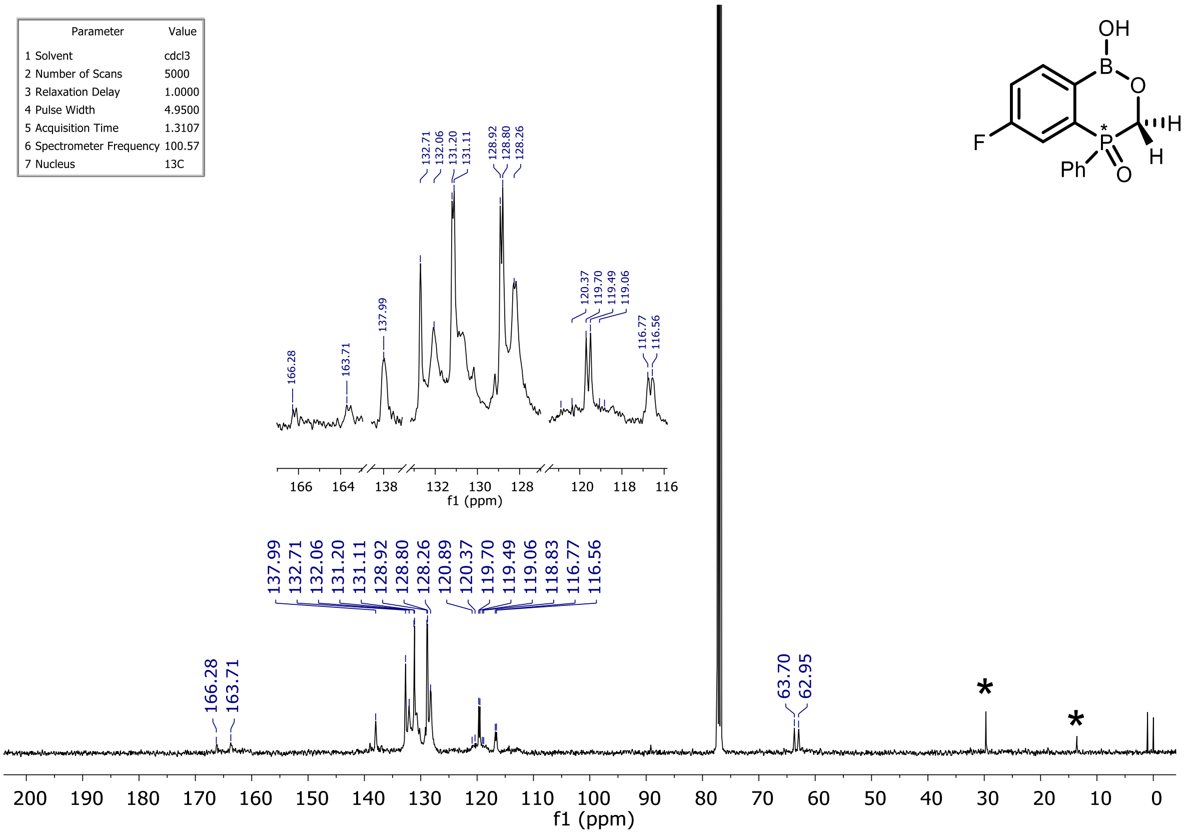

Figure S53. $^{13}C\{^{1}H\}$ NMR spectrum (101 MHz, $CDCl_3$) of **16**. The signals of a residual solvent (hexane) is marked with asterisks.

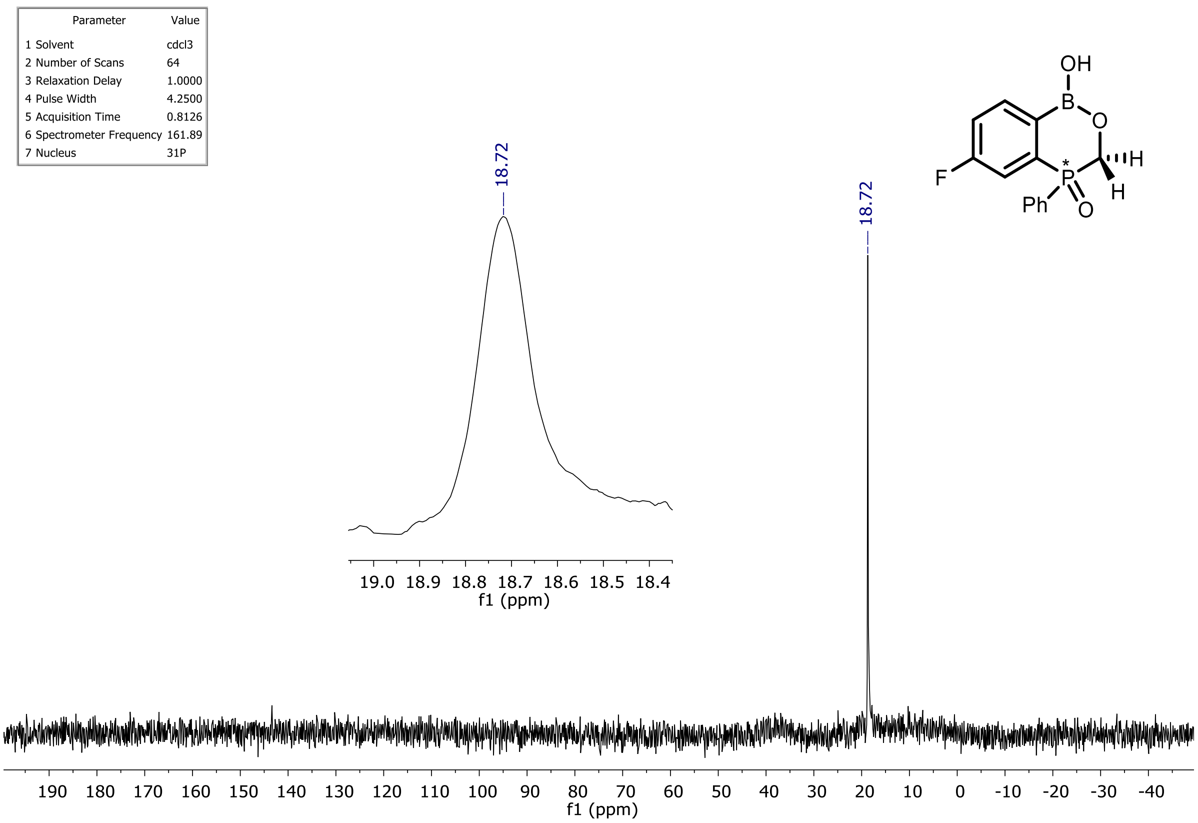

Figure S54. $^{31}P\{^1H\}$ NMR spectrum (162 MHz, $CDCl_3$) of **16**.

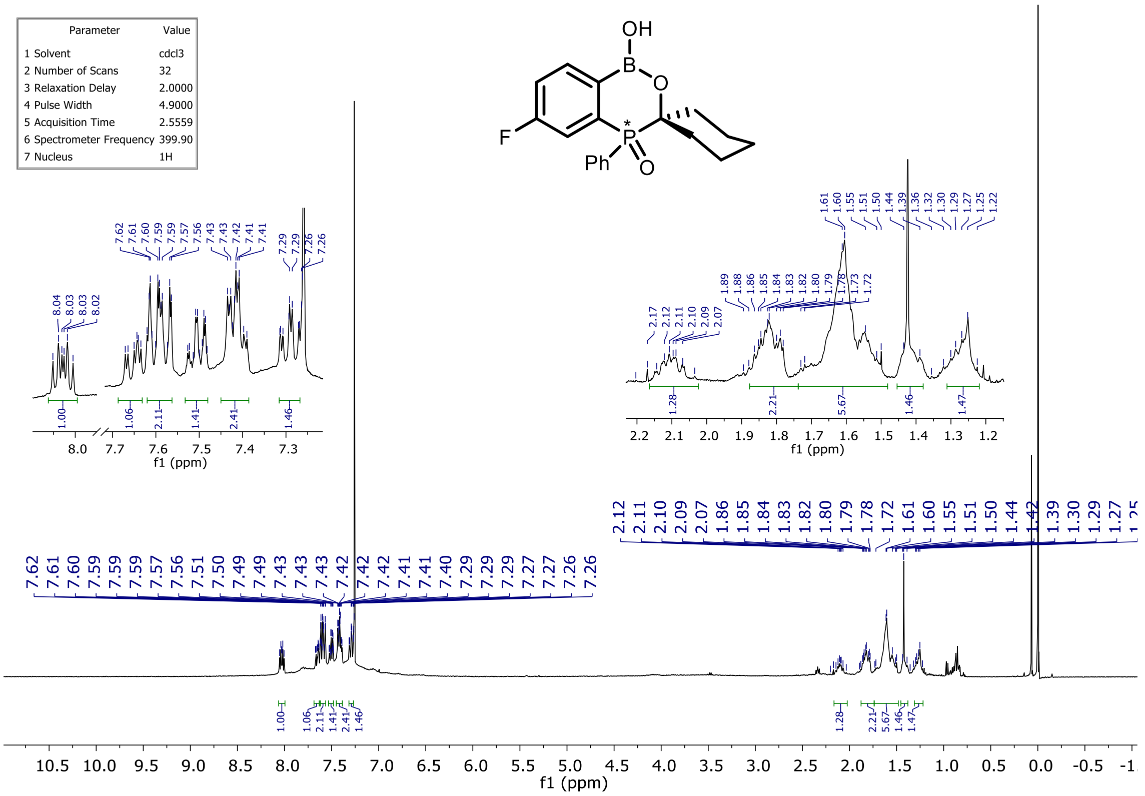

Figure S55. ^1H NMR spectrum (400 MHz, $CDCl_3$) of **17**.

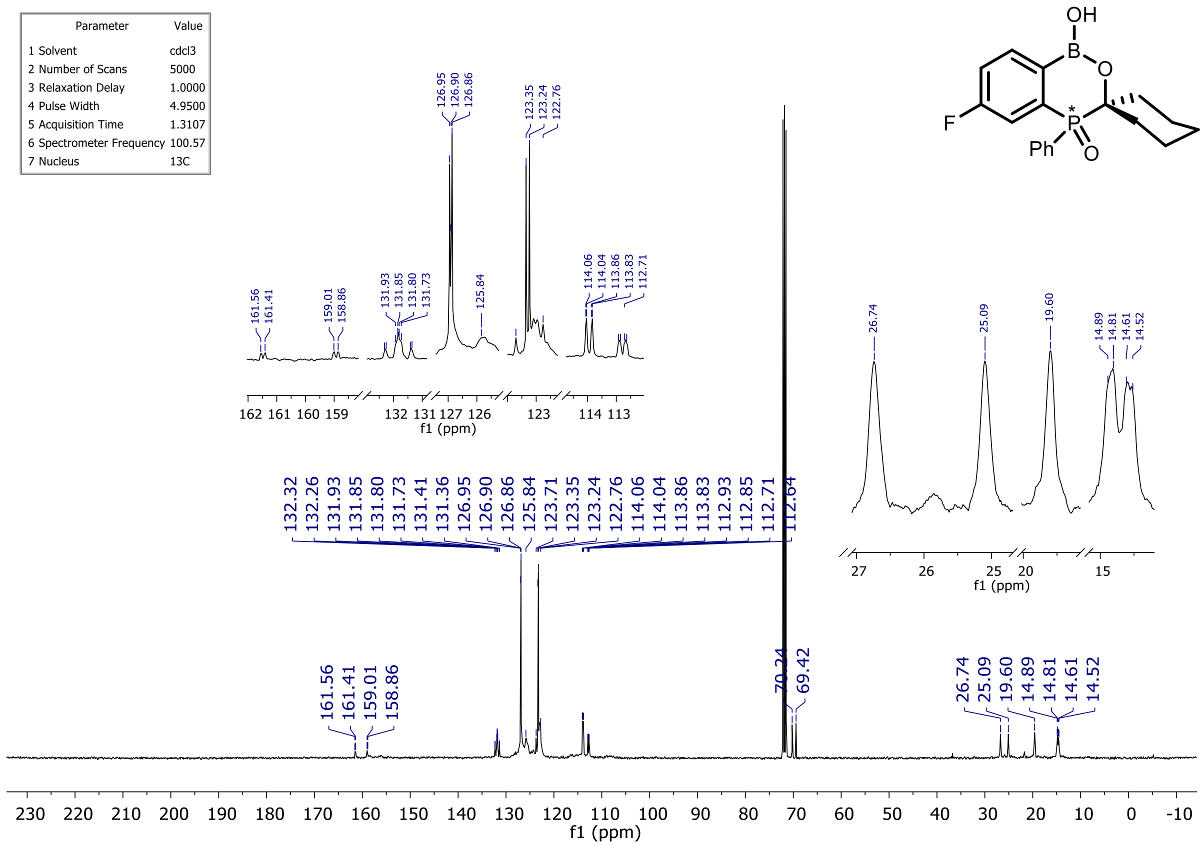

Figure S56. $^{13}C\{^{1}H\}$ NMR spectrum (101 MHz, $CDCl_3$) of **17**.

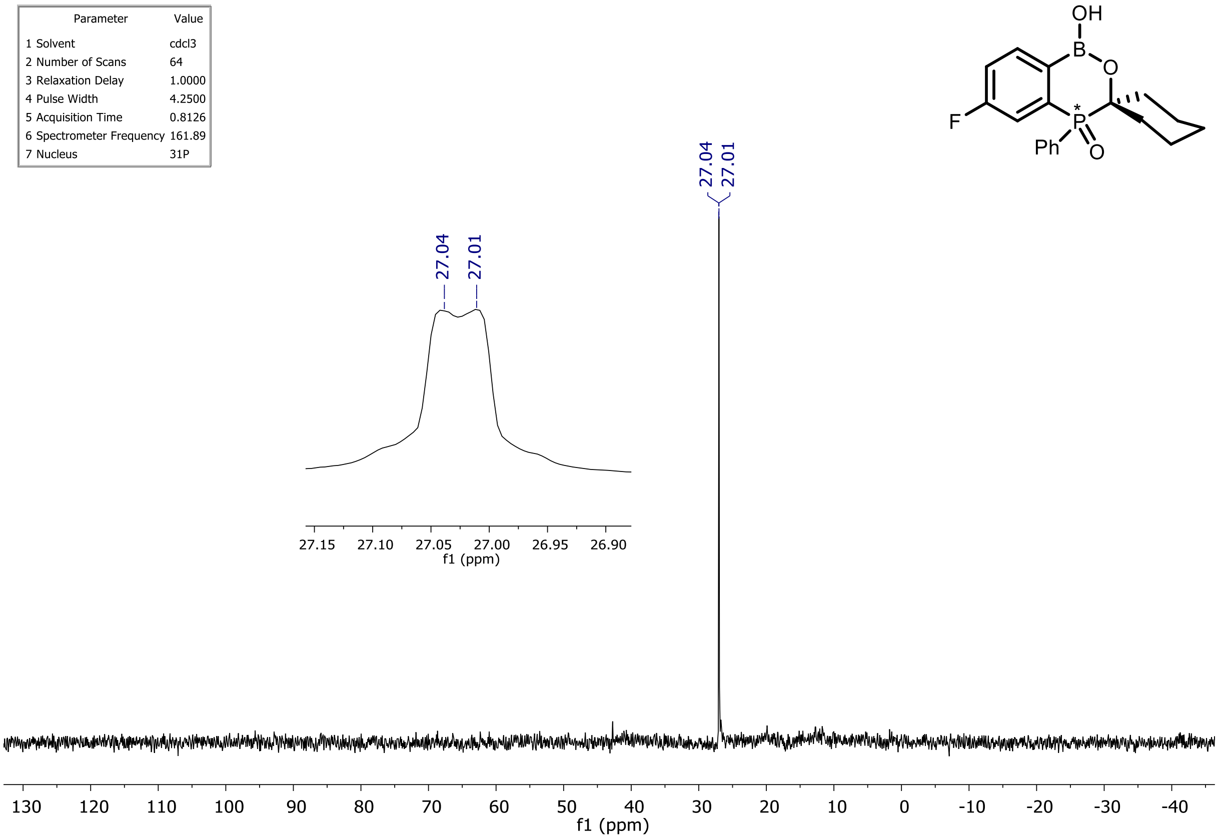

Figure S57. $^{31}P\{^{1}H\}$ NMR spectrum (162 MHz, $CDCl_3$) of **17**.

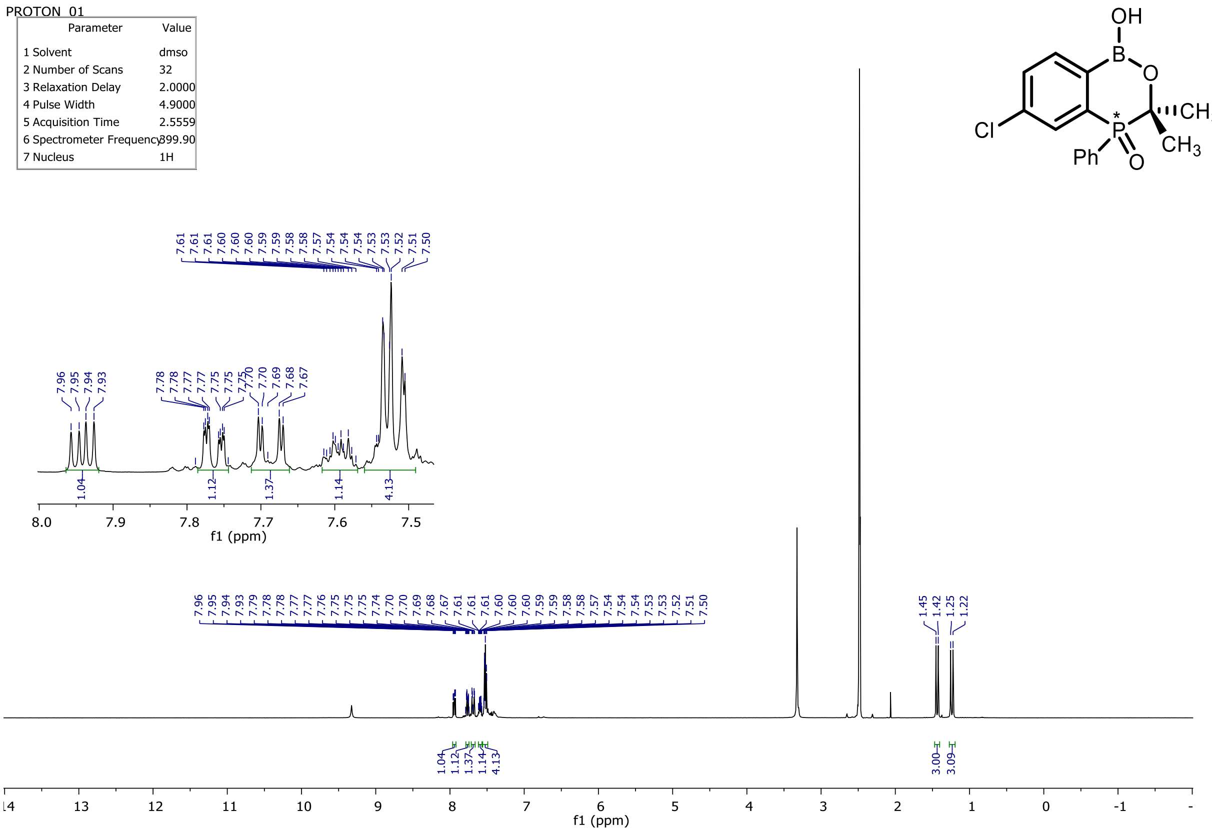

Figure S58. ^{1}H NMR spectrum (400 MHz, DMSO-d_6) of **18**.

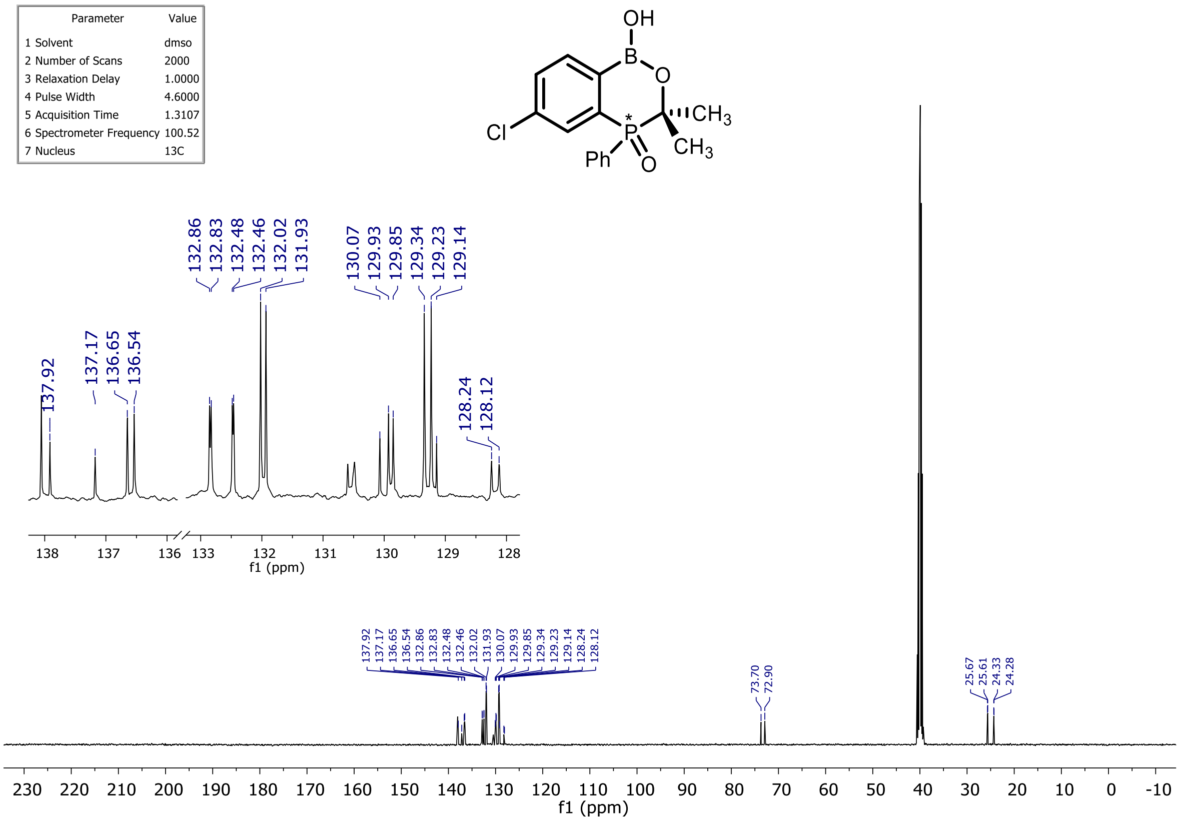

Figure S59. ^{13}C{^{1}H} NMR spectrum (101 MHz, DMSO-d_6) of **18**.

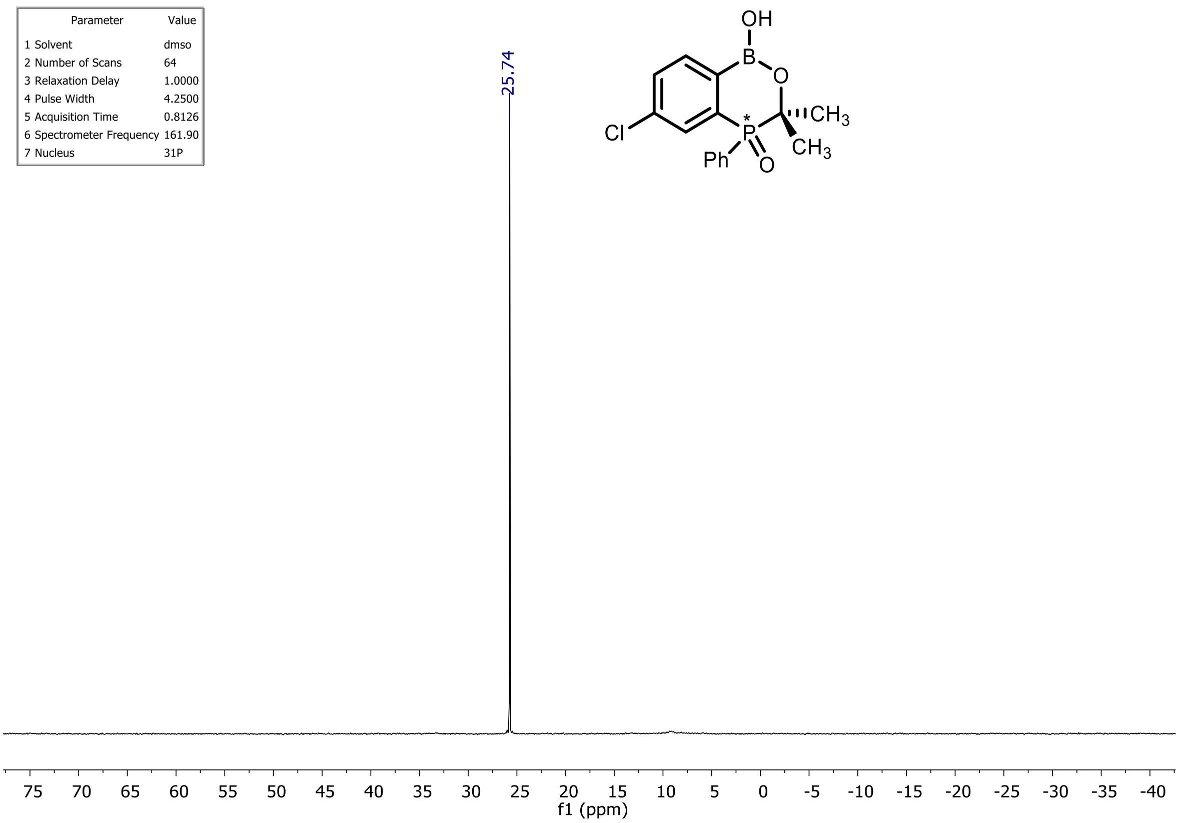

Figure S60. $^{31}P\{^1H\}$ NMR spectrum (162 MHz, DMSO-d_6) of **18**.

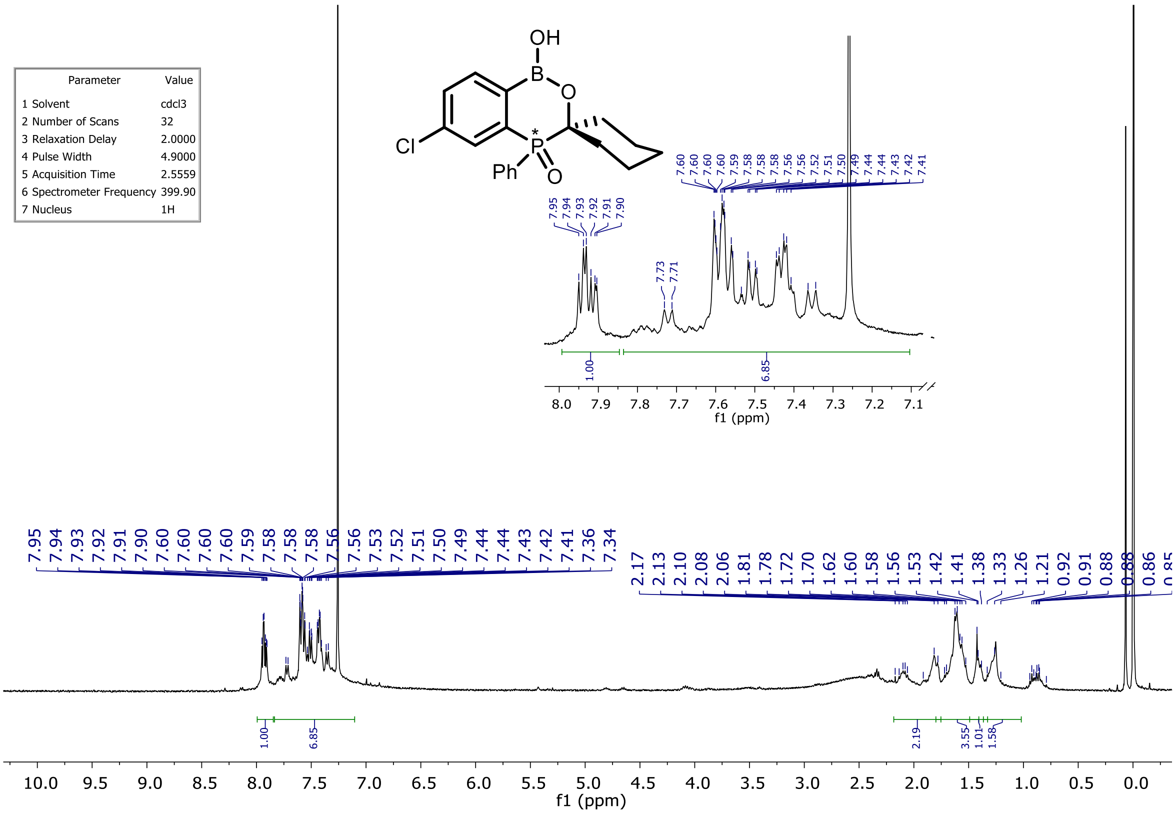

Figure S61. ^1H NMR spectrum (400 MHz, $CDCl_3$) of **19**.

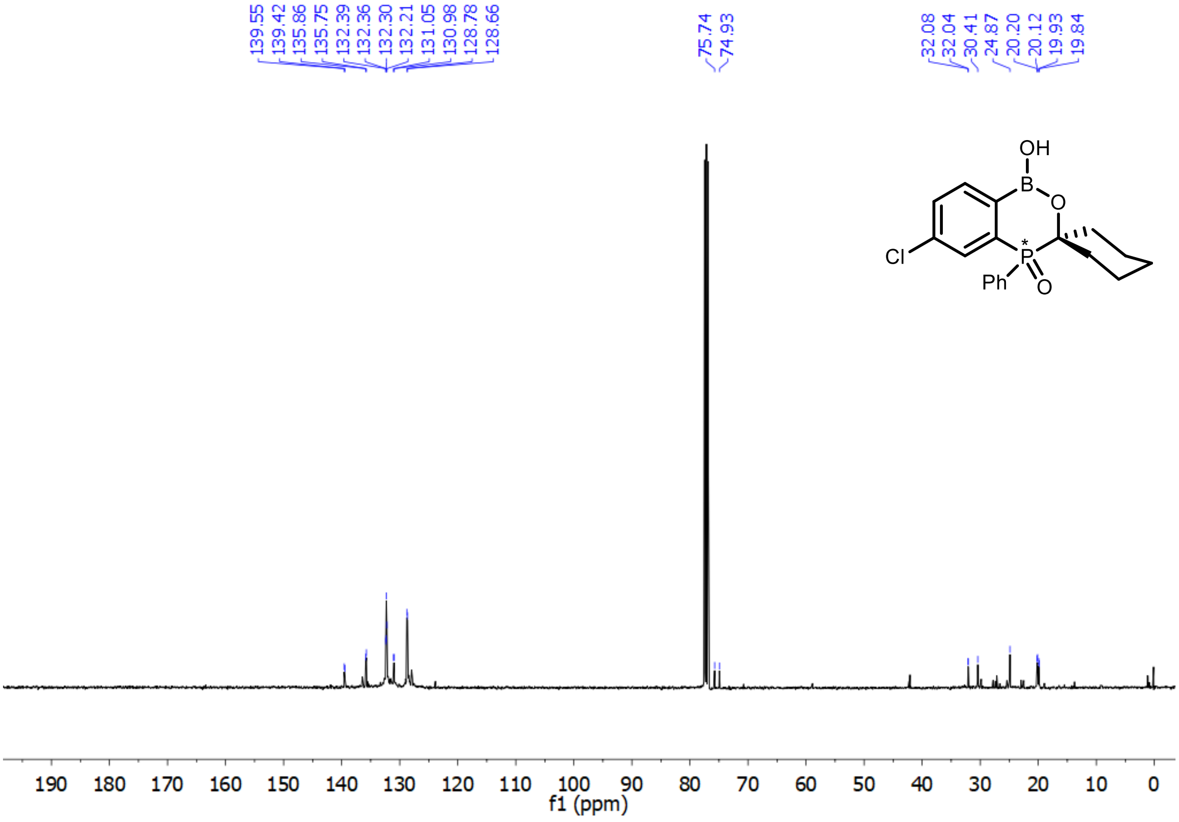

Figure S62. $^{13}C\{^{1}H\}$ NMR spectrum (101 MHz, $CDCl_3$) of **19**.

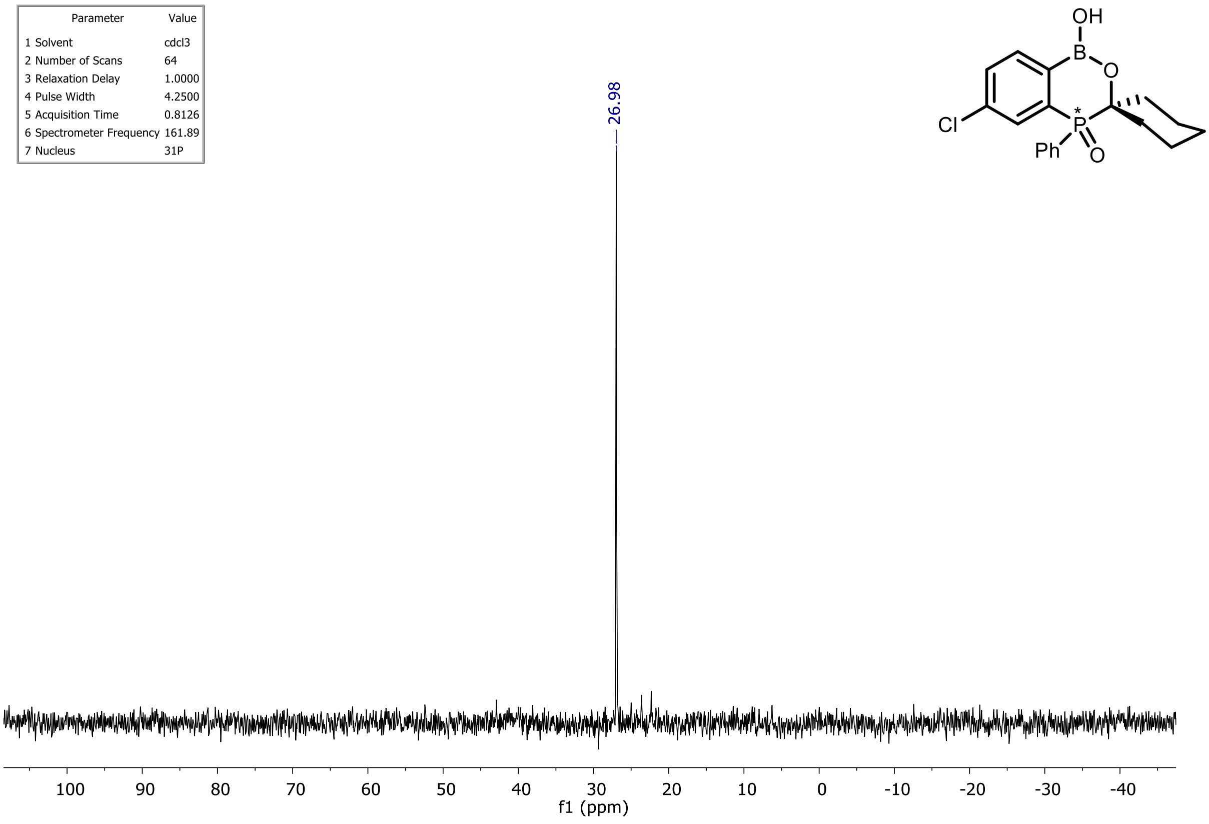

Figure S63. $^{31}P\{^{1}H\}$ NMR spectrum (162 MHz, DMSO-d_6) of **19**.

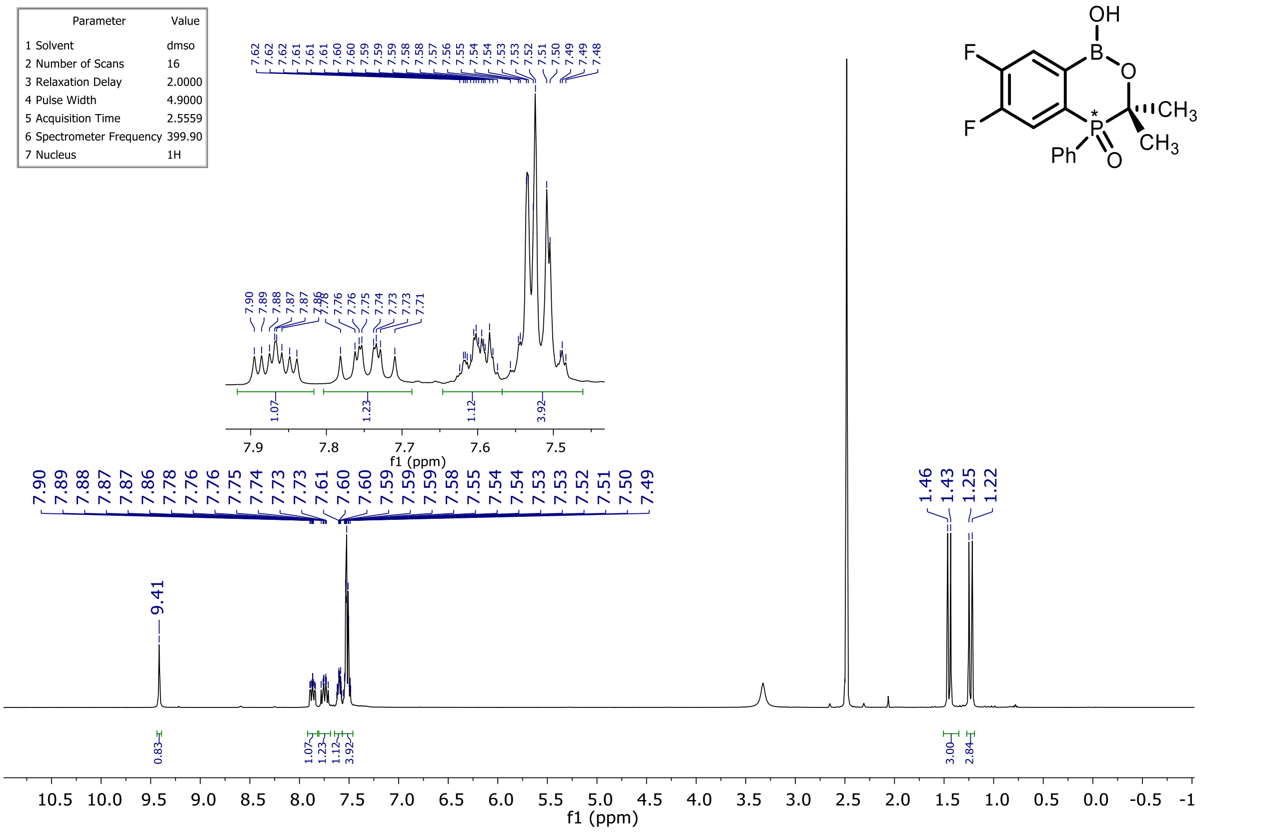

Figure S64. ^{1}H NMR spectrum (400 MHz, DMSO-d_6) of **20**.

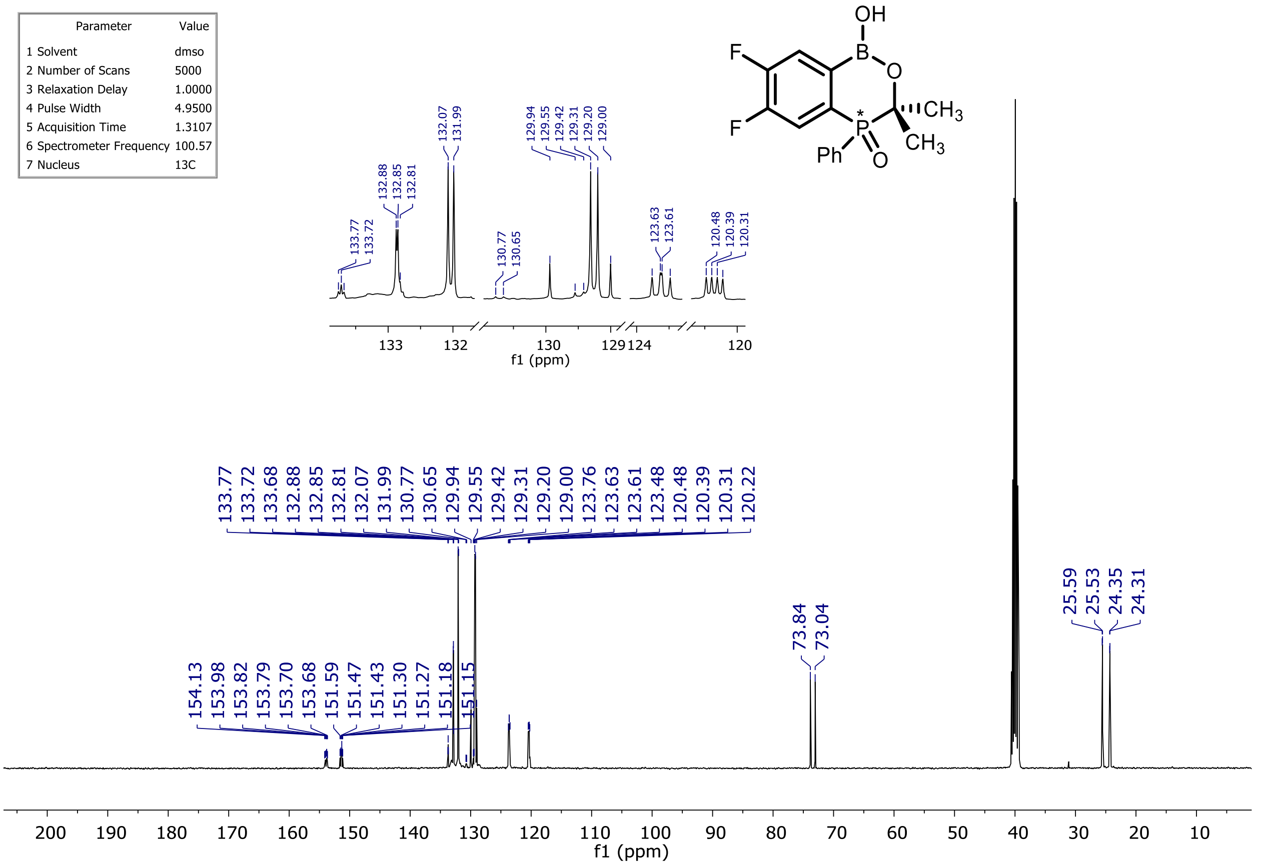

Figure S65. ^{13}C{^{1}H} NMR spectrum (101 MHz, DMSO-d_6) of **20**.

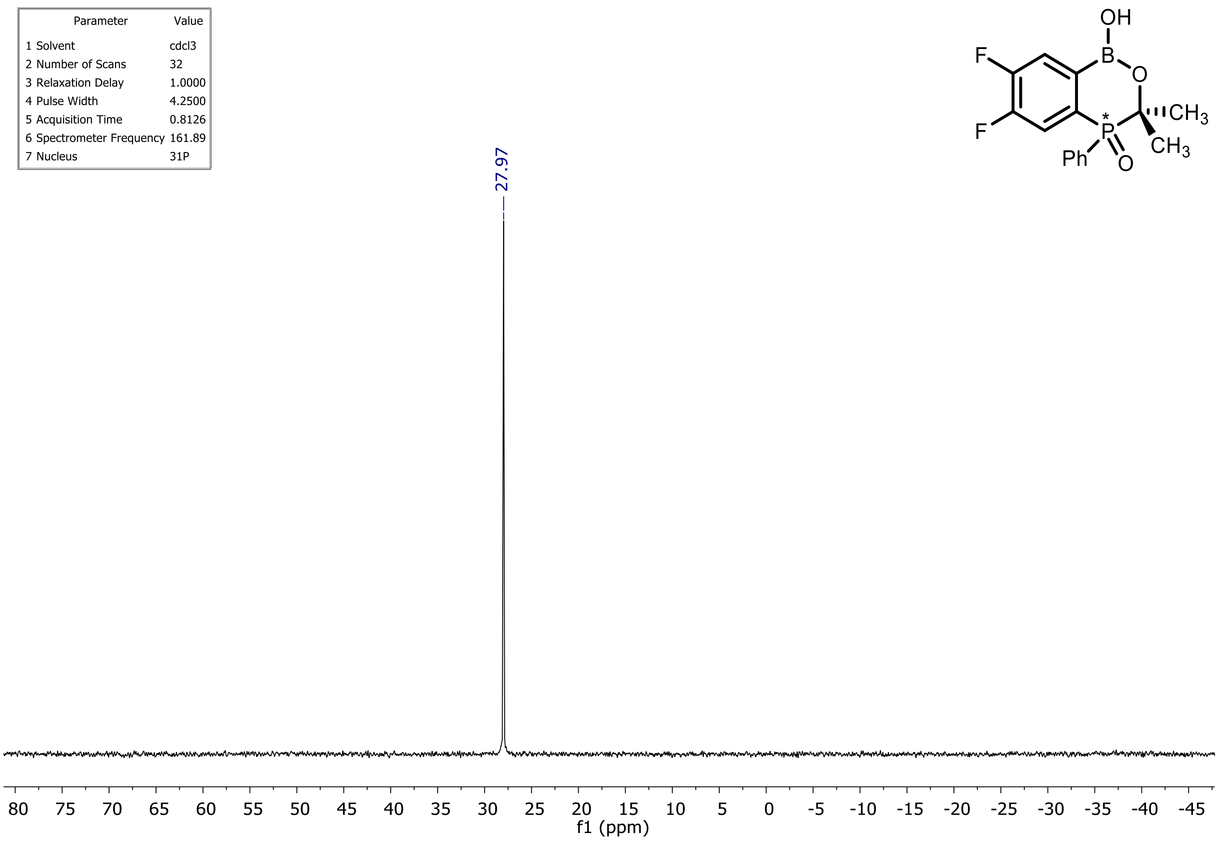

Figure S66. $^{31}P\{^{1}H\}$ NMR spectrum (162 MHz, $CDCl_3$) of **20**.

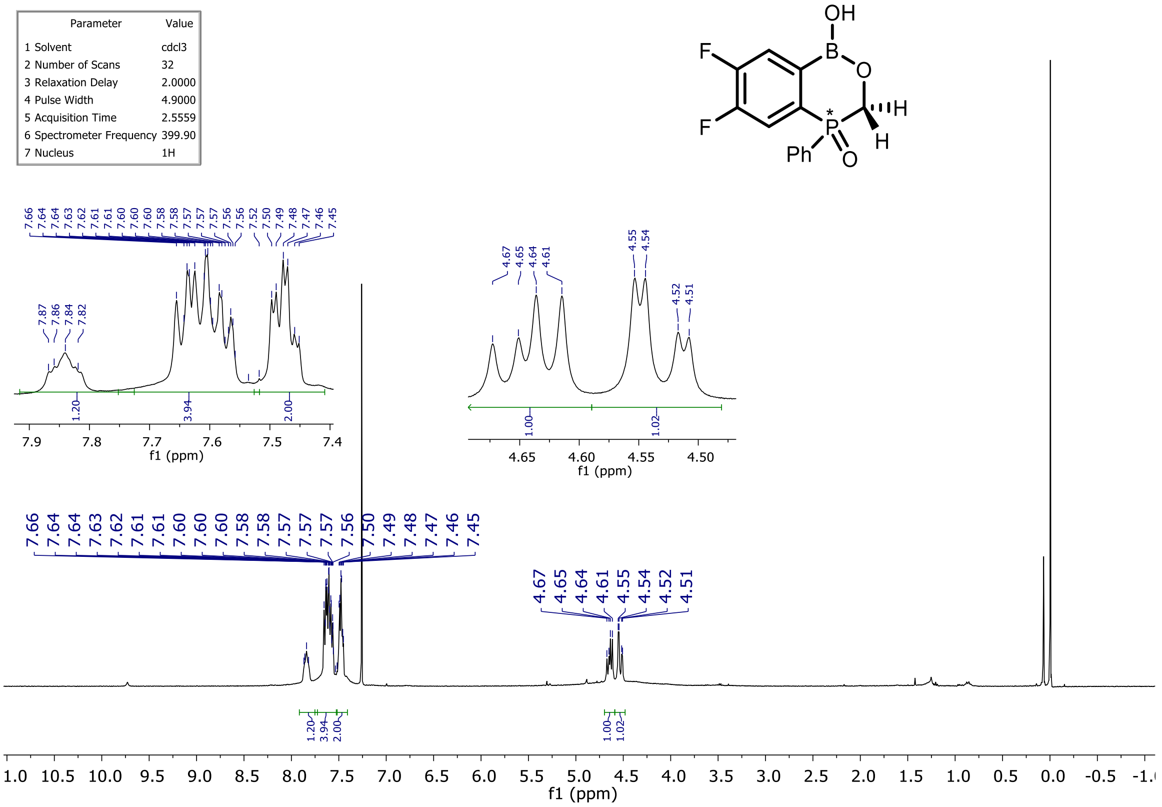

Figure S67. ^{1}H NMR spectrum (400 MHz, $CDCl_3$) of **21**.

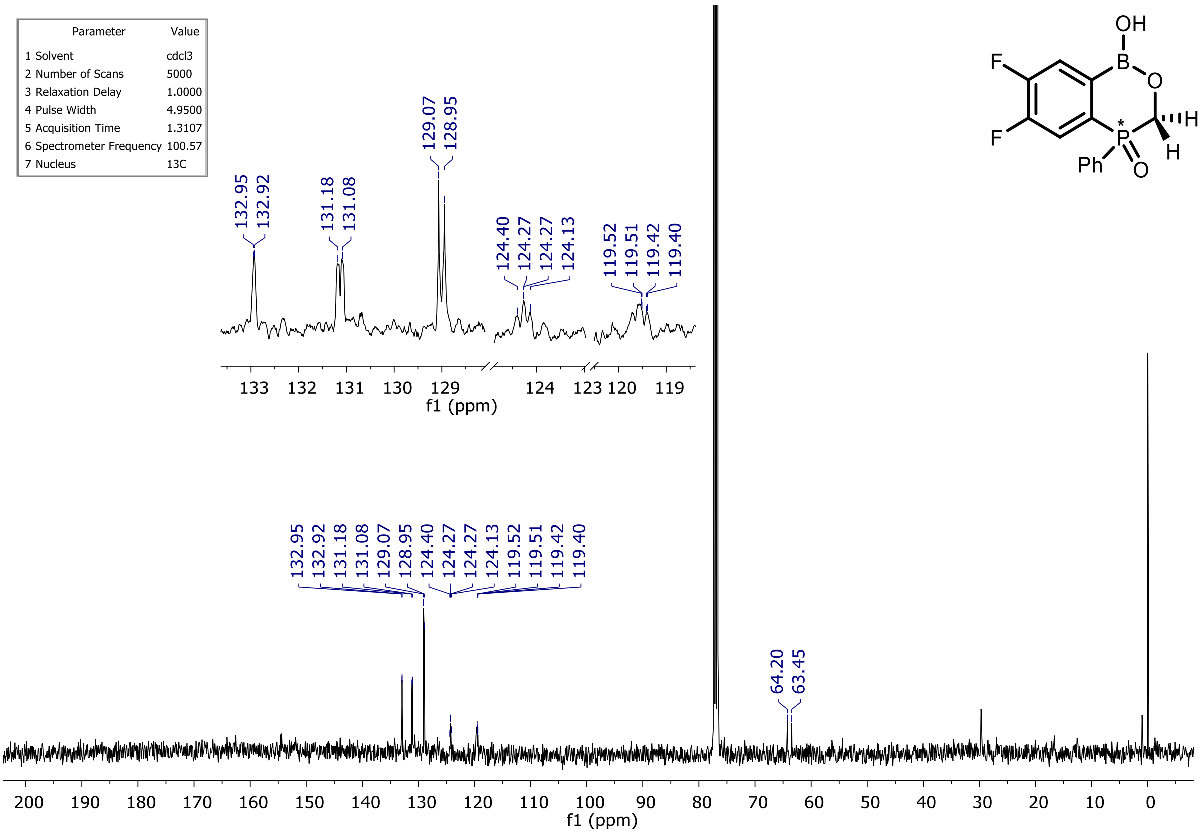

Figure S68. $^{13}C\{^{1}H\}$ NMR spectrum (101 MHz, $CDCl_3$) of **21**. Signals of fluorine- and phosphorus-bound carbon atoms are not observed due to low intensity.

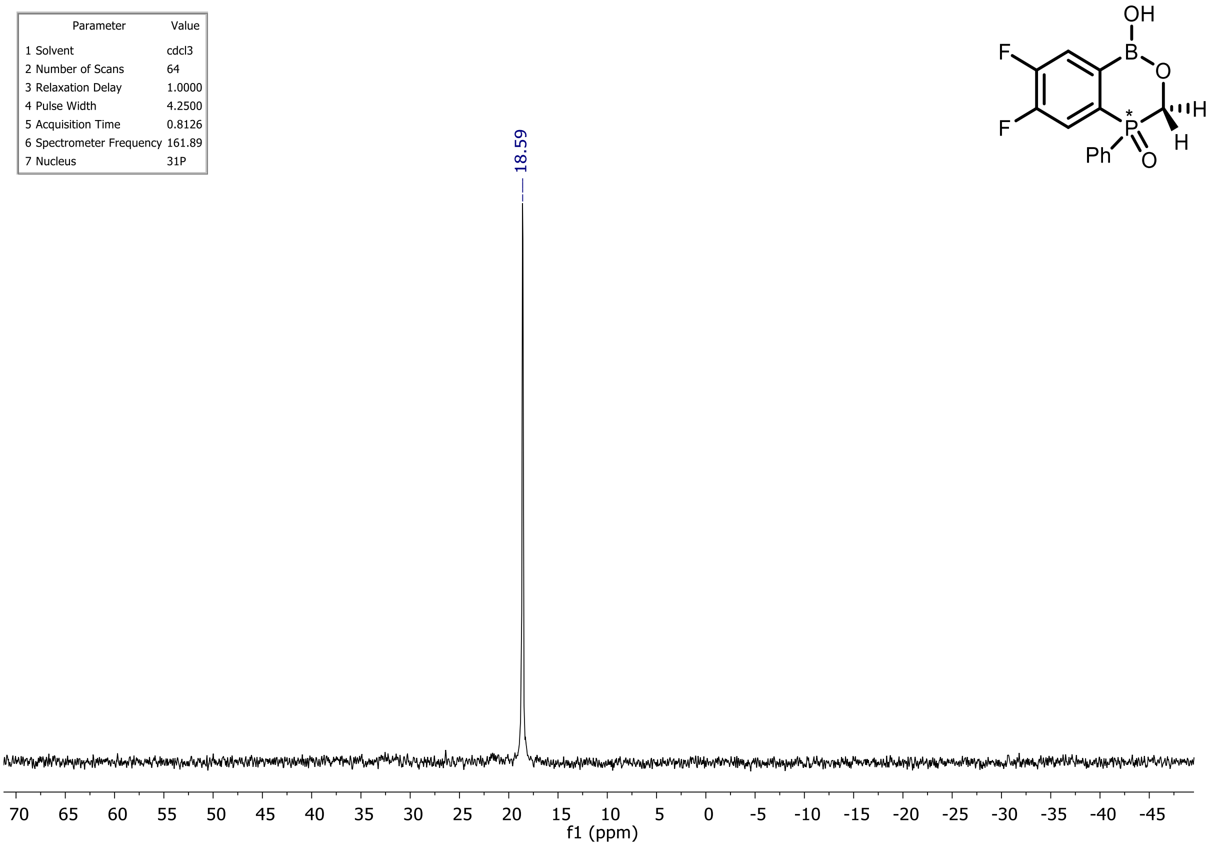

Figure S69. $^{31}P\{^{1}H\}$ NMR spectrum (162 MHz, $CDCl_3$) of **21**.

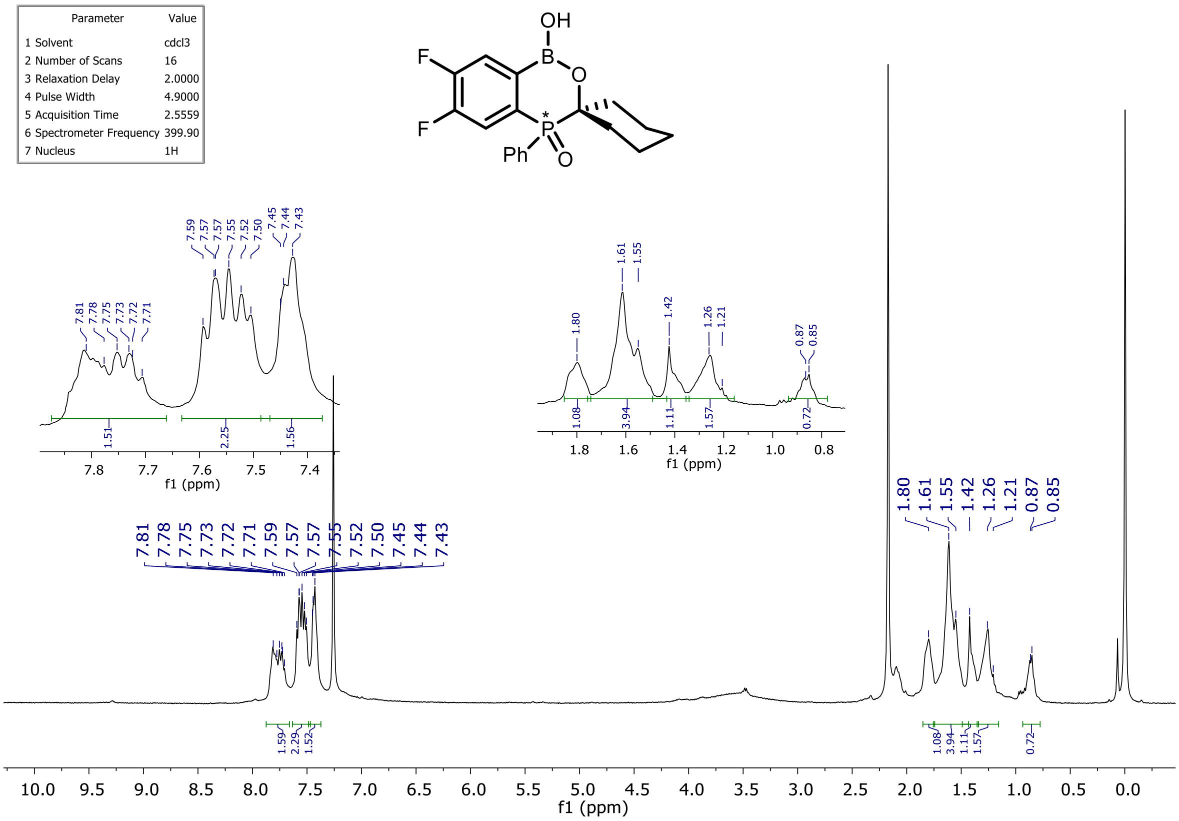

Figure S70. ^{1}H NMR spectrum (400 MHz, $CDCl_3$) of **22**.

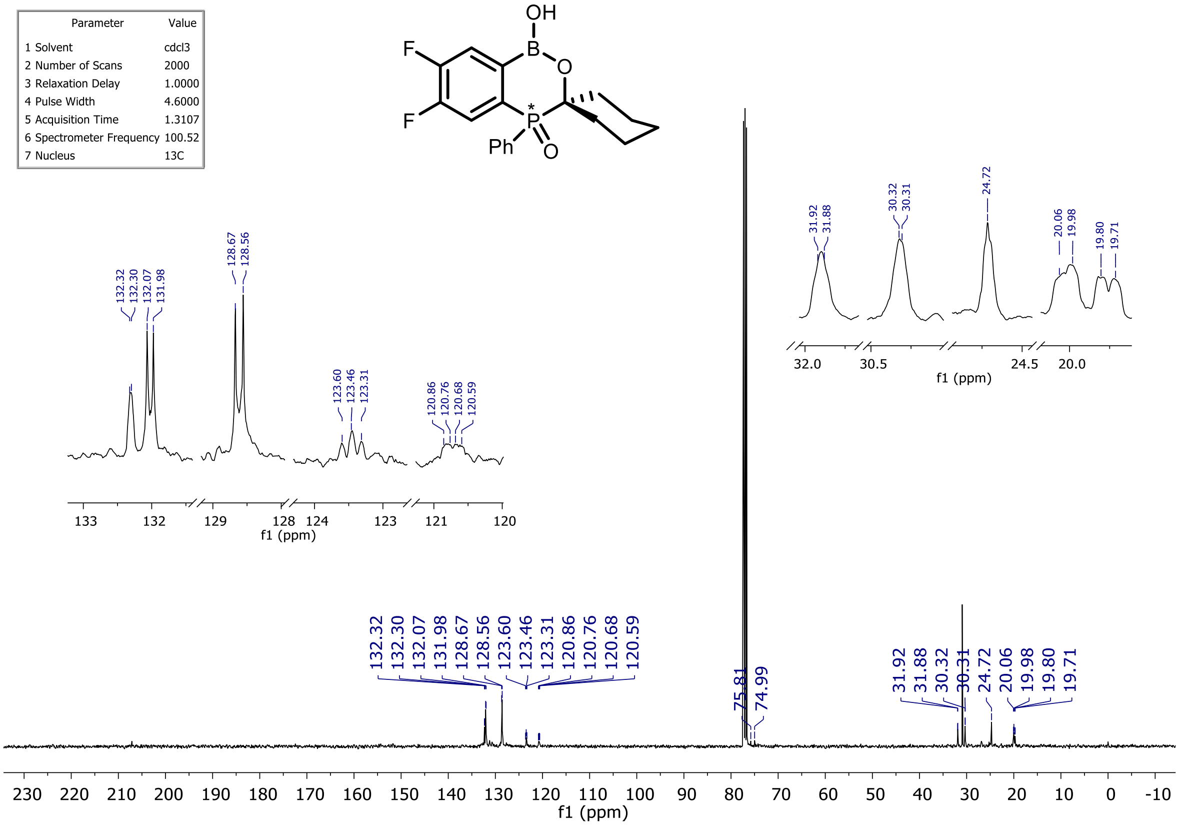

Figure S71. $^{13}C\{^{1}H\}$ NMR spectrum (101 MHz, $CDCl_3$) of **22**. Signals of aromatic C(F) and C(P) atoms are not observed due to low intensity.

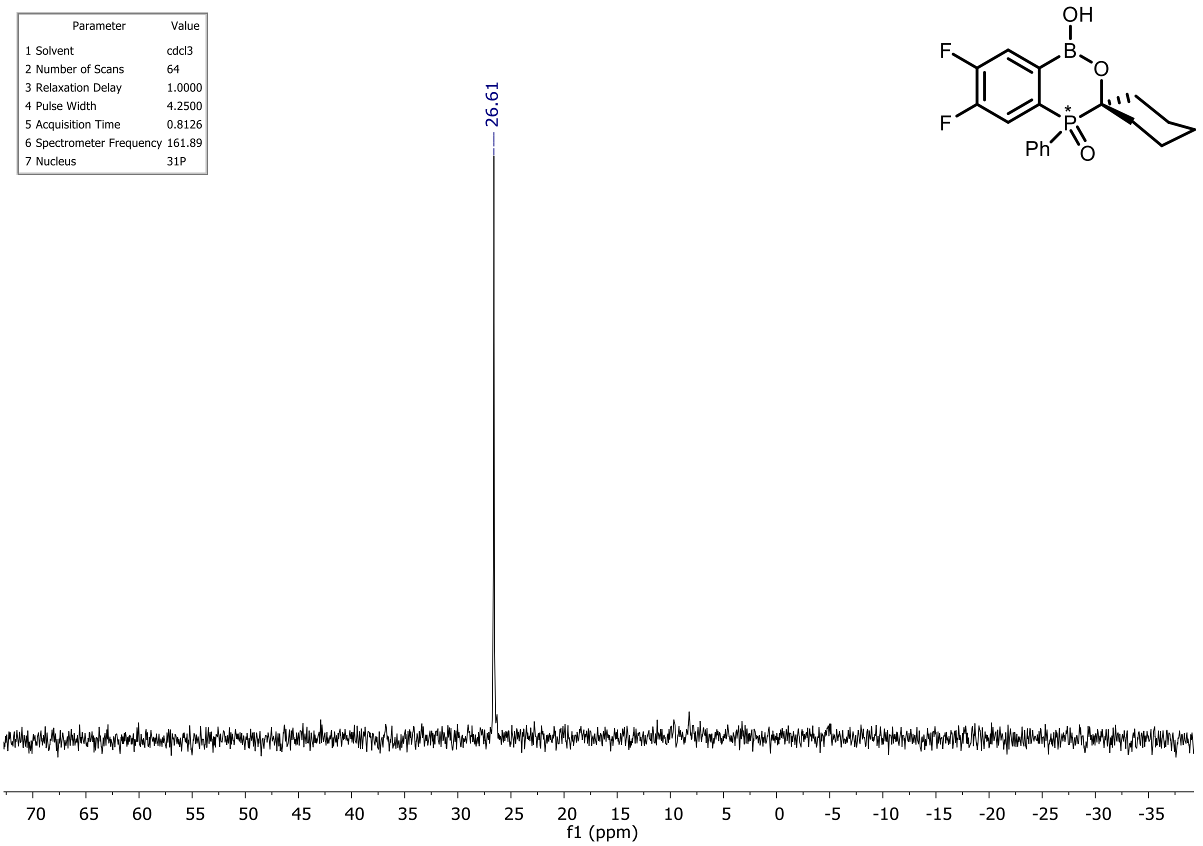

Figure S72. $^{31}P\{^{1}H\}$ NMR spectrum (162 MHz, $CDCl_3$) of **23**.

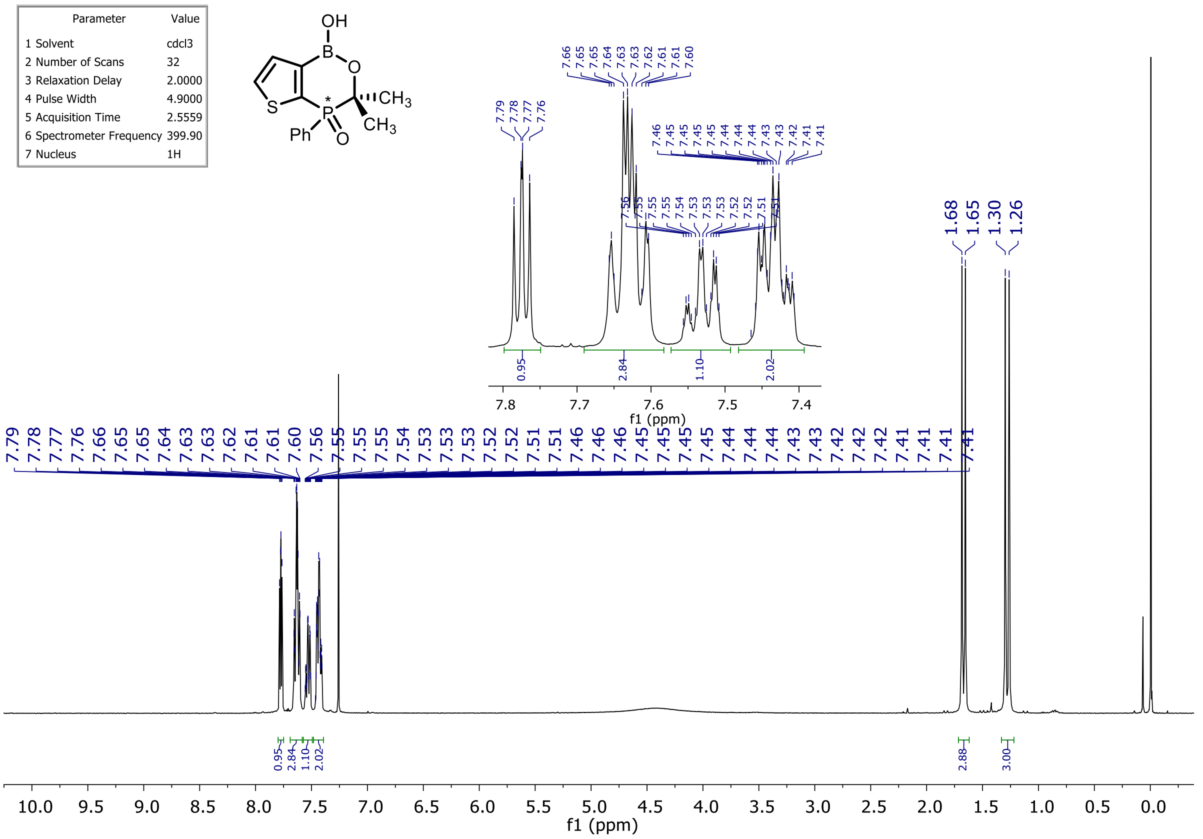

Figure S73. ^{1}H NMR spectrum (400 MHz, $CDCl_3$) of **23**.

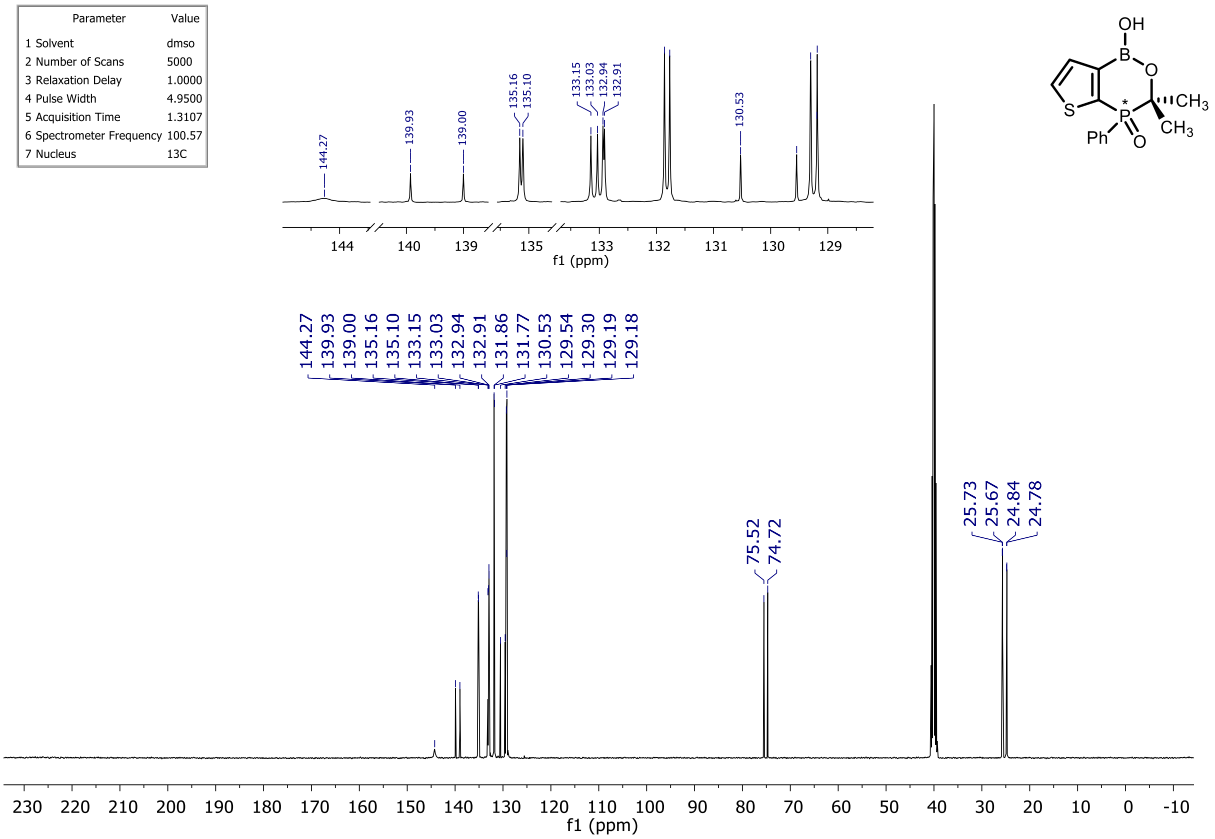

Figure S74. $^{13}C\{^{1}H\}$ NMR spectrum (101 MHz, DMSO-d_6) of **23**.

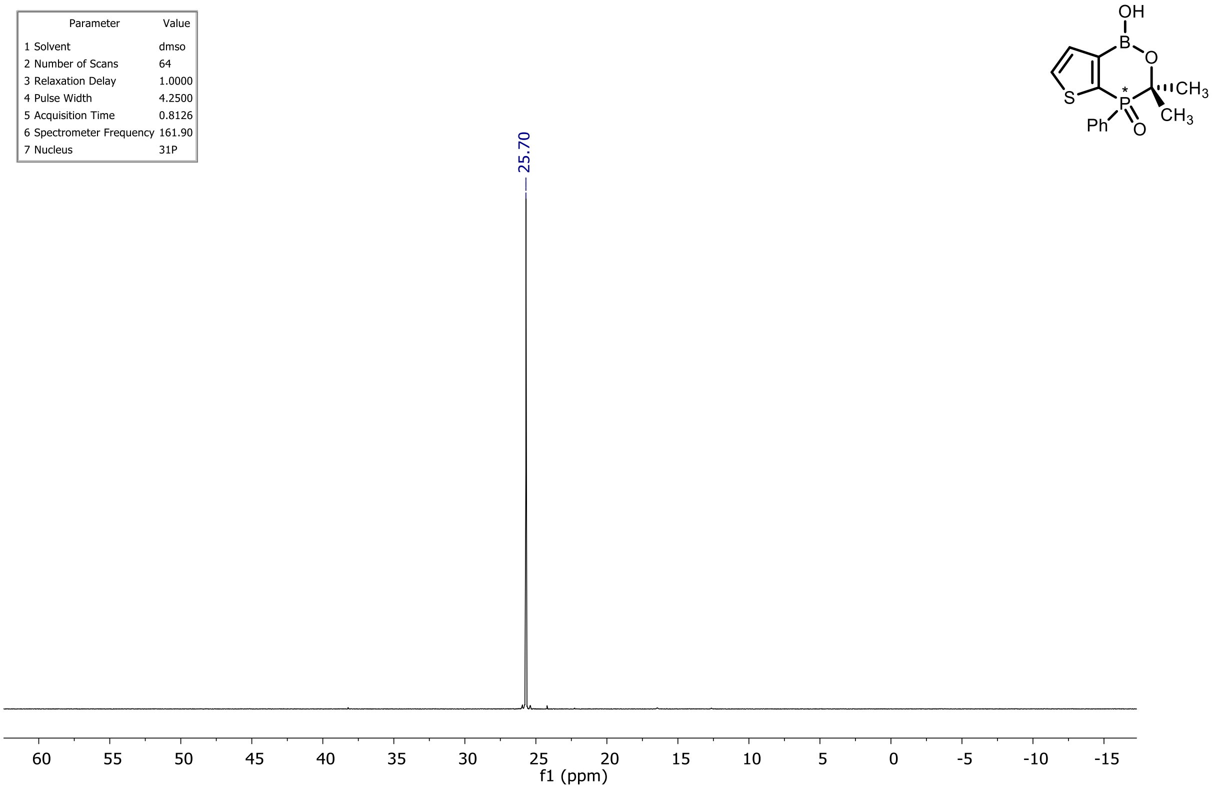

Figure S75. $^{31}P\{^{1}H\}$ NMR spectrum (162 MHz, DMSO-d_6) of **23**.

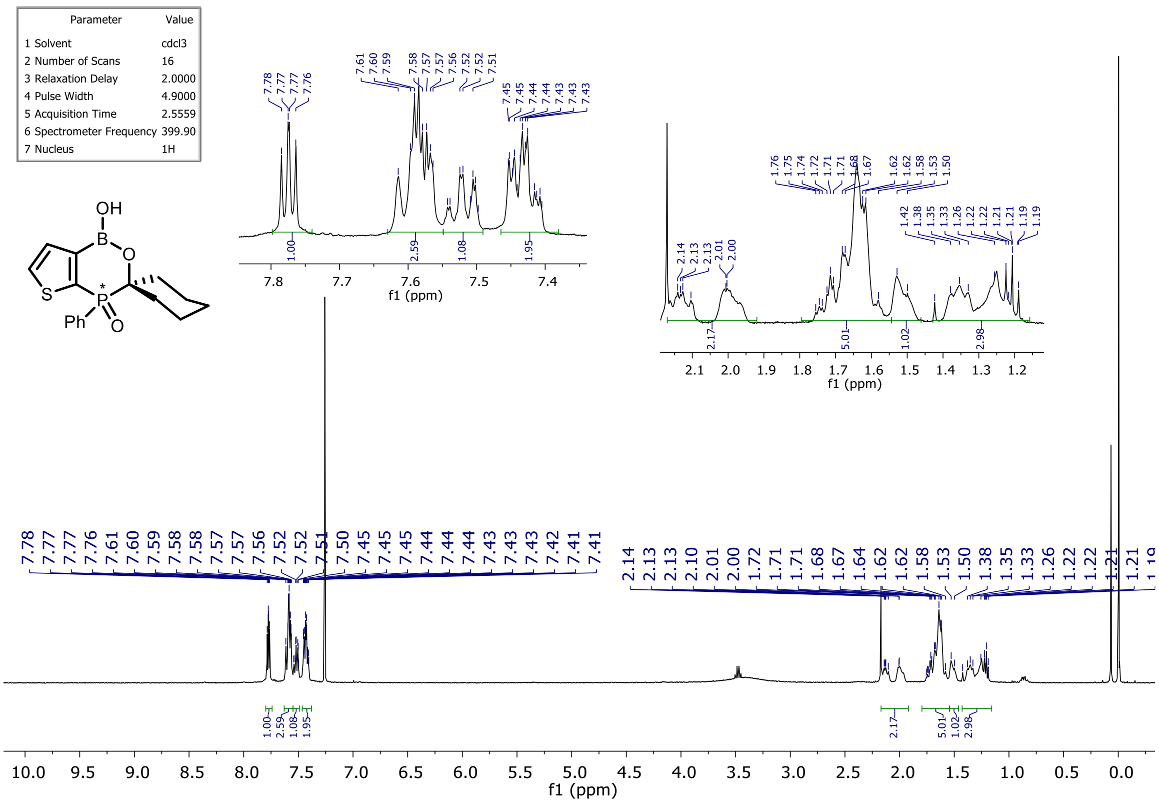

Figure S76. ^{1}H NMR spectrum (400 MHz, $CDCl_3$) of **24**.

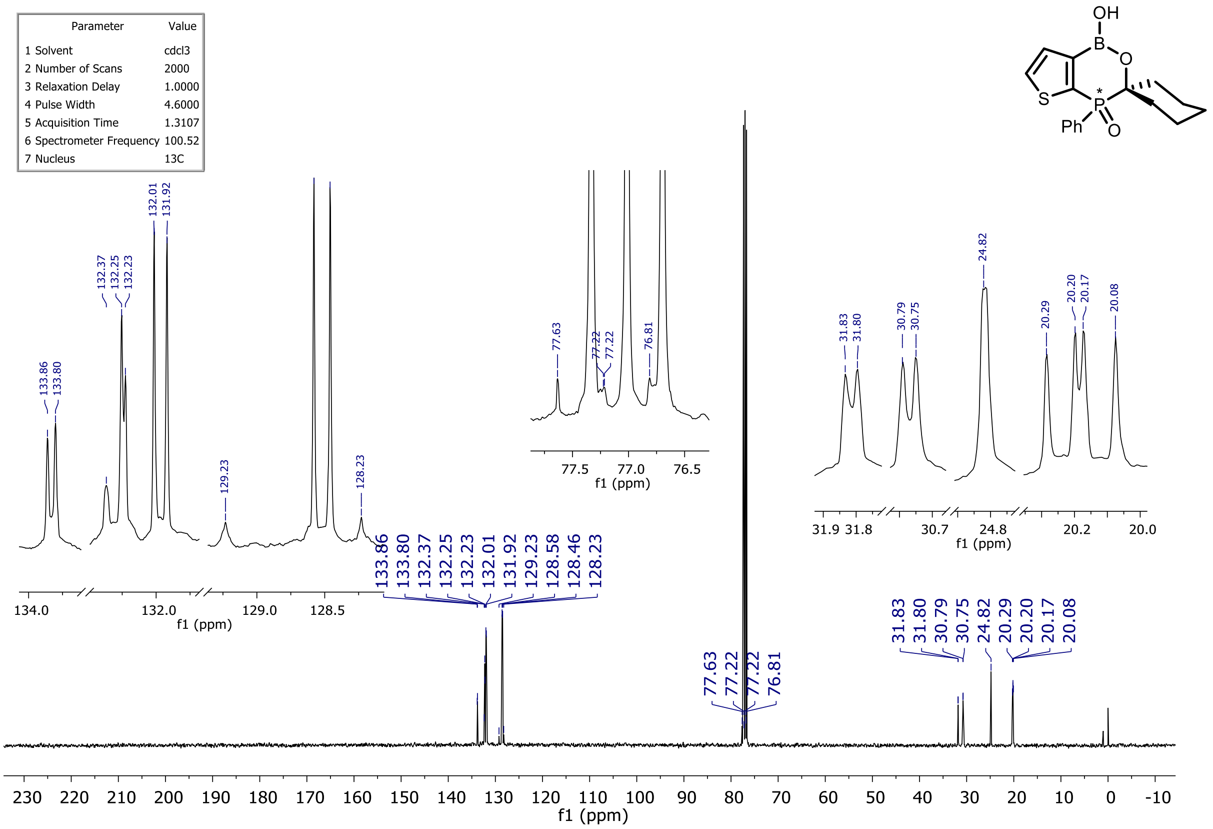

Figure S77. $^{13}C\{^{1}H\}$ NMR spectrum (101 MHz, $CDCl_3$) of **24**.

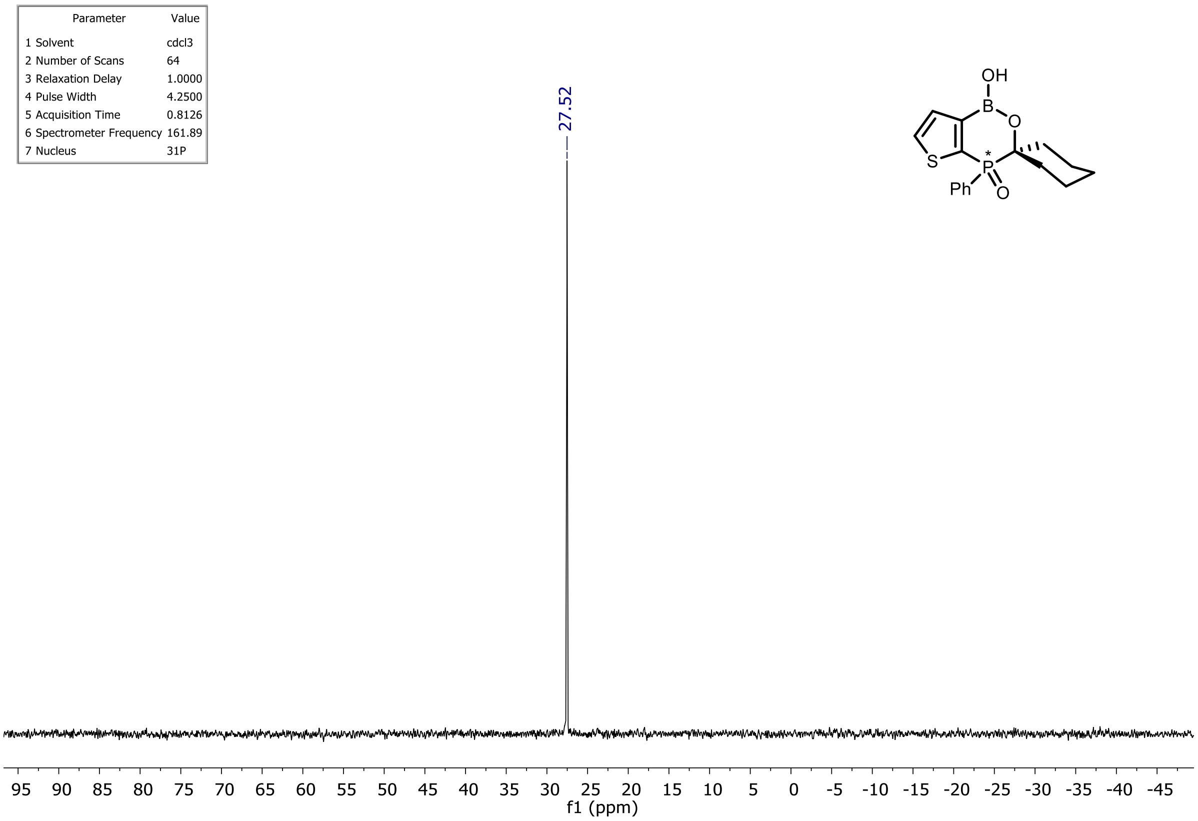

Figure S78. $^{31}P\{^{1}H\}$ NMR spectrum (162 MHz, DMSO-d_6) of **24**.

Mass spectra

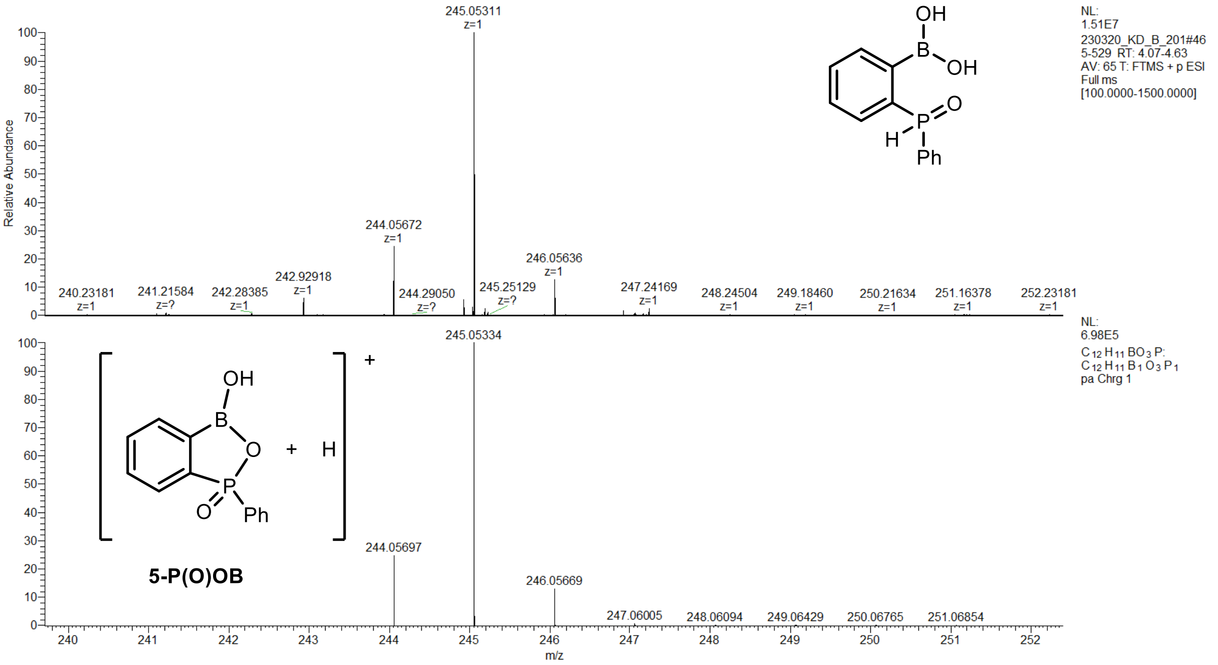

Figure S79. HRMS spectrum (ESI, positive ion mode) of **5**. The basic formula $C_{12}H_{13}BO_3P^+$ $[M+H]^+$ was not found. The calculated spectrum of the formula $C_{12}H_{11}BO_3P^+$ $[M–H_2+H]^+$ is given in the bottom.

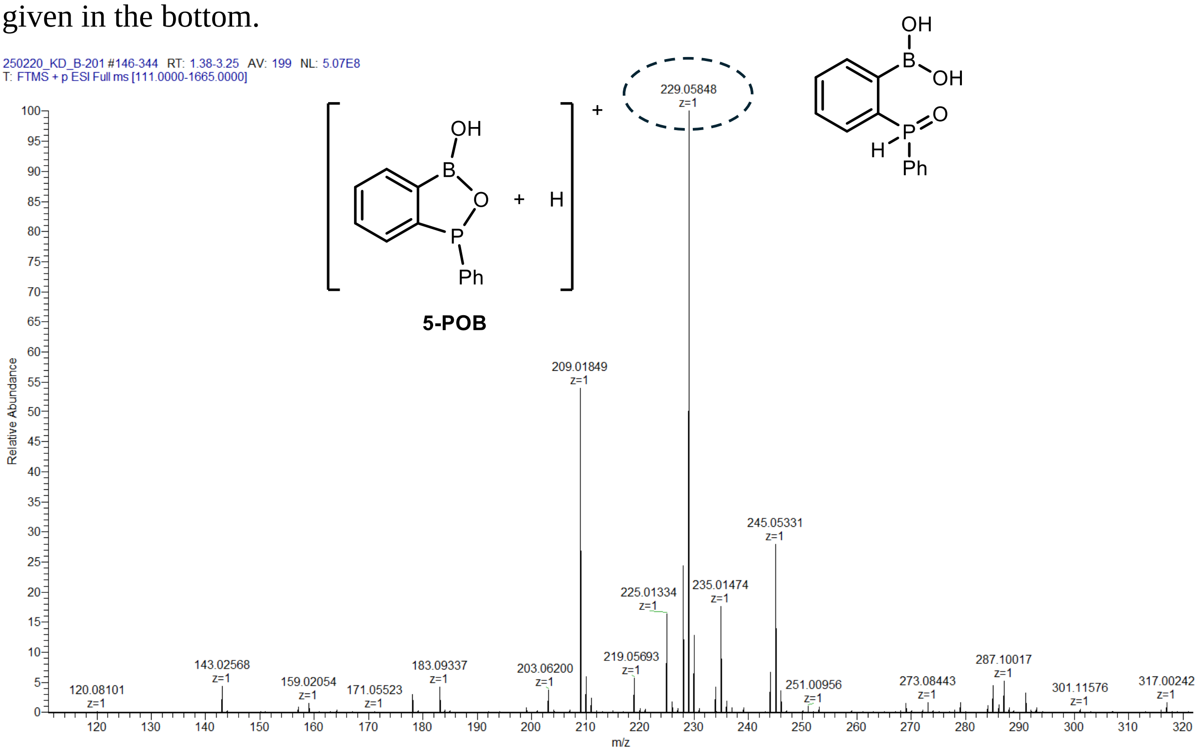

Figure S80. HRMS spectrum (ESI, positive ion mode) of **5.** The base peak attributable to the protonated **5-POB** species is marked.

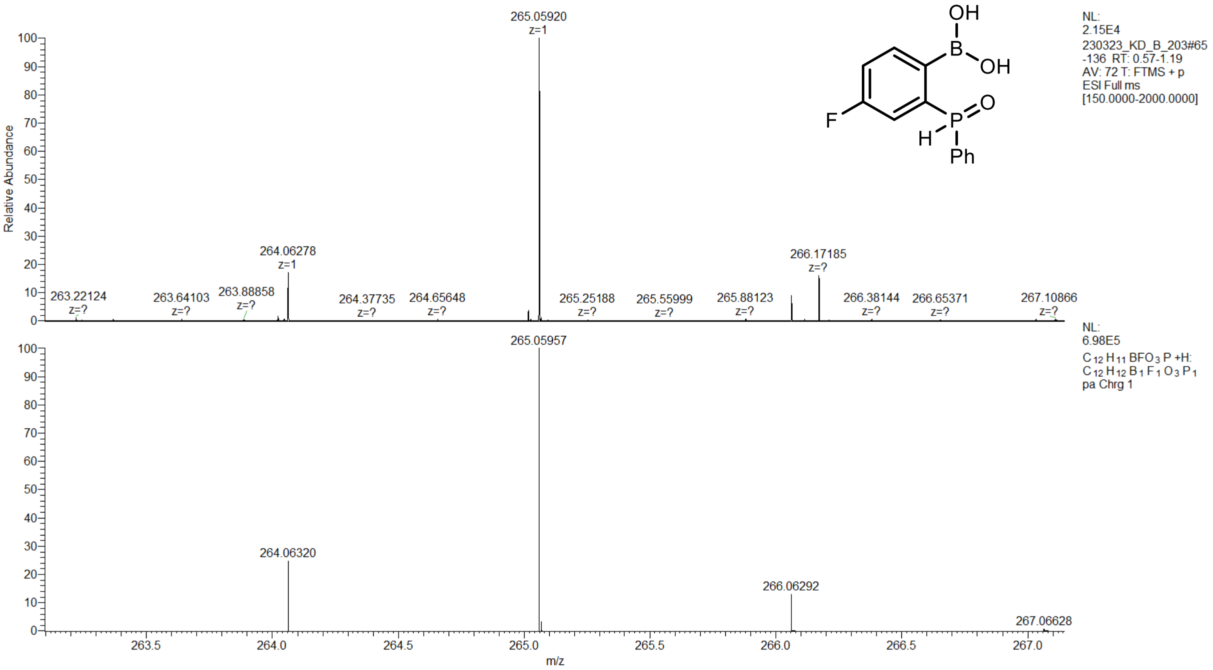

Figure S81. HRMS spectrum (ESI, positive ion mode) of **6**. The calculated spectrum of the formula $C_{12}H_{12}BFO_3P^+$ $[M+H]^+$ is given in the bottom.

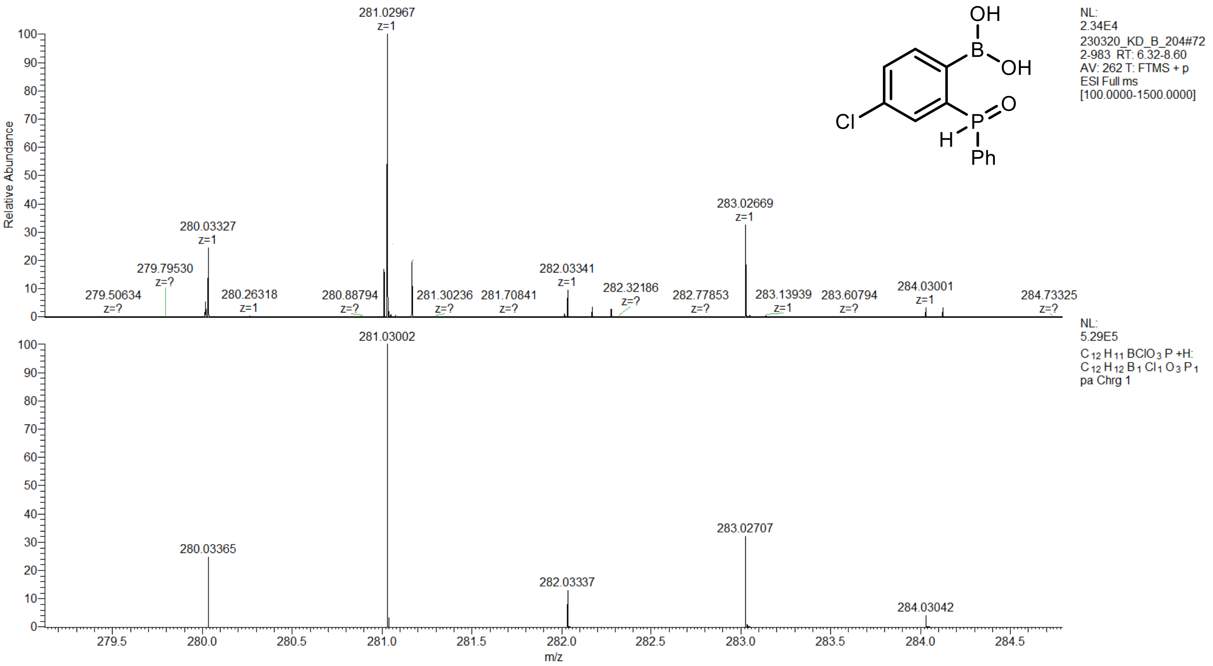

Figure S82. HRMS spectrum (ESI, positive ion mode) of **7**. The calculated spectrum of the formula $C_{12}H_{12}BClO_3P^+$ $[M+H]^+$ is given in the bottom.

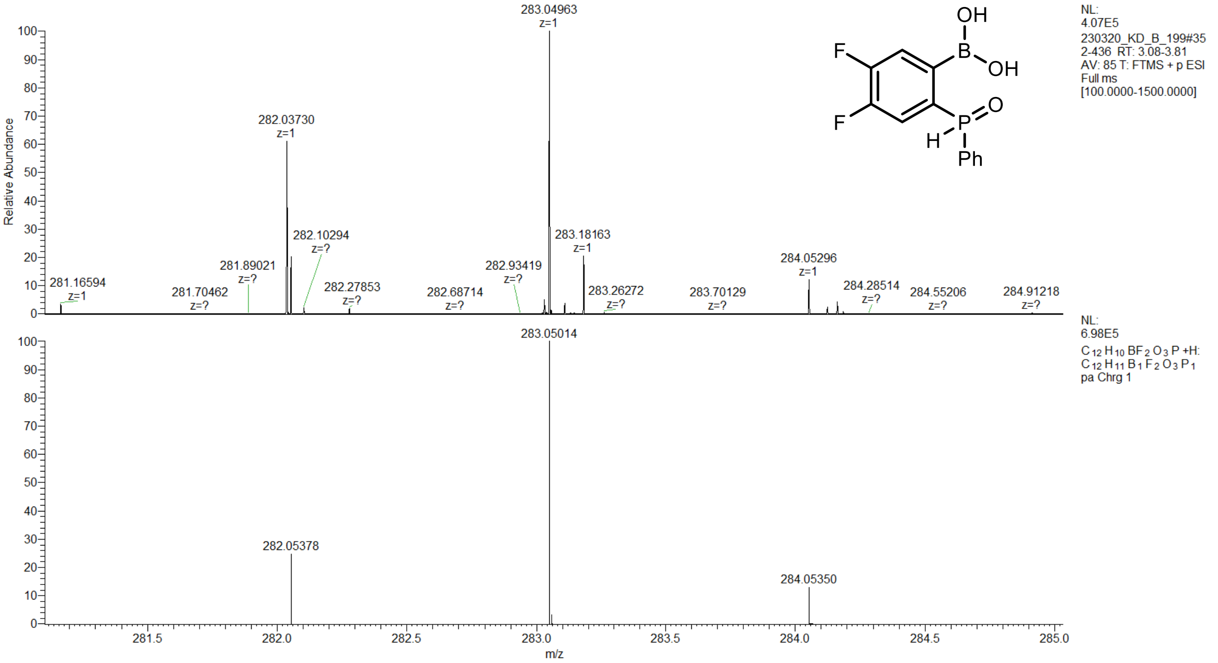

Figure S83. HRMS spectrum (ESI, positive ion mode) of **8**. The calculated spectrum of the formula $C_{12}H_{11}BF_2O_3P^+$ $[M+H]^+$ is given in the bottom.

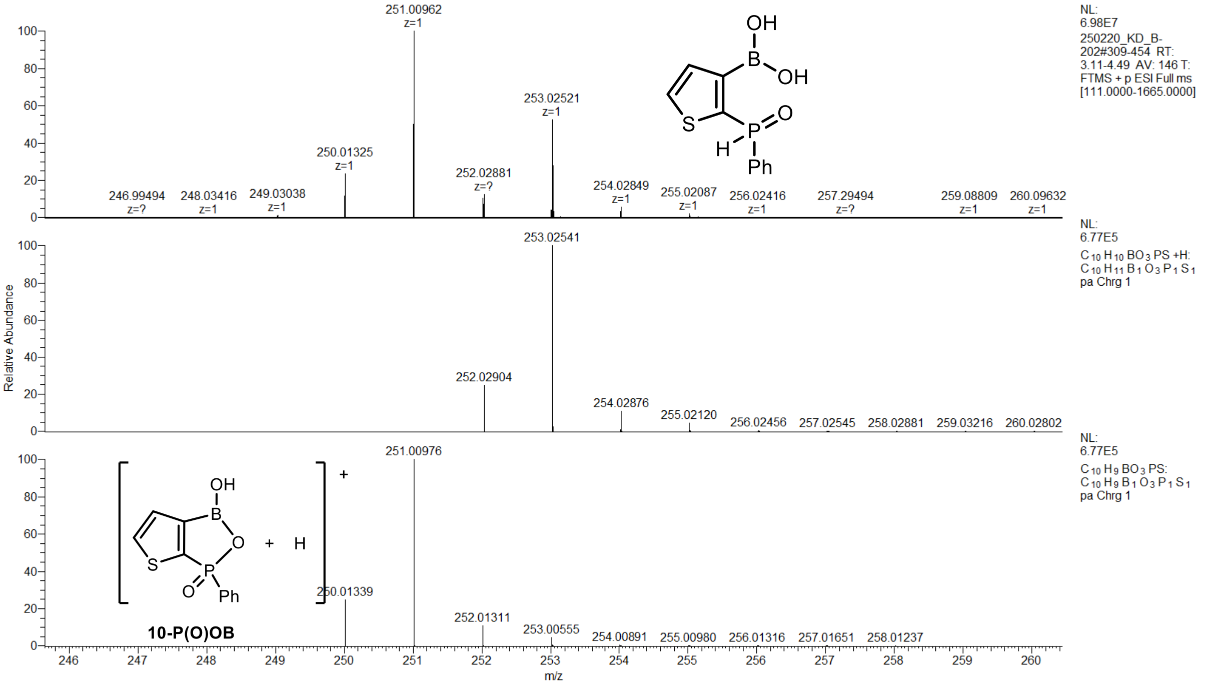

Figure S84. HRMS spectrum (ESI, positive ion mode) of **10**. The calculated spectra of the formulae $C_{10}H_{11}BO_3PS^+$ $[M+H]^+$ and $C_{10}H_9BO_3PS^+$ $[M–H_2+H]^+$ are given in the middle and the bottom, respectively.

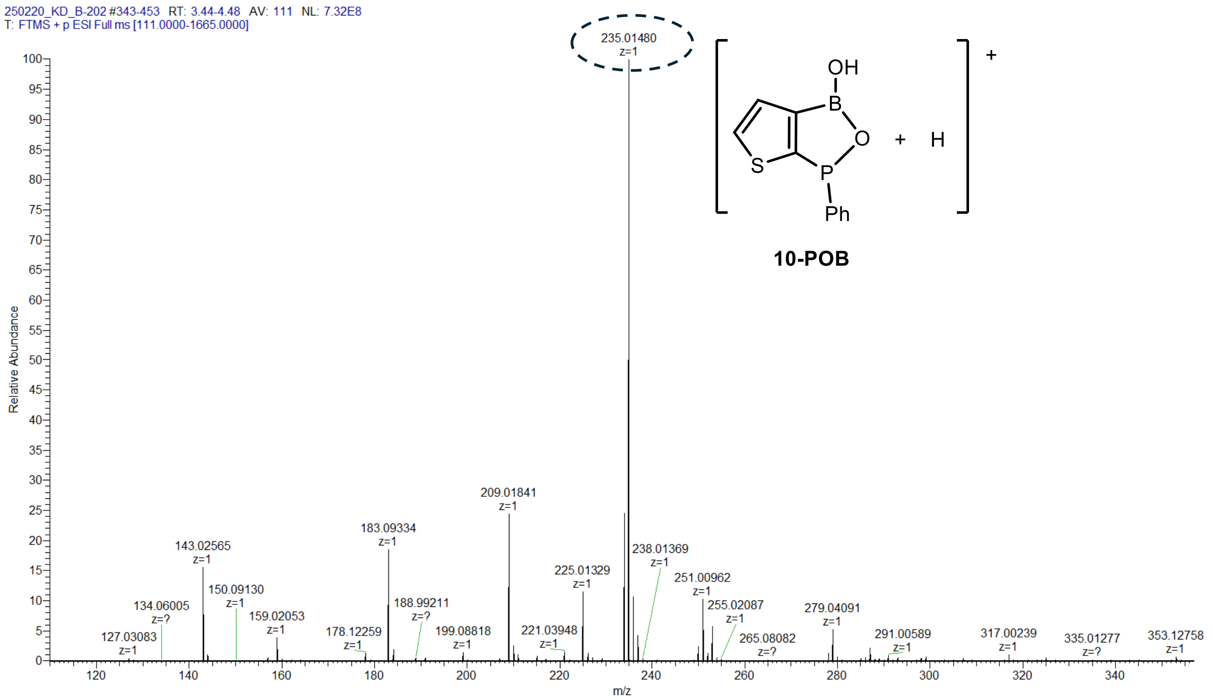

Figure S85. HRMS spectrum (ESI, positive ion mode) of **10**. The base peak can be assigned to the species **10-POB**.

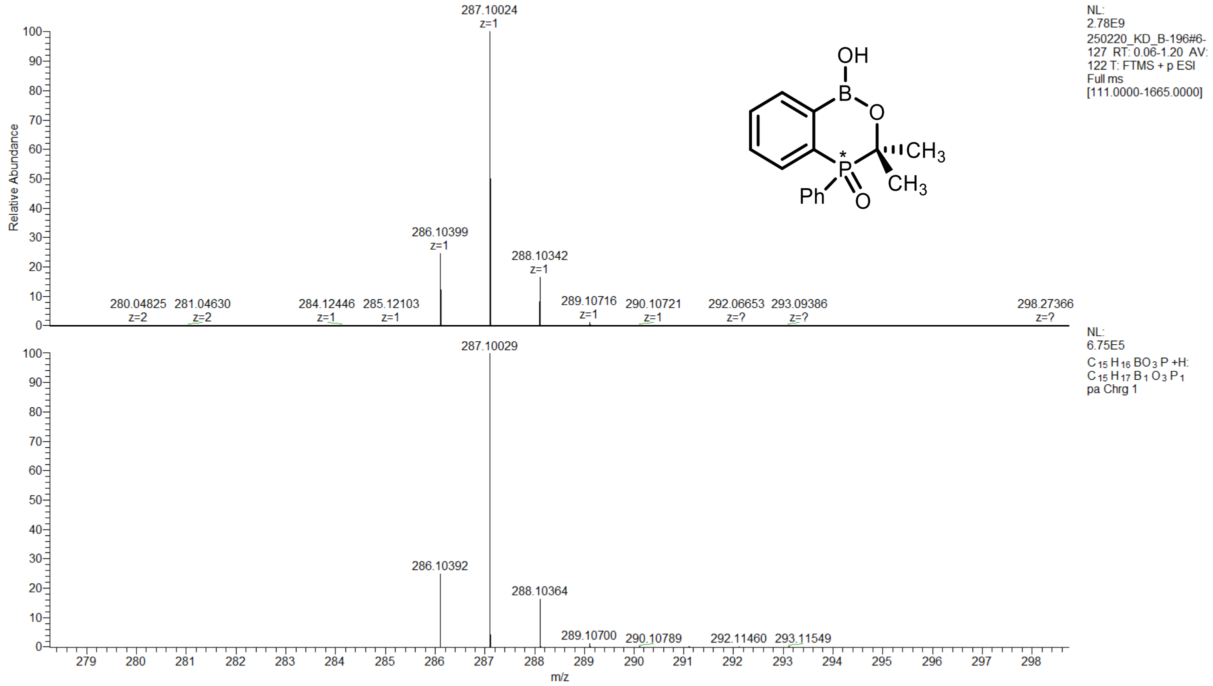

Figure S86. HRMS spectrum (ESI, positive ion mode) of **11**. The calculated spectrum of the formula $C_{15}H_{17}BO_3P^+$ $[M+H]^+$ is given in the bottom.

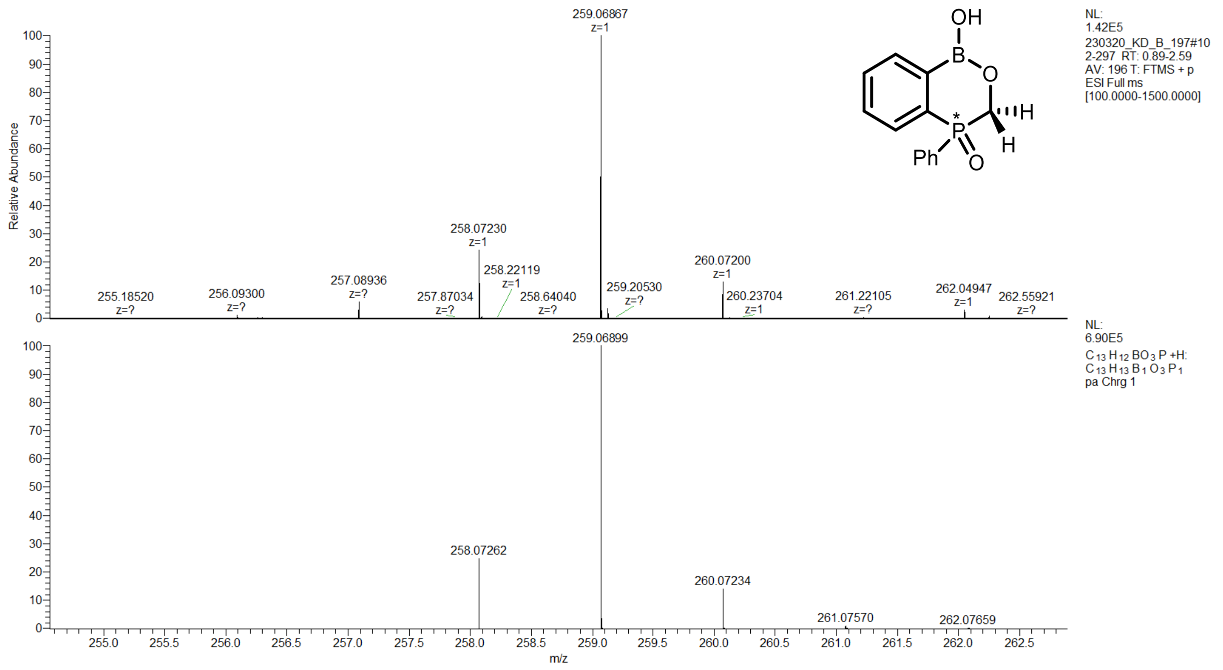

Figure S87. HRMS spectrum (ESI, positive ion mode) of **12**. The calculated spectrum of the formula $C_{13}H_{13}BO_3P^+$ $[M+H]^+$ is given in the bottom.

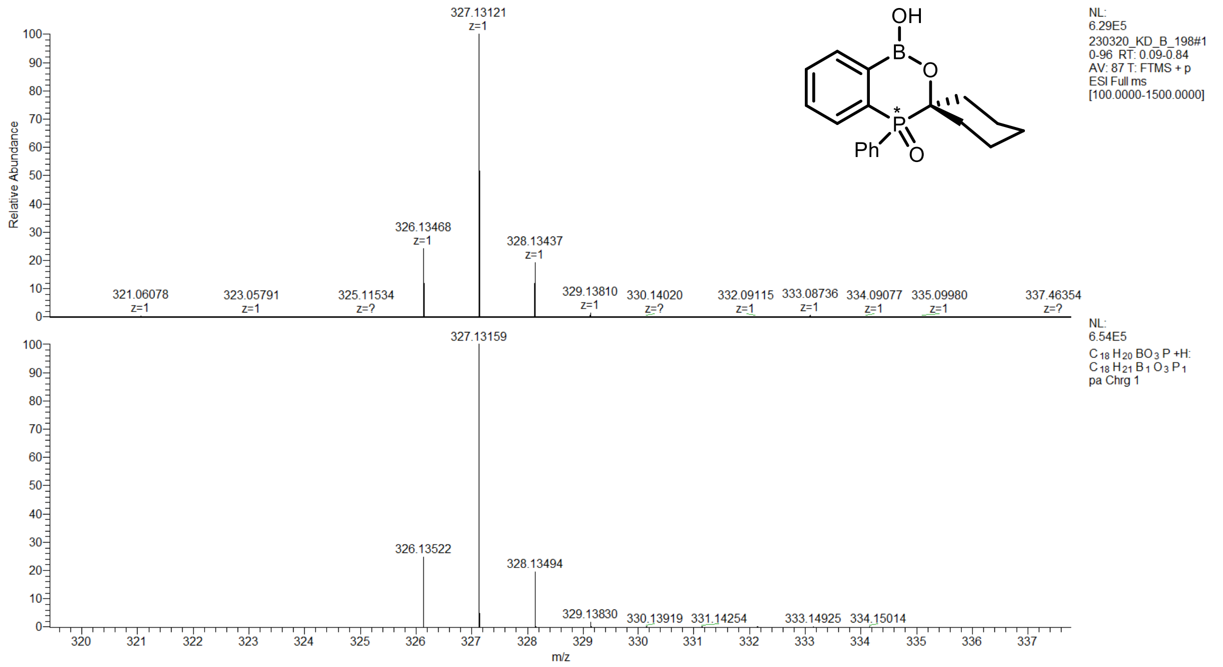

Figure S88. HRMS spectrum (ESI, positive ion mode) of **13**. The calculated spectrum of the formula $C_{18}H_{21}BO_3P^+$ $[M+H]^+$ is given in the bottom.

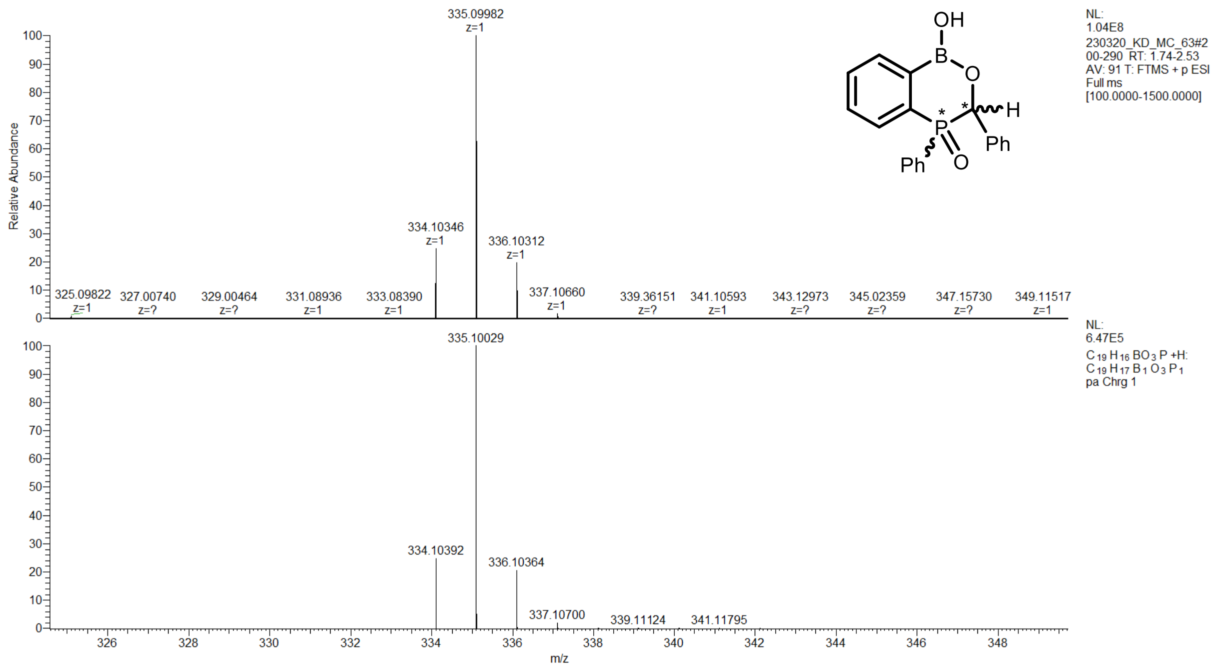

Figure S89. HRMS spectrum (ESI, positive ion mode) of **14**. The calculated spectrum of the formula $C_{19}H_{17}BO_3P^+$ $[M+H]^+$ is given in the bottom.

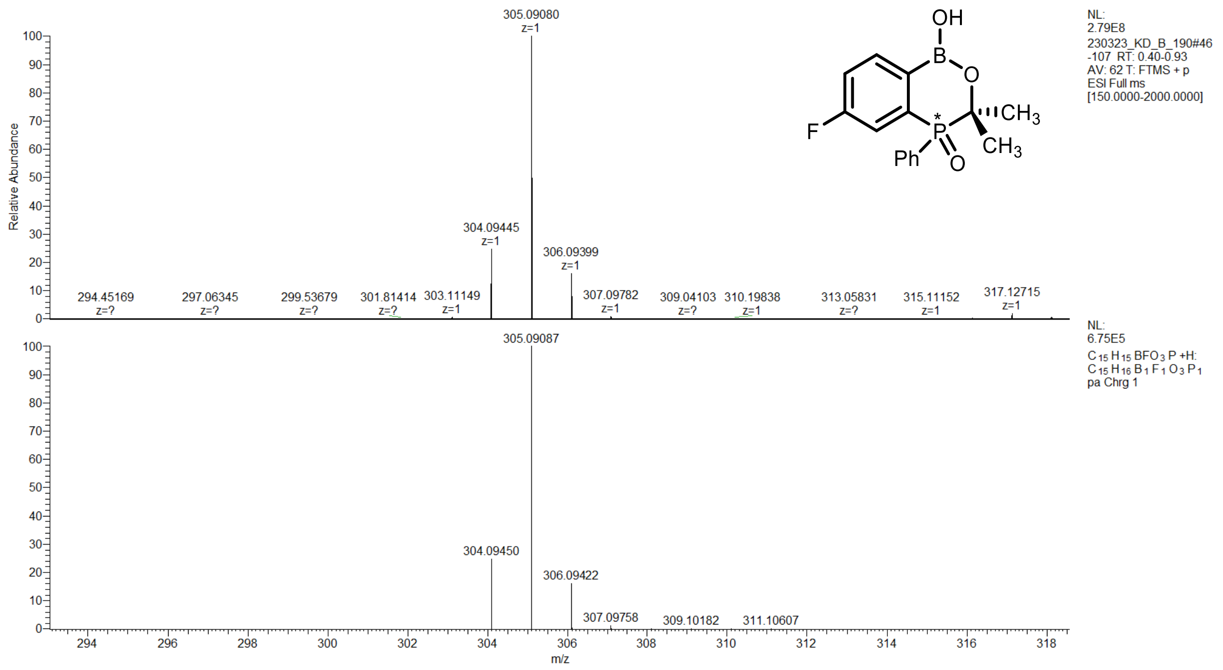

Figure S90. HRMS spectrum (ESI, positive ion mode) of **15**. The calculated spectrum of the formula $C_{15}H_{16}BFO_3P^+$ $[M+H]^+$ is given in the bottom.

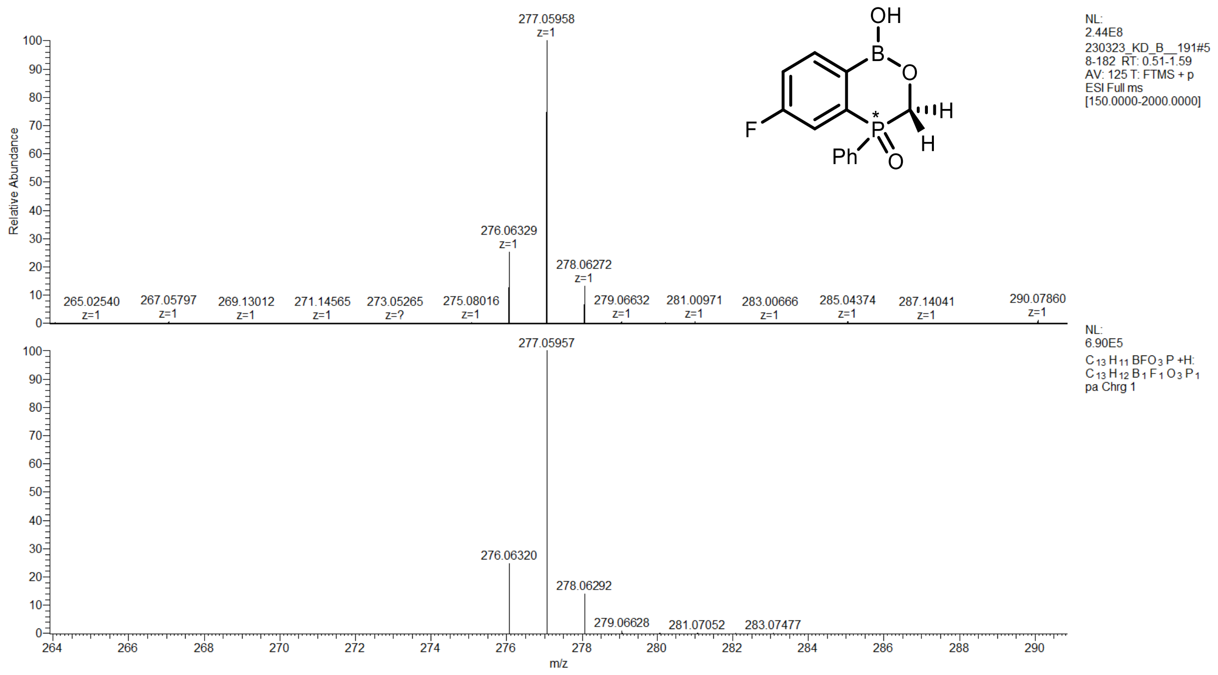

Figure S91. HRMS spectrum (ESI, positive ion mode) of **16**. The calculated spectrum of the formula $C_{13}H_{12}BFO_3P^+$ $[M+H]^+$ is given in the bottom.

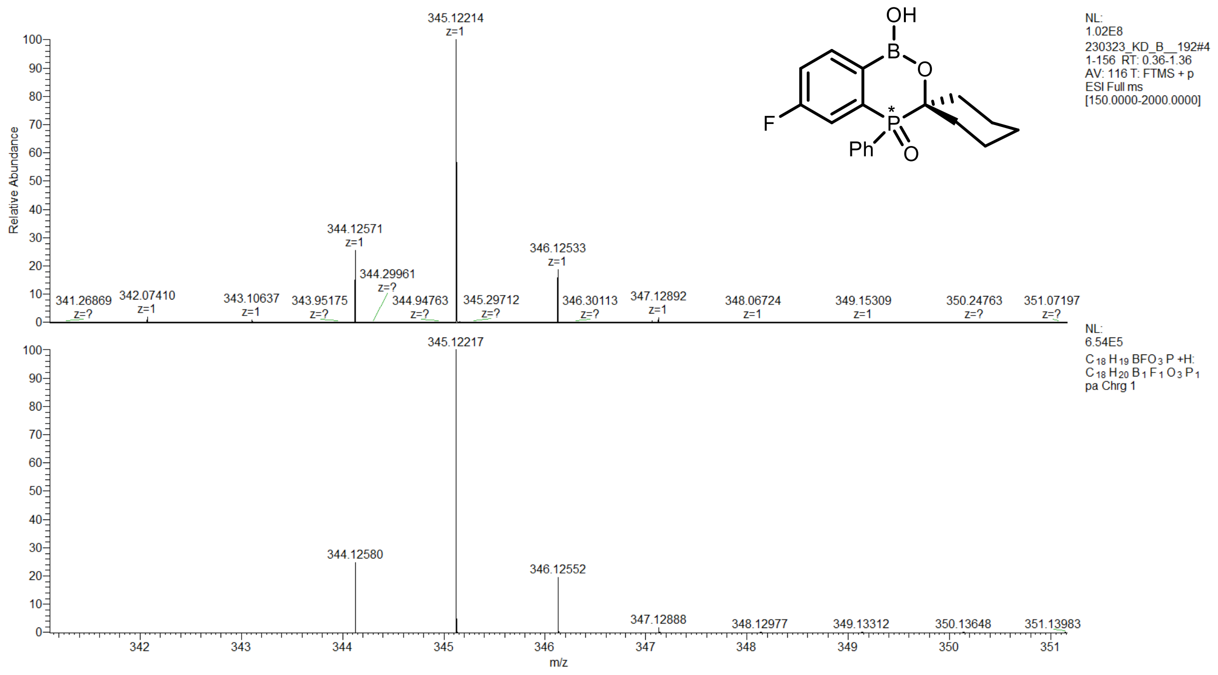

Figure S92. HRMS spectrum (ESI, positive ion mode) of **17**. The calculated spectrum of the formula $C_{18}H_{20}BFO_3P^+$ $[M+H]^+$ is given in the bottom.

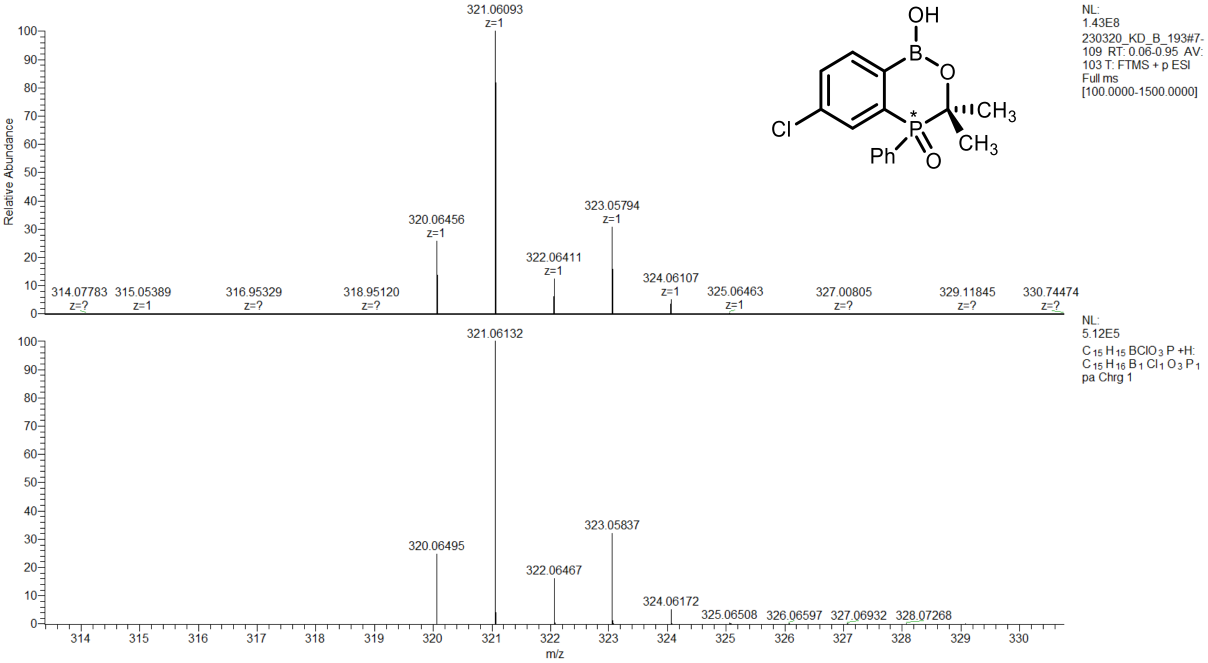

Figure S93. HRMS spectrum (ESI, positive ion mode) of **18**. The calculated spectrum of the formula $C_{15}H_{16}BClO_3P^+$ $[M+H]^+$ is given in the bottom.

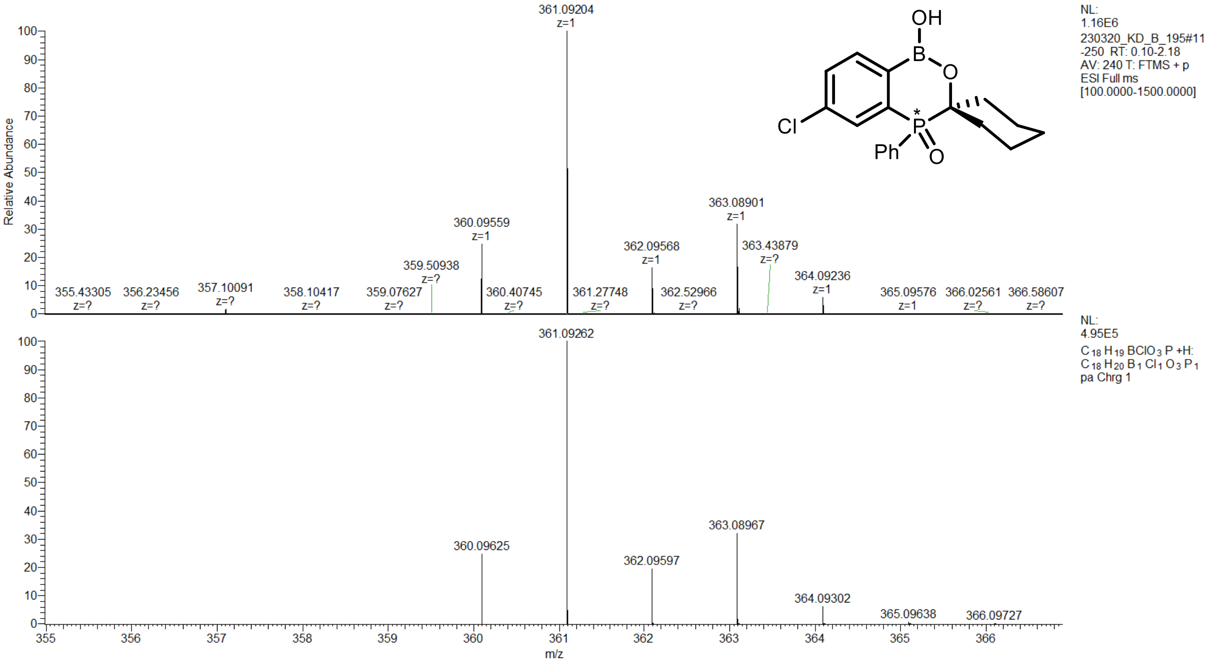

Figure S94. HRMS spectrum (ESI, positive ion mode) of **19**. The calculated spectrum of the formula $C_{18}H_{20}BClO_3P^+$ $[M+H]^+$ is given in the bottom.

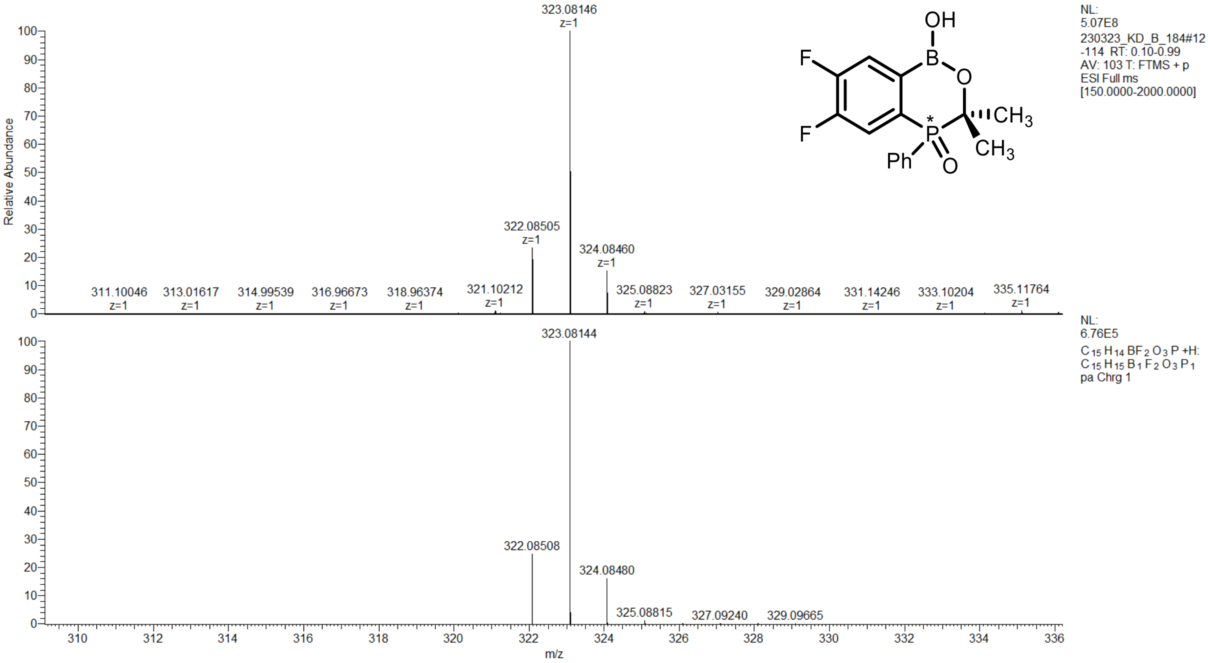

Figure S95. HRMS spectrum (ESI, positive ion mode) of **20**. The calculated spectrum of the formula $C_{15}H_{15}BF_2O_3P^+$ $[M+H]^+$ is given in the bottom.

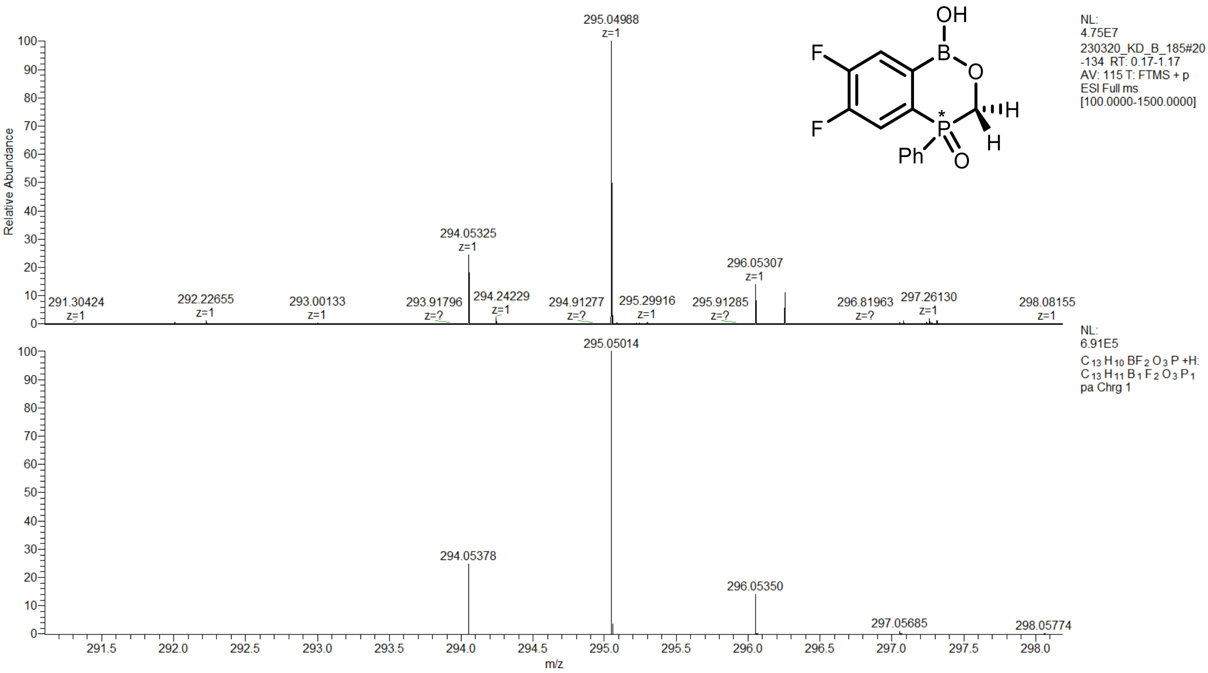

Figure S96. HRMS spectrum (ESI, positive ion mode) of **21**. The calculated spectrum of the formula $C_{13}H_{11}BF_2O_3P^+$ $[M+H]^+$ is given in the bottom.

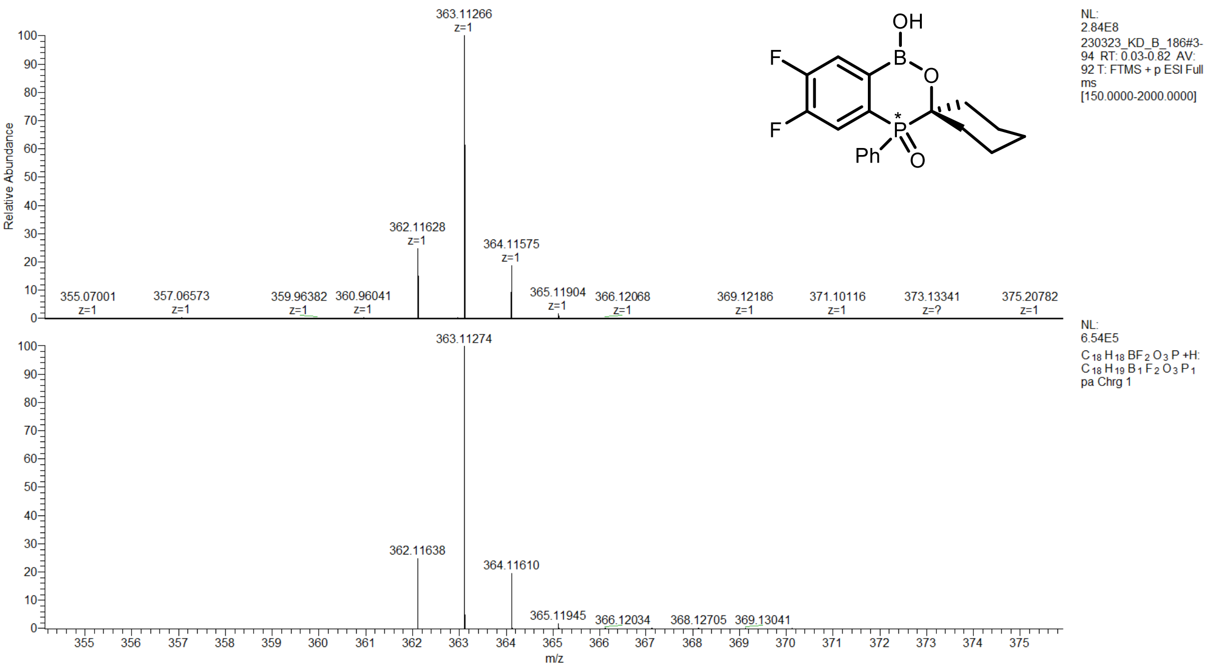

Figure S97. HRMS spectrum (ESI, positive ion mode) of **22**. The calculated spectrum of the formula $C_{18}H_{19}BF_2O_3P^+$ $[M+H]^+$ is given in the bottom.

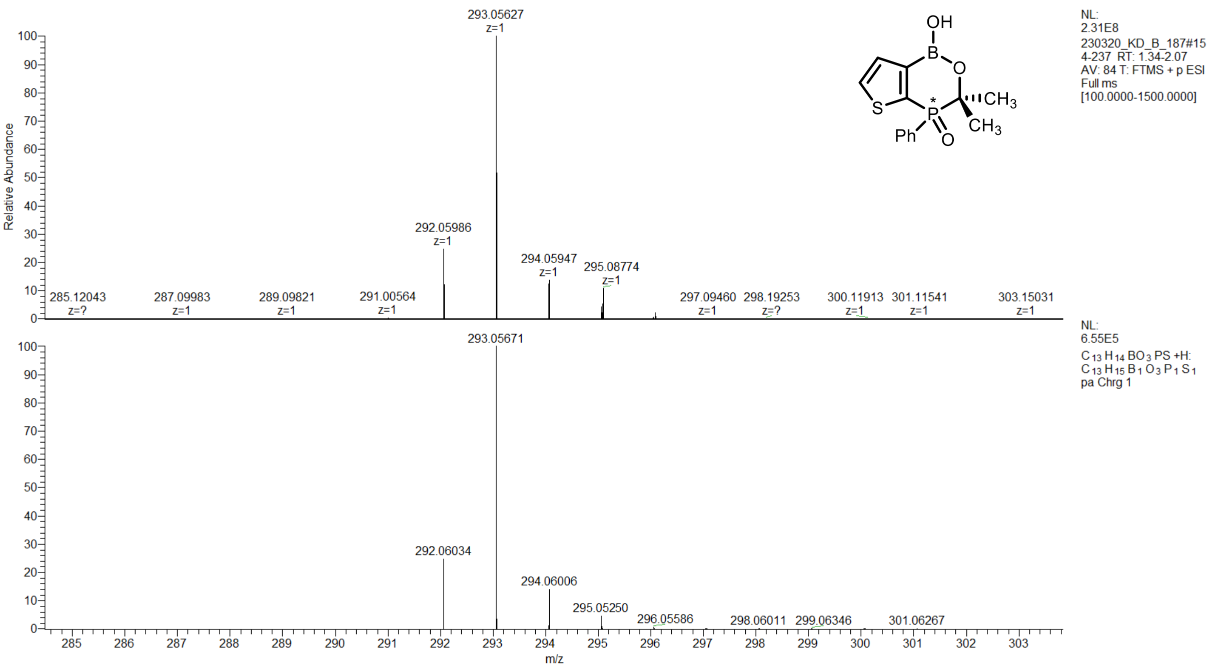

Figure S98. HRMS spectrum (ESI, positive ion mode) of **23**. The calculated spectrum of the formula $C_{13}H_{15}BO_3PS^+$ $[M+H]^+$ is given in the bottom.

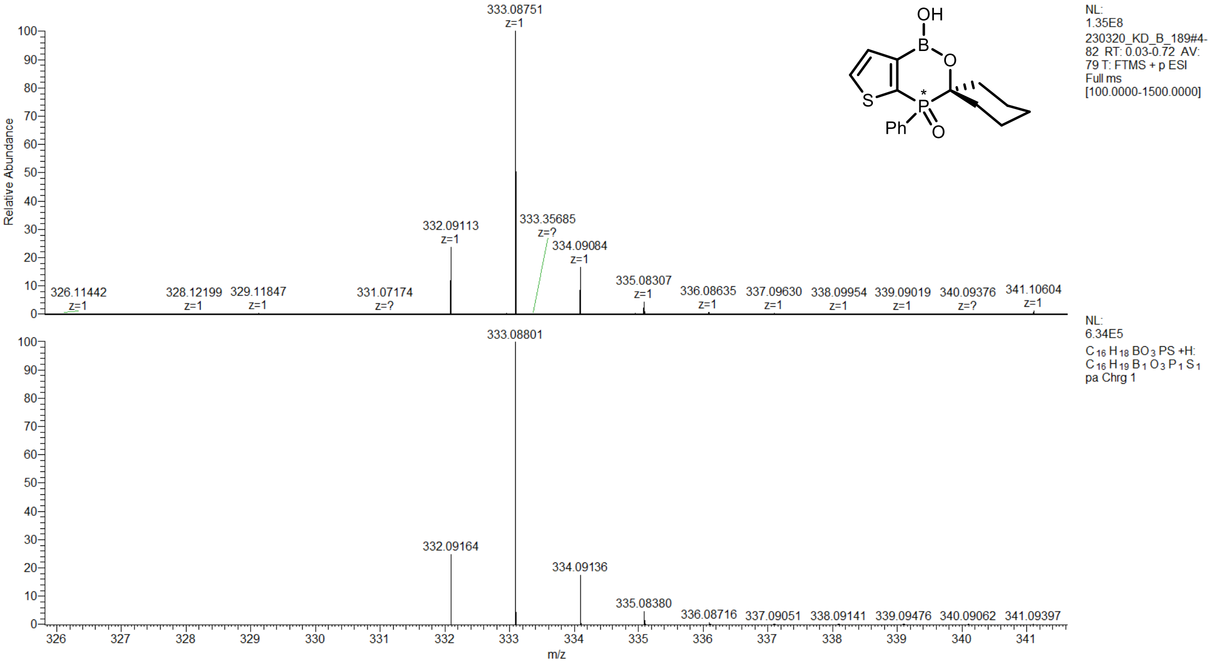

Figure S99. HRMS spectrum (ESI, positive ion mode) of **24**. The calculated spectrum of the formula $C_{16}H_{19}BO_3PS^+$ $[M+H]^+$ is given in the bottom.

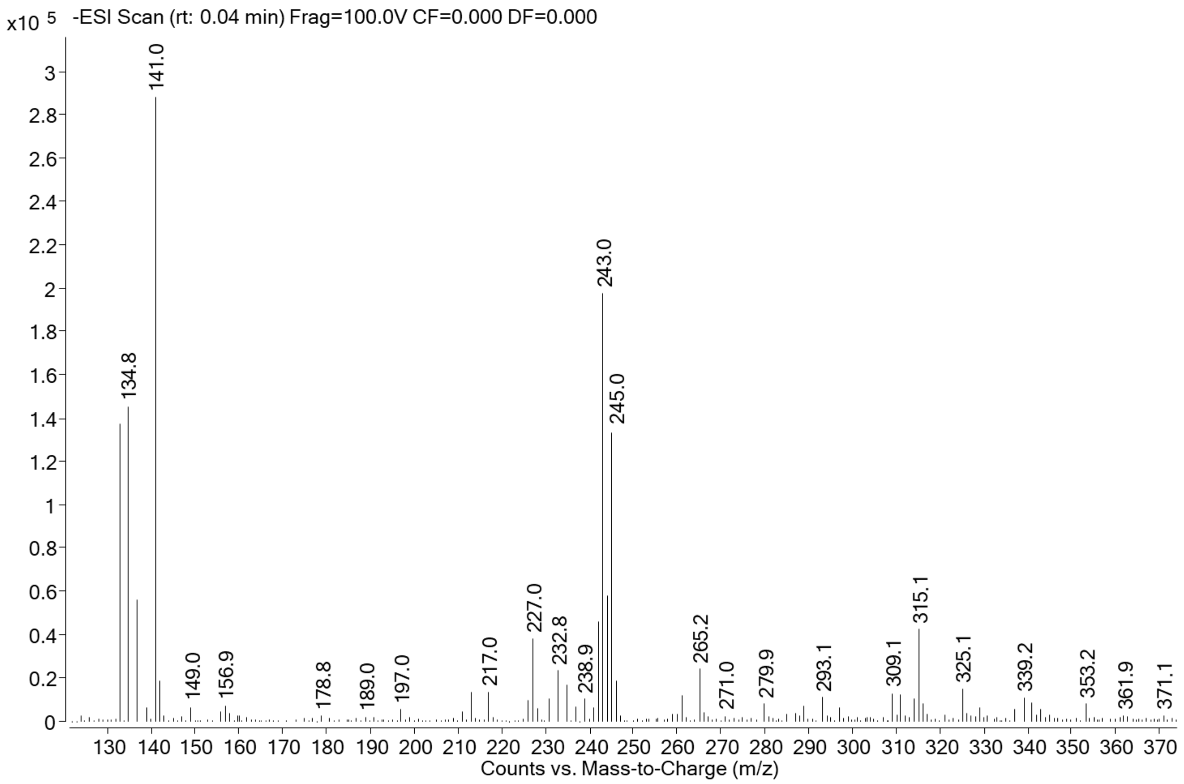

Figure S100. ESI MS scan spectrum (negative ion mode) of **5**, 50 ppm in 0.1% formic acid/acetonitrile 1:1 (*v*:*v*).

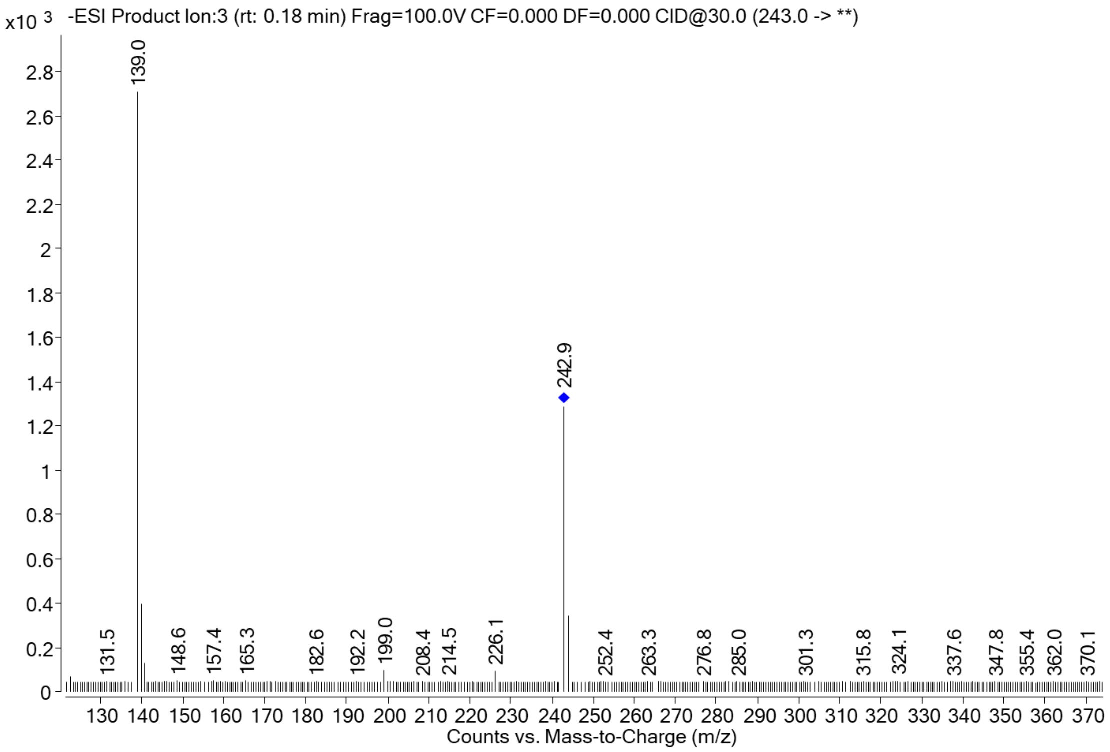

Figure S101. ESI MS/MS product ion spectrum (negative ion mode, CID = 30 V) of a signal 243 *m/z* corresponding to deprotonated **5-P(O)OB**, 50 ppm in 0.1% formic acid/acetonitrile 1:1 (*v*:*v*).

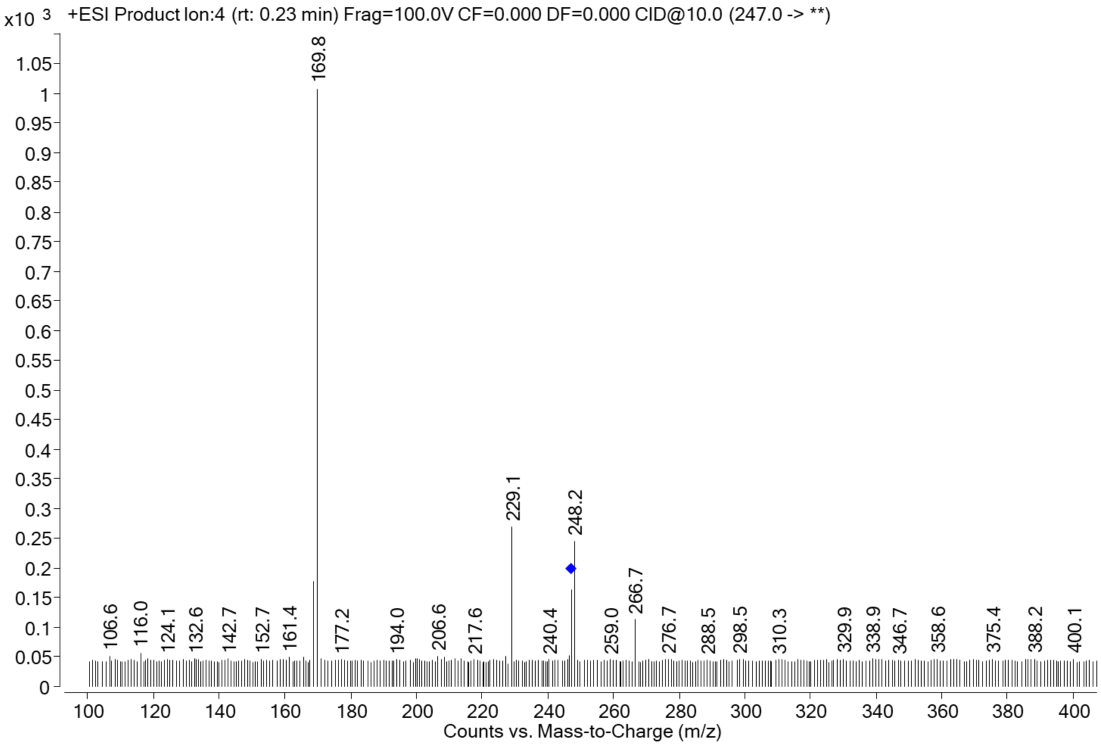

Figure S102. ESI MS/MS product ion spectrum (positive ion mode) of a signal 247 *m/z*, corresponding to protonated **5**, 50 ppm in 0.1% formic acid/acetonitrile 1:1 (*v*:*v*).

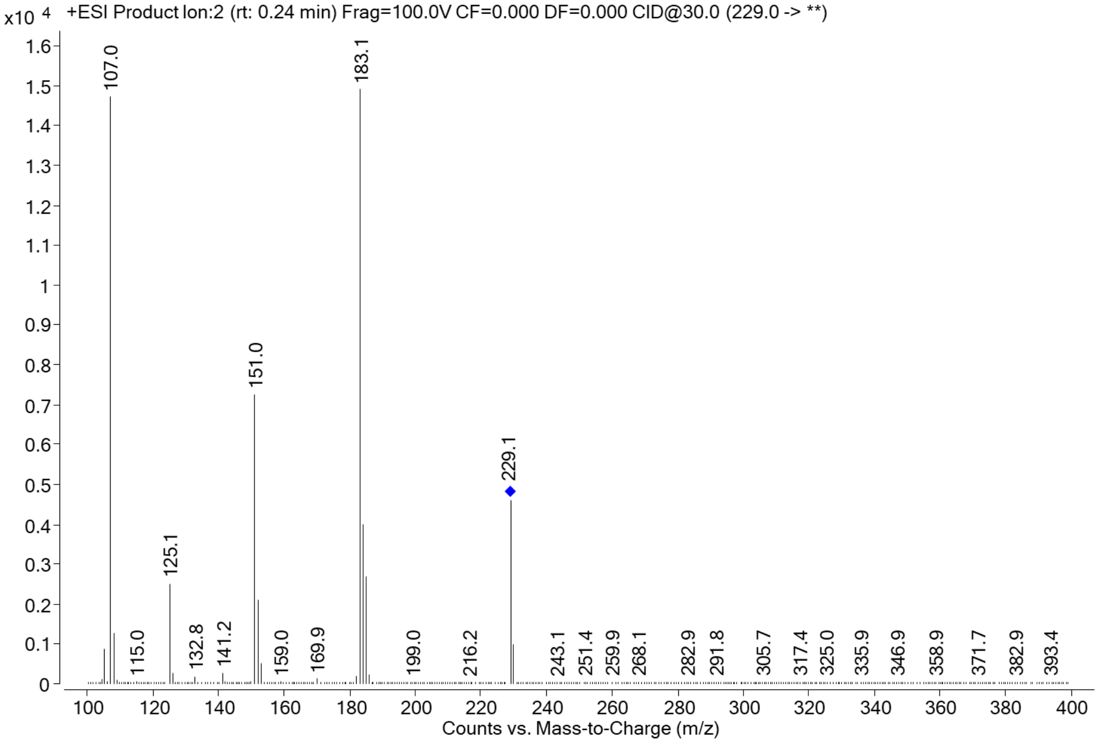

Figure S103. ESI MS/MS product ion spectra (positive ion mode) of a signal 229 *m/z*, corresponding to protonated **5-POB**, 50 ppm in 0.1% formic acid/acetonitrile 1:1 (*v*:*v*)

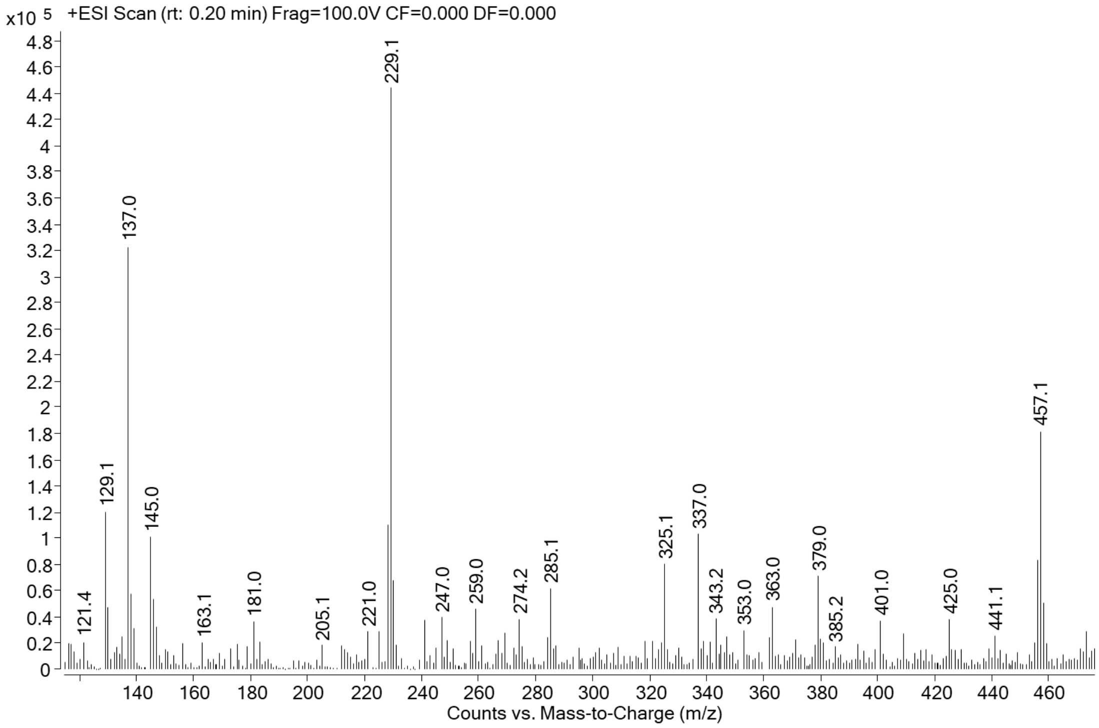

Figure S104. ESI MS scan spectrum (positive ion mode) of **5**, 50 ppm in 0.1% formic acid/acetonitrile 1:1 (*v*:*v*), showing the signal 457 *m/z* corresponding to protonated dimer of **5-POB**.
